# Neandertal fire-making technology inferred from microwear analysis

**DOI:** 10.1038/s41598-018-28342-9

**Published:** 2018-07-19

**Authors:** A. C. Sorensen, E. Claud, M. Soressi

**Affiliations:** 1 Faculty of Archaeology, Leiden University, Leiden, The Netherlands; 2 INRAP, GSO, Bègles, France; 3 UMR 5199 PACEA, Université de Bordeaux, Pessac, France

## Abstract

Fire use appears to have been relatively common among Neandertals in the Middle Palaeolithic. However, the means by which Neandertals procured their fire—either through the collection of natural fire, or by producing it themselves using tools—is still a matter of debate. We present here the first direct artefactual evidence for regular, systematic fire production by Neandertals. From archaeological layers attributed to late Mousterian industries at multiple sites throughout France, primarily to the Mousterian of Acheulean Tradition (MTA) technoculture (ca. 50,000 years BP), we identify using microwear analysis dozens of late Middle Palaeolithic bifacial tools that exhibit macroscopic and microscopic traces suggesting repeated percussion and/or forceful abrasion with a hard mineral material. Both the locations and nature of the polish and associated striations are comparable to those obtained experimentally by obliquely percussing fragments of pyrite (FeS_2_) against the flat/convex sides of a biface to make fire. The striations within these discrete use zones are always oriented roughly parallel to the longitudinal axis of the tool, allowing us to rule out taphonomic origins for these traces. We therefore suggest that the occasional use of bifaces as ‘strike-a-lights’ was a technocultural feature shared among the late Neandertals in France.

## Introduction

Most people today are familiar with the concept of striking steel against flint to produce a shower of sparks that fall onto tinder, which begins to smoulder, and when placed into a bundle of dried grass, can be gently blown into flame. Prior to cigarette lighters and wooden matches, the flint-and-steel method was among the most common fire making systems in modern times, originating in the Iron Age^1–3^. Prior to their introduction to metal products by Western colonialists, numerous hunter-gatherer, pastoralist and horticulturalist societies employed the minerals pyrite or marcasite—two very similar species of iron disulphide (FeS_2_) that are virtually indistinguishable from one another in their nodular or crystal aggregate forms, referred to hereafter simply as pyrite—in place of steel for their fire making needs. Ethnographic accounts describe the flint-and-pyrite (and in some cases, pyrite-on-pyrite) fire making system being employed from Alaska and Canada to Tierra del Fuego in the Americas, and from Australia and Melanesia to Siberia, and only a few instances noted in Africa^1,4,5^. However, the earliest known instances of percussive fire making extend much deeper into the prehistoric past.

## Archaeological evidence for fire making

Strike-a-lights (or *briquets*, in French)—the usual term for the flint element in the flint and pyrite fire making system—and pyrite have been recovered archaeologically from Palaeo-Eskimo contexts on Greenland^6,7^ and in Alaska^8^, and at numerous Bronze Age, Neolithic and Mesolithic sites throughout Eurasia^5,9–13^. However, comparatively speaking, very few fire making tools have been recovered from earlier, i.e. Palaeolithic, contexts^6,14–16^.

The paucity of fire making tools during the Palaeolithic may be due to both taphonomic and behavioural variables. Multiple pieces of pyrite have been recovered from Palaeolithic contexts, with a few Upper Palaeolithic (hereafter, UP) examples exhibiting traces of use consistent with fire making^5,17,18^ (see^14^ for a comprehensive list of known Palaeolithic pyrite specimens). However, due to a corrosive phenomenon called ‘pyrite decay’^19^, it is likely that far more pieces have disintegrated after having been discarded. This reaction occurs when iron sulphide minerals like pyrite and marcasite oxidize and degrade upon exposure to humid air^20^. This may also be why it is rare to find pyrite residue adhering to ancient strike-a-lights, the oldest exhibiting overt, well-preserved pyrite residues being a set of eight Neolithic examples from Switzerland^21^, and a few more ambiguous late UP specimens from the Netherlands and Denmark^6^.

Furthermore, the scarcity of evidence for fire making in the Middle Palaeolithic (hereafter, MP) and UP may also be due to the nature of the flint tools used. It has been postulated that fire making during these periods may have been performed using less visible, more expedient tools^14,22,23^. This means fire making likely did not involve formalised strike-a-light tool types used for extended periods of time, but was instead performed using flint fragments (e.g. flakes, cores, debitage, etc.) readily available in the vicinity, recycled tools originally used for other tasks, or multi-purpose tools utilised for any number of tasks—anything, so long as the tool in question was considered by the user suitable for the task. In each case, it is likely the tool was used as a fire maker only once, or perhaps a handful of times at most. This has two major implications: 1) fire making tools are not immediately recognisable by their morphology, and 2) physical evidence of these tools having been used to make fire (i.e. use-wear) may not be readily apparent without the help of more detailed microscopic analysis. This makes identifying expedient fire making tools potentially very difficult and could be why so few are known from these early periods.

## Where are the Middle Palaeolithic strike-a-lights? The biface hypothesis

While it is generally assumed that modern humans were proficient fire makers, some researchers doubt Neandertals knew how to artificially make fire^24,25^ despite evidence that they used fire regularly^26–28^. Since using fire does not necessarily require the ability to produce fire (natural fires in the landscape may have provided semi-regular access to this resource in the past), only by identifying the tools used to make fire can we know if Neandertals possessed this skill. Manganese dioxide (MnO_2_)—a black mineral that when powdered and added to woody material lowers its combustion temperature by around 100 °C—was collected by late Neandertals and may have been used as a tinder-enhancer for fire making^29^. Given that fire cannot be made from MnO_2_ alone, more evidence is needed to firmly establish that Neandertals were able to produce fire. To date, only one tool from a Neandertal site (Bettencourt, France) has been interpreted as a strike-a-light^16,30,31^. This piece, like others recovered from late UP contexts, bears relatively weak use damage on the surface of the tools compared to their younger Neolithic and Bronze Age counterparts, suggesting they may have been expedient tools^14^.

The vast majority of known prehistoric strike-a-lights tend to be elongated pieces with the active zones (i.e. the portion of the tool used to strike the pyrite) positioned at one or both ends of the tool^6,30^. While this appears to be the norm, this pattern could largely be an example of sampling bias; that is, since most Stone Age peoples from the UP onward employed elongated blade-based lithic technologies as the basis for most of their stone tools, it only makes sense that this be the case for strike-a-lights, as well. Why should we then expect this pattern to hold for non-blade-based flake-tool industries, like those generally employed during the MP (e.g. Levallois, discoid), or assemblages rich in large bifacially-flaked tools (sometimes referred to as ‘handaxes’) like those that typified the Lower Palaeolithic Acheulean, or the late MP Mousterian of Acheulean Tradition (hereafter, MTA) technoculture?

Bifaces are usually seen as curated tools that were often transported over large distances in the MP and used for relatively long periods of time, as demonstrated specifically for MTA bifaces^32–36^. We postulate that curated tools such as these possess a higher probability of preserving traces from multiple use activities—some perhaps infrequent—than any one short-term use flake tool. While primarily used for animal butchery, late MP bifaces were also used for other tasks, including working mineral resources^37–41^. It is possible that some of these mineral use traces could be the result of percussive fire making by Neandertals^42^.

**Figure 1:**
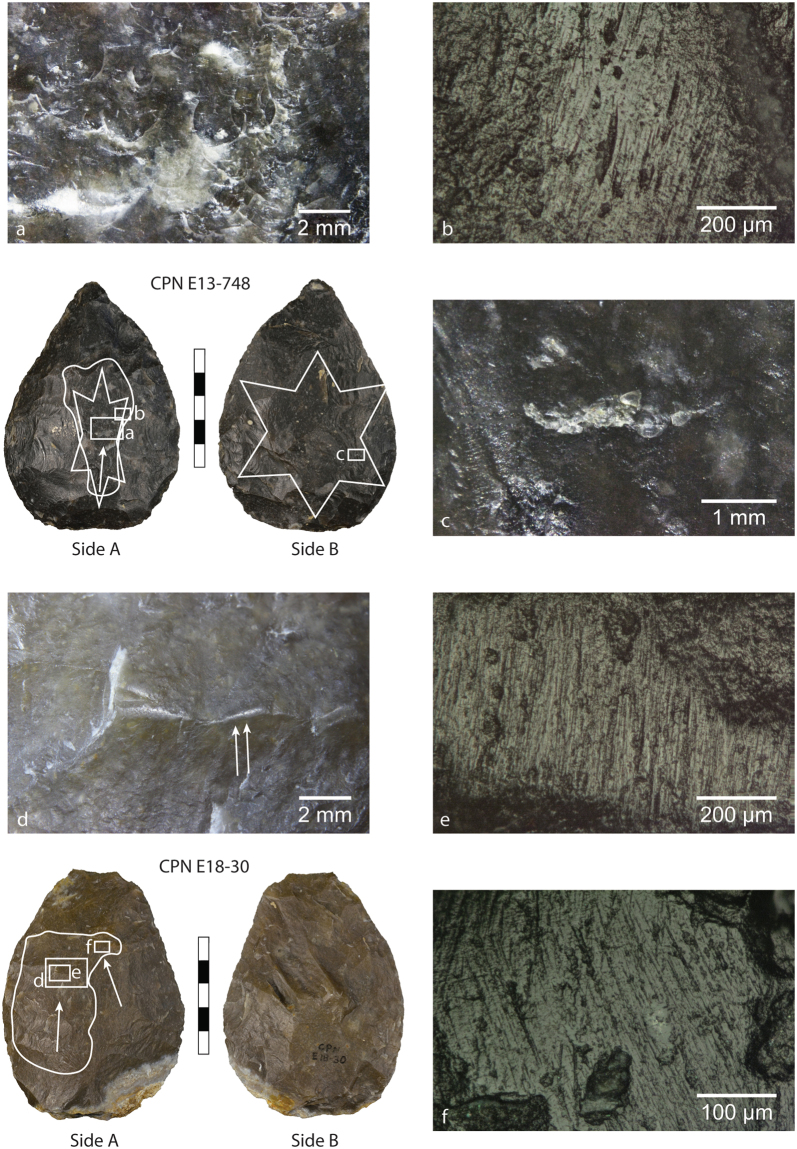
Images of mineral use-wear traces on archaeological bifaces CPN E13-748 (top) and CPN E18-30 (bottom) from Chez-Pinaud/Jonzac (Charente-Maritime). The white lines demarcate the zone of mineral use-wear traces comparable to pyrite. The arrows indicate the orientations of associated striations. The star on Side A of CPN E13-748 indicates a zone of percussion containing numerous C-shaped percussion marks that open distally (**a**) in good agreement with the striations (**b**). On Side B, the star encompasses a zone of percussion containing multiple linear gouges (**c**) indicating this surface was used for retouching/flintknapping. A low-magnification image of the surface of CPN E18-30 (**d**) shows the extent of ridge rounding. The arrows in this image indicate two small (difficult to see) distally opening percussion marks. (**d**) High-magnification image of planed flake scar ridge with well-developed mineral polish and striations. (**f**) High-magnification image of well-developed mineral polish and intersecting striations of different directionalities, possibly indicating more than one use episode.

**Figure 2:**
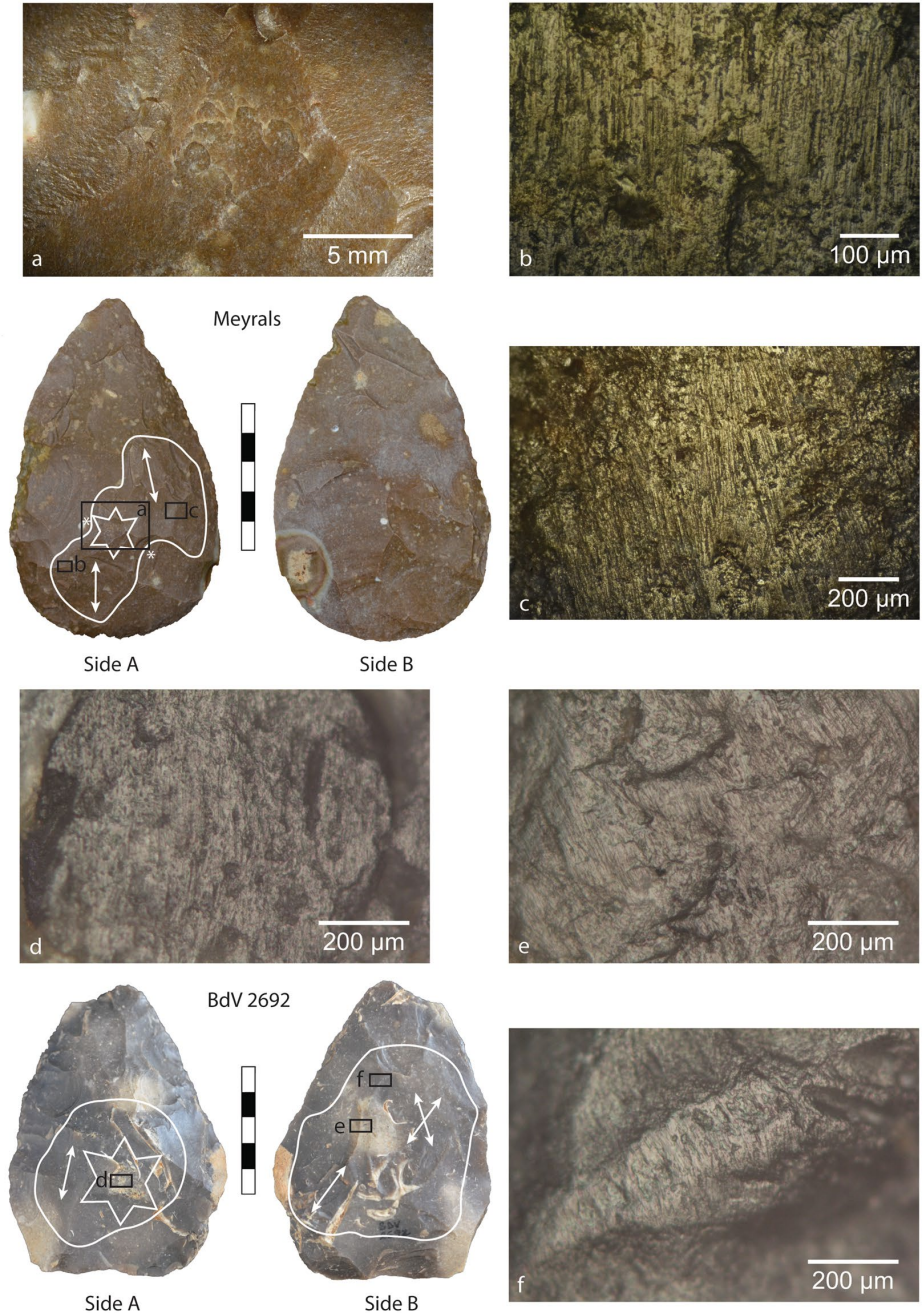
Images of mineral use-wear traces on an archaeological biface from Meyrals (top) and biface BdV 2692 from Bous-des-Vergnes (bottom), both situated in the Dordogne. The white lines demarcate the zones where mineral use wear traces comparable to pyrite are present. The arrows indicates the orientation of striations. The star on the Meyrals bifaces delineates a zone of percussion marks with ambiguous directionalities (**a**), though the majority open proximally, while the asterisks flanking the star indicate zones of percussion marks that have been truncated by subsequent flake removals (as seen more clearly in the left flake negative in image **a**). (**b**,**c**) High-magnification images of mineral microwear polish and striations showing slightly variable directionalities, possibly indicating at least two use episodes. For BdV 2692, the star on Side A indicates the primary zone of percussion and heavy crushing, though percussion marks are present throughout use zone. (**d**) High-magnification image of mineral microwear traces within a percussion mark fracture on Side A. (**e**) High-magnification image of mineral microwear traces with striations showing intersecting directionalities, suggesting more than one use episode on Side B. (**f**) High-magnification image of mineral microwear traces on flake scar ridge.

**Figure 3:**
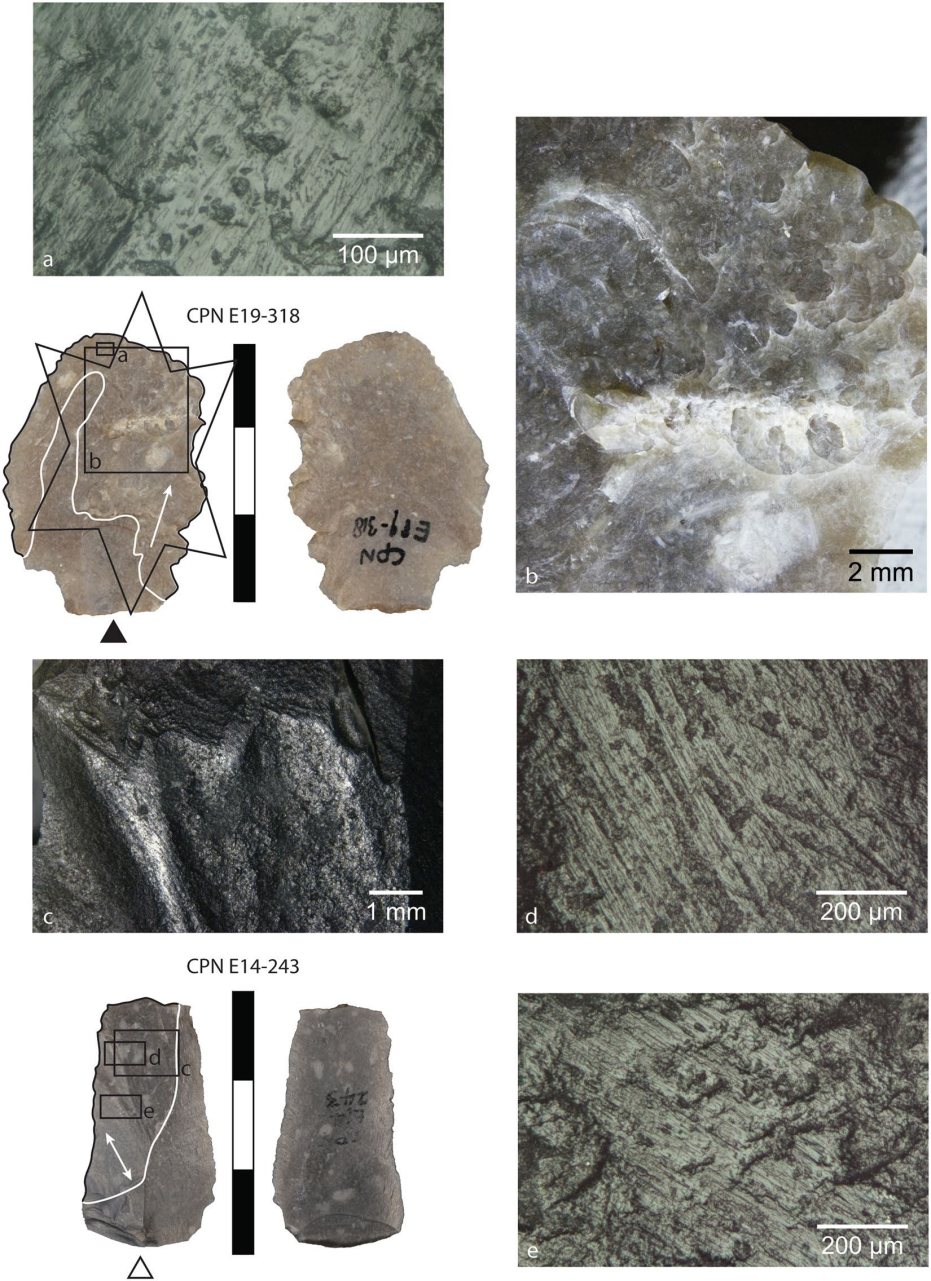
Images of mineral use-wear traces on an archaeological bifacial thinning flakes CPN E19-318 (top) and CPN E14-243 (bottom) from Chez-Pinaud/Jonzac (Charente-Maritime). The white and black lines demarcate the zone of mineral use wear traces comparable to pyrite. The arrows indicate the orientation of the striations. The star on CPN E19-318 indicates these traces are located within a zone of heavy percussion and crushing (**b**), the percussion mark directionalities being somewhat variable, though many open distally in agreement with the striations. (**a**) High-magnification images of well-developed mineral polish and striations on CPN E19-318. (**c**) Low-magnification image of the surface of CPN E14-243 highlights the heavy rounding of flake scar ridges. (**d**,**e**) High-magnification images of well-developed mineral polish, striations and slightly wider and deeper surface scratches.

Of the mineral use-wear traces that have previously been identified on some of these MTA artefacts (Supplementary Tables S1 and S2), we are chiefly interested in the those exhibiting on their flat/convex faces directional percussive and frictive traces originally described by Claud^37,38,41^ as unidentified abrasive mineral use traces that manifest as visible rounding of flake scar ridges and/or percussion marks (i.e. C-shaped or circular impact points) associated with prominent microscopic (sub)parallel striations and polish (Fig. 1). These zones of use 1) may include only friction traces, percussion traces or both, 2) are variably located, i.e. on the proximal and/or distal ends on one of both sides of any given biface, and 3) are present on bifaces at various stages of their use-lives, i.e. on larger, only slightly reduced bifaces and on smaller, more heavily denatured bifaces. However, the striations, when present, occur in discrete zones (as opposed to being evenly distributed across the surface of the biface) and are consistently oriented parallel to the longitudinal axis or to a lateral edge of the bifaces^37^, regardless of location, and are sometimes cross-cut by later flake removals (Fig. 2), either from the resharpening of the biface or from using the biface as a flake core. These points, along with the fact that these traces have, to our knowledge, only been observed on bifaces—as well as on bifacial thinning flakes produced during the shaping of a biface (Fig. 3)—within MTA assemblages (despite other lithic elements like scrapers and flakes having also been analysed), indicate that these marks result from deliberate actions by Neandertals and not from post-depositional processes. Moreover, previous microwear analyses of these tools have not determined a link between the mineral use-wear and the use traces associated with other activities (e.g. butchery, hide processing, wood working, etc.), suggesting the mineral use traces are their own entity^37,38,41^. Other observed microwear traces (when present) are indicated in the Supplementary Information figures and listed in Supplementary Table S1.

**Figure 4:**
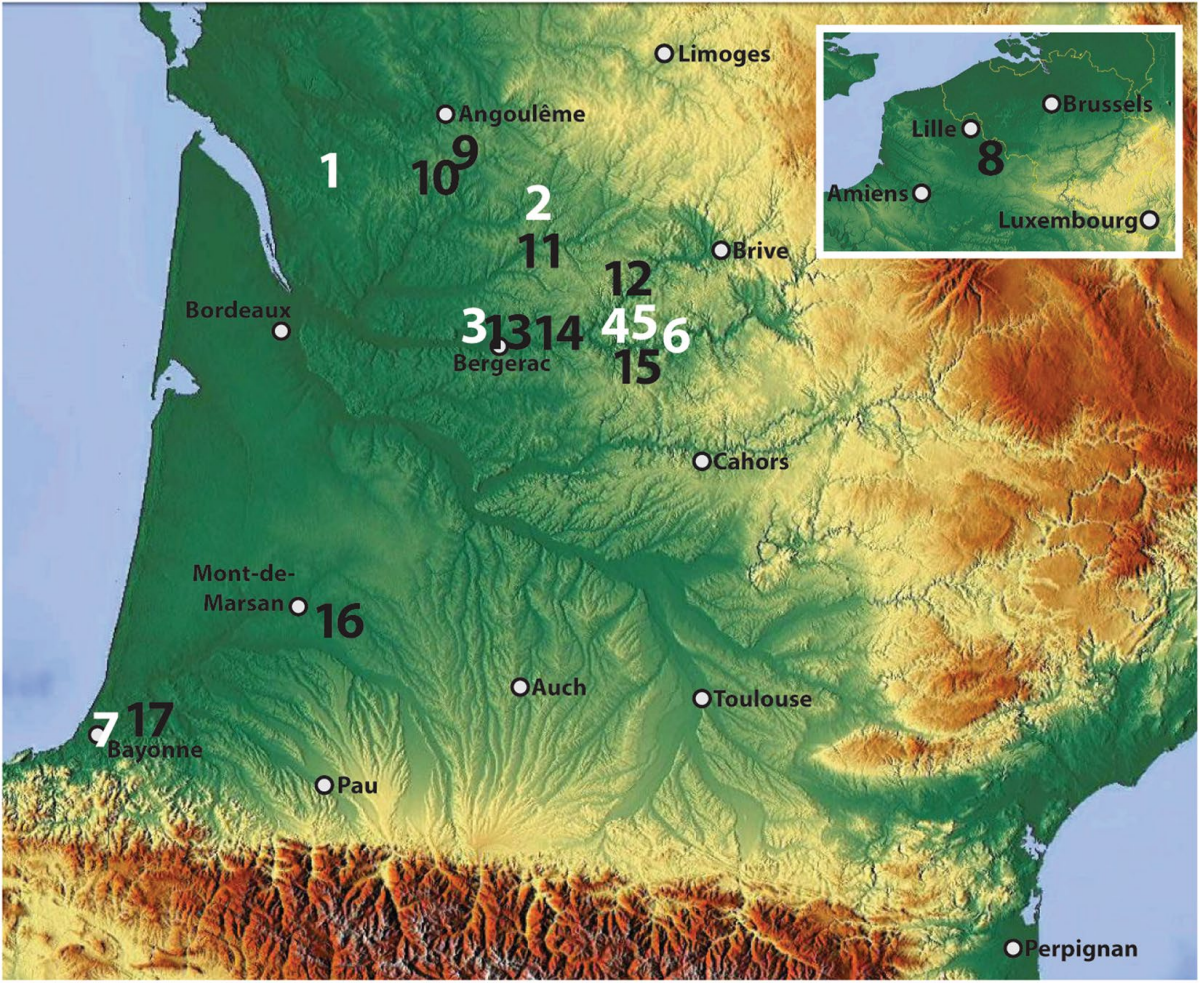
Map of southwest France with locations of sites discussed in text. Inset map includes northern France and Belgium. Bifaces from sites with white numbers (1–7) were analysed for this study (Table S1), while sites with black numbers (8–17) are known to possess bifaces with mineral traces, but were not analysed for this study (Table S2). Chez-Pinaud/Jonzac (1), Fonseigner (2), Bout des Vergnes (3), Meyrals (4), Sarlat (5), Pech de l’Azé I (6), Le Prissé (7), Bas-du-Mont des Bruyères (Saint-Amand-les-Eaux) (8), La Quina (9), Les Bessinaudes (10), Coursac (11), La Rochette (12), Canolle (13), Les Vieux Coutets (14), Grotte XVI (15), Latrote (16), Le Chemin de Jupiter (17).

We utilise a microwear analysis-based approach to test the hypothesis that at least some of these previously identified mineral wear traces are the result of using the bifaces as strike-a-lights. A battery of experiments (Supplementary Table S3) were performed using 32 surfaces on 8 replica flint bifaces and 4 scraper tools in conjunction with pyrite and other hard mineral materials to 1) test the efficacy of using bifaces to make fire, 2) to compare the mineral use-wear produced to one another to determine if similarities exist that could cause ambiguity, and 3) compare the traces produced to those observed on the archaeological specimens. A selection of late MP bifaces from France, primarily from assemblages attributed to the MTA, were included in our analysis, including specimens from Chez-Pinaud/Jonzac (hereafter, CPN; Charente-Maritime)^38,41^, Le Prissé (Pyrénées Atlantiques)^39,40^ and Pech de l’Azé I (hereafter, Pech I)^43^, Bout-des-Vergnes (hereafter, BdV)^40,44^, Fonseigner^32,41^, Sarlat and Meyrals (unpublished findspots; A. Turq, pers. comm.), all located in the Dordogne (Fig. 4; Supplementary Table S1; for other sites where bifaces exhibiting mineral use-wear have been recovered, see Supplementary Table S2). We identify multiple isolated zones of macroscopic and microscopic traces suggesting repeated percussion and/or forceful abrasion with a hard mineral material and compare these to traces obtained experimentally through percussive and abrasive tasks involving various stony materials, including fire making using fragments of pyrite^6,14,15,23,45^.

It should be noted that no pyrite residues were observed on the archaeological pieces during analyses using an optical microscope, so no systematic residue analyses were performed for this study. The presence of pyrite residues in intimate association with fire making microwear traces was confirmed on a series of Neolithic strike-a-lights using micro X-ray fluorescence (μ-XRF), RAMAN spectroscopy and micro X-ray diffraction (μ-XRD)^21^, but these methods were only used to confirm the nature of these residues since they were readily visible macroscopically. Another study utilized scanning electron microscopy (SEM) coupled to a spectrometer as a prospection method for identifying trace amounts of optically invisible pyrite residues on late UP strike-a-lights^6^. A few minute particles containing iron and sulphur atoms were observed on three of these tools, although it is possible these elements could be naturally derived from the encasing sediments, so their origins remain uncertain. While these results are promising, the tools examined for the current study are upwards of an order of magnitude older than those analysed in the above studies. Thus, the potential for using these analytical techniques to identify optically invisible pyritic micro-residues on tools of such great antiquity needs to be explored further given the very low probability for pyrite residue preservation.

## Results

Distinguishing between traces created by different mineral materials can indeed be challenging, especially between those with similar physical properties like hardness, crystal habits, fracturing tendencies, etc.^15,41,46–49^. Variability in the appearance of traces produced by the same contact material can complicate their assessment both on experimental and archaeological specimens and may be caused by a number of factors: variability between individual rock/mineral types (size, structure, contact surface morphology, etc.), a contact material behaving differently on different types of flint, variable preservation conditions, or that each archaeological biface was employed in a different series of functions after the mineral use traces were imparted^50^.

### Experimental results

**Figure 5:**
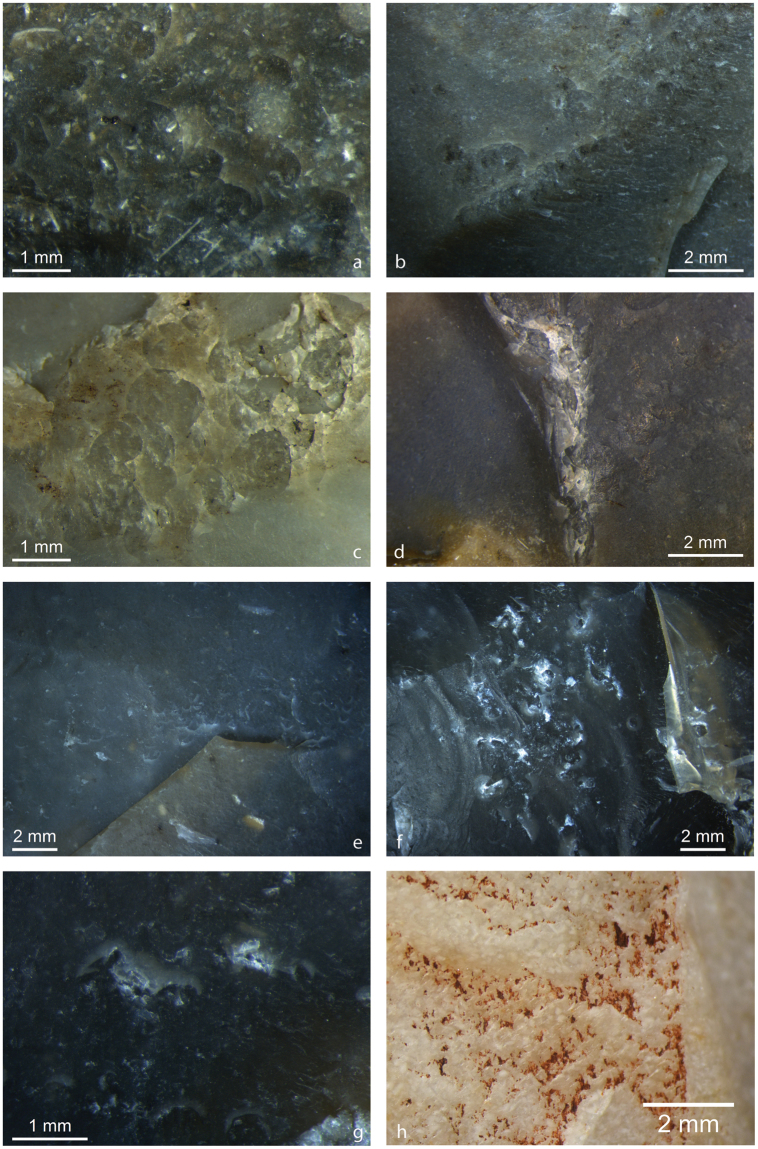
Images of experimental wear traces at low-magnification. (**a**) Unidirectional C-shaped percussion marks produced while making fire with pyrite (Exp 3471, Supplementary Fig. S39); (**b**) unidirectional C-shaped percussion marks clustered along a flake scar ridge while making fire (Exp 3474-Zone D, Supplementary Fig. S44); (**c**) percussion marks and heavy crushing produced during fire making (Exp 3470, Supplementary Fig. S38); (**d**) crushing and percussion marks along flake scar ridge produced during fire making (Exp 3472, Supplementary Fig. S40); (**e**) very small unidirectional C-shaped percussion marks produced while ‘backing’ a flint flake, caused by the sudden change in relief as the flake passed over the step-fracture and dropped onto the lower surface (Exp 3473-Zone B, Supplementary Fig. S41); (**f**) percussion marks and linear and ovate surficial gouges produced while flintknapping another flint biface (Exp 3476-Zone A, Supplementary Fig. S47); (**g**) percussion marks and linear surficial gouges produced while retouching the edge of a scraper (Exp 3476-Zone F, Supplementary Fig. S48); (**h**) iron-oxide mineral residue (after cleaning) deposited while abrading/grinding iron-cemented sandstone (Exp 3477-Zone D, Supplementary Fig. S50).

**Figure 6:**
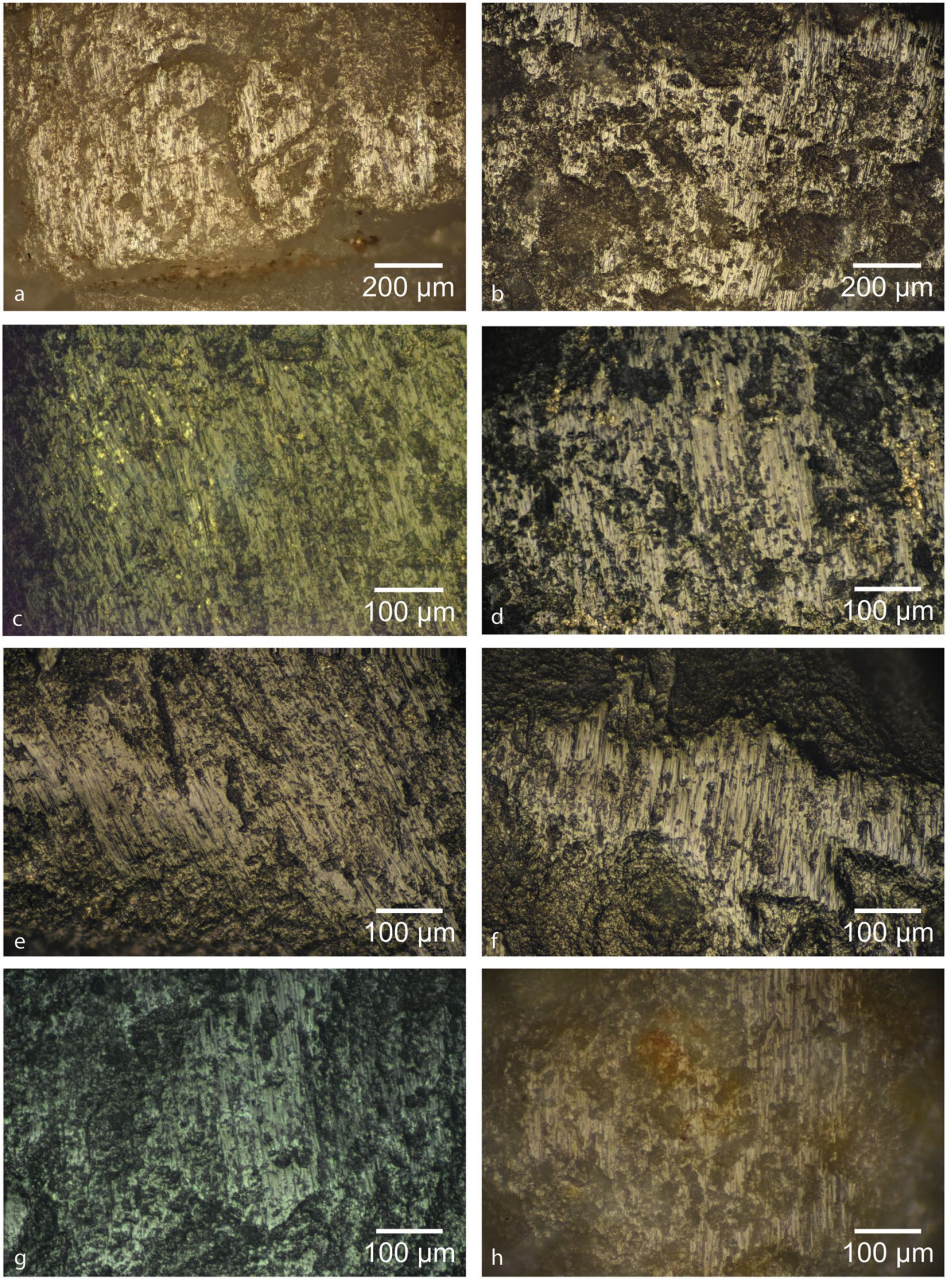
Images of experimental pyrite microwear traces at high-magnification. These traces generally occur as zone of matte, rough polish containing densely packed clusters of parallel to sub-parallel striations and scratches. (**a**) Exp 3470 (Supplementary Fig. S38), (**b**) Exp 3471 (Supplementary Fig. S39), (**c**) Exp 3472 (Supplementary Fig. S40), (**d**) Exp 3473-Zone D (Supplementary Fig. S42), (**e**) Exp 3475-Zone B (Supplementary Fig. S45), (**f**) Exp 3474-Zone C (Supplementary Fig. S44), (**g**) Exp 3476-Zone G (Supplementary Fig. S48), (**h**) Exp 3477-Zone E (Supplementary Fig. S49).

**Figure 7:**
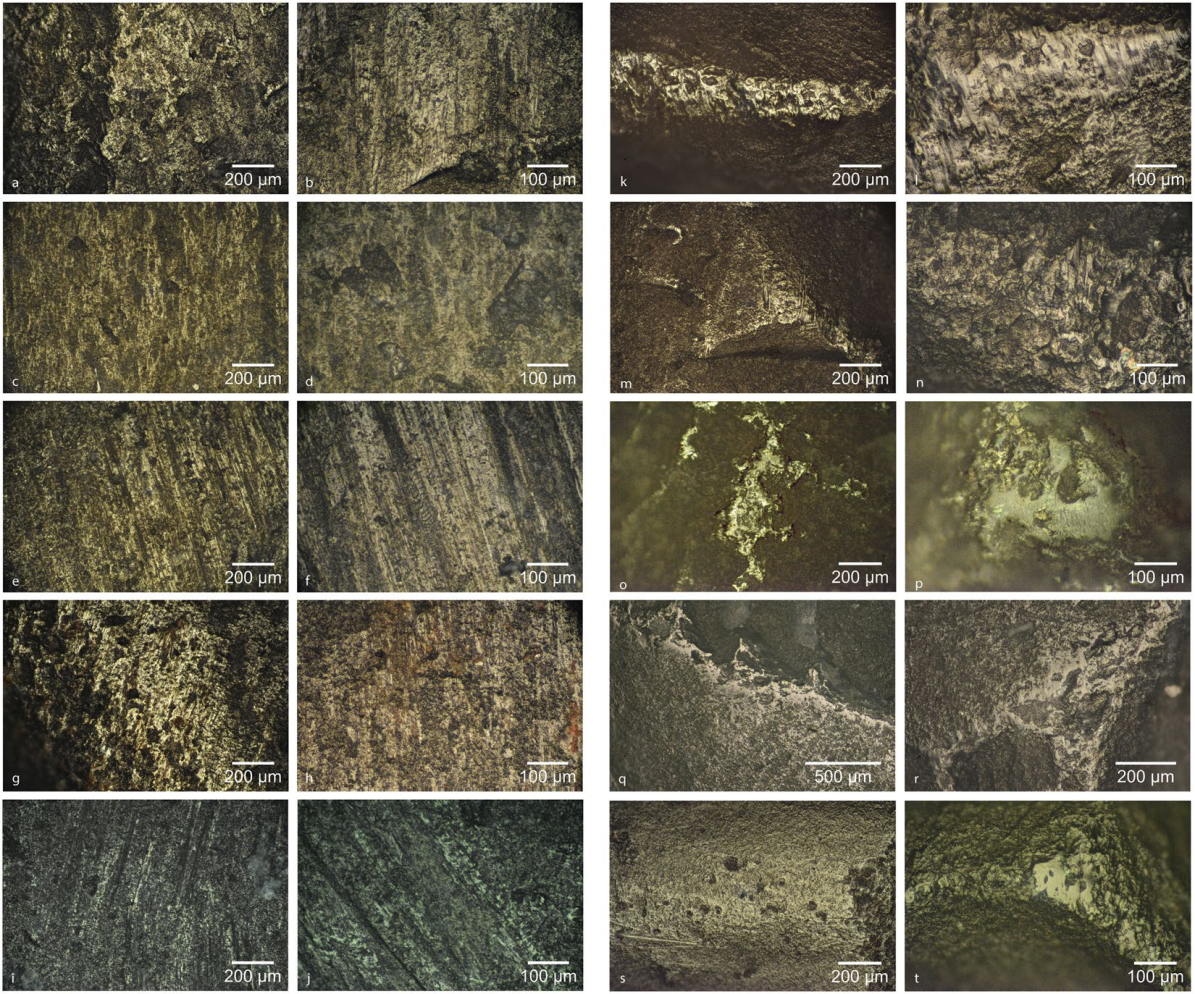
Images of experimental microwear traces of other mineral materials at high-magnification. See Supplementary Table S3 for more detailed descriptions of the experimental tools pictured here. (**a**) Flint, Exp 3473-Zone A (Supplementary Fig. S41), (**b**) Flint, Exp 3476-Zone F (Supplementary Fig. S48), (**c**) Quartz, Exp 3474-Zone B (Supplementary Fig. S43), (**d**) Quartz, Exp 3474-Zone B (Supplementary Fig. S43),(**e**) Sandstone, Exp 3474-ZoneA (Supplementary Fig. S43), (**f**) Sandstone, Exp 3474-Zone A (Supplementary Fig. S43), (**g**) Iron-cemented sandstone, Exp 3474-Zone C (Supplementary Fig. S44), (**h**) Iron-cemented sandstone, Exp 3474-Zone C (Supplementary Fig. S44), (**i**) Quartzite, Exp 3476-Zone C (Supplementary Fig. S47), (**j**) Quartzite, Exp 3476-Zone D (Supplementary Fig. S47), (**k**) Calcareous cortex of a flint nodule, Exp 3475-Zone D (Supplementary Fig. S46), (**l**) Calcareous cortex of a flint nodule, Exp 3475-Zone D (Supplementary Fig. S46), (**m**) Limestone, Exp 3475-Zone A (Supplementary Fig. S45), (**n**) Limestone, Exp 3475-Zone A (Supplementary Fig. S45), (**o**) Hematite, Exp 3478-Zone A (Supplementary Fig. S51), (**p**) Hematite, Exp 3478-Zone B (Supplementary Fig. S51), (**q**) Goethite, Exp 3479 (Supplementary Fig. S52), (**r**) Goethite, Exp 3479 (Supplementary Fig. S52), (**s**) Manganese dioxide, Exp 3480 (Supplementary Fig. S53), (**t**) Manganese dioxide, Exp 3481 (Supplementary Fig. S54).

The use traces imparted onto a stone tool appear as one or more of the following types of surface damage, depending on the material being worked and the duration of the task: polish, linear traces (i.e. striations, scratches, grooves), rounding, fractures, surface/edge removals and crushing (i.e. abundant overlapping fractures causing extensive surface removal) (Figs 5–7). Generally speaking, the mineral use-wear traces observed on our experimental and archaeological pieces can be broken down into four main categories: retouching/flintknapping, non-directional percussive, directional percussive and directional frictive traces. Retouching/knapping traces consist of single or clustered linear gouges in the surface of the flint, sometimes overlying (semi-)circular percussion marks^46,51^, often oriented in a similar direction allowing for the determination of the direction of motion (Fig. 5f,g, Supplementary Figs S47, 48). The non-directional percussive traces seem to indicate some sort of pounding activity where the direction of force is roughly perpendicular to the surface of the tool, creating isolated or grouped circular percussion marks (i.e. incipient Hertzian cones) on flatter surfaces without associated linear gouges, or extensive crushing of salient points and ridges (Fig. 5c,d, Supplementary Figs S38, S40). When the battering is excessive, it may be difficult to distinguish between pounding and flintknapping activities due to fracturing and surface loss. The directional percussive traces are also comprised of single or clustered percussion marks, but instead of being fully circular, they are instead C-shaped, indicating a more oblique blow (Fig. 5a,b, Supplementary Figs S39, S44). Experiments have shown that the Cs open towards the direction the percussor is travelling, and thus can indicate the relative motion of the two elements. Finally, the directional frictive traces created during activities involving grinding, forceful rubbing or, at times, oblique percussion, manifest as polish and/or striations, the latter often indicating the relative directionality of the interacting elements (Figs 6,7).

#### Fire making traces

The traces produced by pyrite on flint during fire making generally conforms to a combination of directional percussive and frictive traces. At the macroscopic level, this activity can produce clusters of unidirectional C-shaped percussion marks, rounding of flake scar ridges and some crushing (Fig. 5a–d). At the microscopic level, these traces generally occur as zone of matte, rough polish containing densely packed clusters of parallel to sub-parallel striations and scratches (Fig. 6). While usually a percussive task, percussion marks are not always present or readily noticeable. This could be due to a number of reasons, including the nature of raw material (percussion marks are sometimes more difficult to observe in coarse-grained stone, e.g. Supplementary Figs S34, S49), the force of the blow (often dependent on the size of the pyrite fragment, with larger fragments yielding larger incipient cones), and/or the surface morphology of the pyrite fragment (salient/convex surfaces are more likely to produce percussion marks than a flatter surface due to the greater concentration of force). Therefore, it is possible to produce what appear to be purely frictive traces while employing oblique percussion. Moreover, it is also possible to create sparks using a purely frictive, forceful rubbing gesture (e.g. Exp 3475-Zone B; see Supplementary Table 3 and Supplementary Fig. S45), though this method was not as effective at producing sparks/fire as using oblique percussion.

While C-shaped percussion marks were common, other macroscopic traces observed in our experiments include crushing and/or heavy rounding of edges, flake scar ridges or other salient surfaces (Fig. 5, Supplementary Figs S38, S40). Microtraces include densely packed clusters of (sub)parallel striations within discrete zones of flat, matte polish, as well as microscopic manifestations of the crushing, rounding, and surface removals mentioned before. Often times these traces are associated with small pits (described also as ‘micro-potlids’^45^ and ‘craters’ or ‘micro-craters’^49,51^). Johansen and Stapert^45^ attribute these to friction heat, much like potlids formed when flint is exposed to fire, but based on our experiments, they may be at times more related to a fragment or salient portion of the pyrite plucking out portions of the flint surface as it carves out a striation, as indicated by the linearity of some of these pits (e.g. Fig. 6, Supplementary Fig. S46), small pyrite fragments tumbling between the two surfaces, or they may sometimes simply be an artefact of the surface topography of the flint.

#### Non-fire making traces

The experimental traces created by grinding iron oxide (hematite, goethite) and manganese dioxide minerals across flake scar ridges to produce powder^52^ produces a bright, flat polish lacks pronounced striations (Fig. 7o–t, Supplementary Figs S51–54). Linear groupings of closely spaced C-shaped incipient cones (also referred to as a frictive track or ‘chattersleek’) were common within the goethite and hematite traces (Supplementary Figs S51, 52). These traces differ substantially from those observed on the archaeological bifaces^41^, with the degree of wear to the ridges also being much too minimal, and can likely be discounted as candidates for explaining the unidentified mineral use traces. Moreover, iron oxide residues (e.g. Fig. 5h, Supplementary Figs S50, 51) were particularly difficult to remove from the experimental pieces during cleaning, even when subjected to harsh acids, suggesting these residues, if ever present on archaeological pieces, would be more likely to preserve than pyrite residues.

Siliceous rocks (flint, quartzite, quartz, sandstone) tend to exhibit a streaky polish, not as flat as pyrite and sometimes having a reticulated appearance (i.e. features perpendicular to the motion direction, somewhat similar to a frictive track) (Fig. 7a–j). Striations are variable in expression, both in number and nature. Quartz striations are generally wider and poorly expressed (Fig. 7c,d, Supplementary Figs S43, S49). Sandstone and quartzite often create packed clusters of shallow striations with occasional wider, deeper, U-shaped cuts into the surface of the flint, likely corresponding to salient individual sand grains (Fig. 7e–j; Supplementary Figs S43, 44, S47, S50). Flint polish appears more domed with only occasional striations with widths and depths intermediate between sandstone/quartzite and pyrite (Fig. 7a,b; Supplementary Figs S47, 48). The surface of the flint often has a ‘cloudy’ appearance due to resistant, additive siliceous residues. Of these, iron-cemented sandstone was the most apt to produce polish and striations somewhat similar to the mystery traces in question. Linear gouge marks generally associated with retouching and flintknapping (Fig. 5f,g, Supplementary Figs S47, 48) are not usually produced during other percussive activities (e.g. fire making), and non-directional circular percussion marks without associated (uni)directional frictive traces are more likely resulting from pounding activities. Grinding, rubbing or abrading activities with these materials result in directional frictive traces, but rarely produce percussion marks. These, if present, are relatively few in number and tend to be found at points where there is a sudden change in relief, where the object moving across the surface of the biface either encounters a step fracture causing the abrading piece to suddenly drop onto a lower surface of the biface, as was the case with Exp 3473-Zone B (Fig. 5e, Supplementary Fig. S41) used to back a flake, or if it encounters a more raised surface like a high flake scar ridge, as seen on Exp 3473-Zone A (Supplementary Fig. S41) used to abrade the edge of another flint biface.

Calcareous stone was found to be neither hard nor abrasive enough to impart the heavy ridge rounding observed on the archaeological pieces without considerable effort. The resultant polish is domed, with wider more shallow (undulating) striations (Fig. 7k–n, Supplementary Figs S45, 46). Sand grain inclusions would occasionally create deeper isolated striations more akin to those created by sandstone (Fig. 7m, Supplementary Fig. S45).

### Archaeological results

All the artefacts examined for this study are listed in Supplementary Table 1, which also indicates their interpreted uses and associated figure numbers. Based on the comparisons with experimental material, both the character and distribution of the use traces imparted onto experimental bifaces used to make fire compare well with those encountered on a number of the archaeological specimens: 26 surfaces on 20 bifaces appear to exhibit traces that indicate either probable or possible use of the tool as a strike-a-light (e.g. Figs 1, 2). Ten surfaces on eight of the archaeological pieces exhibit what we consider retouching/flintknapping marks that are not associated with comparable zones of directional frictive traces (e.g. Fig. 1c; Supplementary Figs S7, S27), while eight other surfaces have what appear to be overlapping zones of retouching/flintknapping and directional percussive/frictive traces that are likely unrelated to one another, reinforcing the multi-use nature of these tools (e.g. Supplementary Figs S1, S20). When present, other non–mineral microwear traces (as reported in^38,41^) are indicated in the Supplementary Information figures and listed in Supplementary Table S1.

#### Orientation and distribution of probable fire making traces

As was the case in our experiments, adjustments to how a biface is held can result in different spatial distributions of the traces. However, despite the variability observed in the distribution of the traces on the archaeological pieces, when the location of the traces is considered together with the orientation of the striations and percussion marks, the inferred motion is likely to be indicative of the orientation of the biface and finger placement during use, and may even be indicative of the handedness of the user. Moreover, the size of the biface relative to the corresponding piece of pyrite used to make fire likely dictated which was used as the active element. Larger biface specimens were likely held passively while being struck with a smaller piece of pyrite, in some cases with the proximal (prehensile) end of the biface positioned downward, perhaps resting on the ground or some other stable substrate, with the tinder placed at its base. This could, for example, explain the proximal crushing observed on biface BvD 12582 (Supplementary Fig. S31). The location of the traces on other archaeological bifaces (e.g. CPN 99 W9, Supplementary Fig. S2; CPN E15-324, Supplementary Fig. S13) suggest that they may have been held with the distal end pointed downward, the tip of the biface either resting on the substrate, or more likely, held above the tinder material (See Supplementary Video S1). On some of the smaller bifaces (e.g. CPN E13-718, Supplementary Fig. S8), it is possible that they were struck against a passively held block of pyrite (Compare with Exp 3472, Supplementary Fig. S40, Supplementary Table S3). This variability in sizes, plus the variable nature and orientation of the flake scars on each biface, as well as the fact that most of the bifaces were further reduced and reshaped after use, and of course not forgetting personal preferences, can all account for the different locations of use zones between the archaeological pieces. Moreover, all of these methods were found to be effective at producing showers of sparks.

On the archaeological bifaces, use traces are consistently oriented parallel either to the longitudinal axis or to one of the lateral edges of the tool, and often perpendicularly cut across flake scars produced while shaping the biface. This is likely due to the flake scar ridges acting as a rough, abrasive surface that aids in creating sparks when struck with pyrite. However, experiments of longer duration (e.g. Exp 3470, Fig. S38, Supplementary Table 3) have shown that these surfaces become worn and less effective at producing sparks over time, which can have a limiting effect on the amount of time any one surface is used. Some bifaces exhibit particularly heavy mineral use-wear on both sides of the tool (e.g. Fonseigner 77, A2 Base Foyer, Niveau B, Supplementary Fig. S24), or on one side with variable directionality (e.g. BdV 2692, Fig. 2; Meyrals, Fig. 2; CPN F15-55, Supplementary Fig. S20; CPN F15-397, Supplementary Fig. S21). This phenomenon could indicate that the tools were used for more than one fire-making event, or that difficult conditions for making a fire (e.g. inclement weather, poor quality or slightly damp tinder) required a longer period of use that necessitated using a fresh surface after the utilized surface became too worn and less effective at producing sparks. However, the act of reshaping a biface through flintknapping effectively rejuvenates the surface of the biface, though in the case of the archaeological bifaces, it is likely that this would have been an added (though largely unintended) benefit of normal edge resharpening practices geared towards obtaining fresh cutting edges for other tasks like butchery. It is therefore interesting to note that some of the most well-developed directional percussive and frictive mineral use traces occur on bifacial thinning flakes (e.g. CPN E14-243, Fig. 3; CPN E19-318, Fig. 3; F15-397, Supplementary Fig. S21).

#### Bifacial thinning flakes

Included in our analysis were ten bifacial thinning flakes from CPN exhibiting mineral use traces. Of these, eight possess probable or possible strike-a-light microwear (Fig. 3; Supplementary Figs S4,S10–15, S21), one appears indicative of use for flintknapping/retouching (Supplementary Fig. S6) and another appears to have been used for some other unidentified percussive task (Supplementary Fig. S17). Four other bifacial thinning flakes with mineral use traces are known from CPN that were not included in our analyses (see Supplementary Table 2). Together with the biface evidence, these additional strike-a-light use zones make a total of 34 surfaces out of 49 analysed possessing these traces. That microtraces attributable to pyrite are observed on bifacial thinning flakes has two major implications, which—assuming these traces do indeed correspond to fire making—are consistent with the expedient strike-a-light model: 1) microwear evidence of a biface being used to make fire can potentially be lost as the tool is subsequently resharpened during its use life^34,35^; however, 2) identifying strike-a-light microtraces on resharpening or bifacial thinning flakes provide evidence that the inhabitants of a site were making fire using bifaces, either on- or off-site, even if the tools themselves were ultimately taken elsewhere.

## Discussion

The long use-lives of bifaces facilitate the recording of multiple isolated use events (e.g. for fire production, flintknapping, etc.) on their surfaces that are perhaps more visible and easier to identify than on expediently used components of Neandertal stone toolkits. Our observations suggest that curated tools produced by earlier Neandertals (e.g. Quina Mousterian scrapers, Micoquian and Keilmesser bifacial tools) and much older hominins (i.e. Acheulean handaxes) throughout Eurasia and Africa have the potential to yield comparable fire making traces that could provide valuable insight into when and where in our deep past fire production became a fixed part of the hominin technological repertoire. Indeed, traces corresponding to repeated forceful contact with mineral materials have been observed on bifacial tools as early as the Acheulean (see Table 1 in^41^, and the sources therein). These often appear at the thickest or most prominent portions of the flat surfaces as ‘battering marks’ (percussion marks, linear surface gouging, heavy localized crushing) that appear to be associated with flintknapping and/or various heavy pounding activities. Despite apparently lacking the characterised striated mineral microwear traces and oriented C-shaped percussion marks observed on our pieces attributed to fire making, these Lower Palaeolithic tools demonstrate the great time depth involved in using the flat sides of bifaces for percussive tasks. The use of flaked surfaces to process mineral materials (i.e. for grinding pigments into powder) has been observed on large curated unifacially-flaked scrapers attributed to the Quina Mousterian^52^. However, mineral use traces possibly corresponding to fire making like those observed on the bifaces discussed in this study have not currently been observed on these older artefacts (preliminary research conducted by Sorensen). Finding fire making traces on such tools produced during colder climatic periods (i.e. during MIS 4) would be particularly important, considering it has been postulated that an apparent reduction in fire use signals during these periods may indicate Neandertals were unable to make fire^24,25^ (however, see^28^). However, bifaces from numerous other late MP sites, mostly in France (see Fig. 4 and Supplementary Table S2, and references listed therein), but also in the Netherlands^53^, possess evidence of Neandertals utilizing the flat faces for mineral-related tasks. These include as percussors/retouchers for flintknapping or other pounding activities, but some of the observed traces look very similar to our inferred fire making traces, especially at Bas-du-Mont des Bruyères (Saint-Amand-les-Eaux, Nord) in northern France^37,54^, La Rochette (Dordogne)^41^ and at La Quina in Charente-Maritime (E. Claud, unpublished observation).

While we cannot know the motivations behind many activities performed by Neandertals using stone tools, the gestures required to produce the traces present on the late MP bifaces appear to fit well within a fire making framework, not only because the effectiveness of the method, but also that using the flat side of the biface to make fire leaves the edges sharp and undamaged. Moreover, the method makes it easier to use very small or heavily reduced pieces of pyrite by negating some of the problems of force and accuracy that come with using such small fragments.

The utility of the biface fire making method was recognized by Neandertals, as suggested by the number of late MP bifaces exhibiting pyrite-like mineral microwear traces. This promotes the idea that using a biface to make fire was not an expedient task, but was instead a known use for these tools, just as the purpose of the can opener on a Swiss Army Knife is clear despite this not being the primary function of the tool. This helps to complete our knowledge of the functional status of late MP bifaces. It was already known that they were curated, highly mobile elements of the Neandertal toolkit^32,34,35^ that were manufactured on one site and transported to the next, where the sharp edges were used primarily for butchering animals, but also for other activities like working wood/vegetal materials, bone and hide^41,55^. They could also be used to obtain some flakes that were used for still other tasks, these bifacial thinning flakes perhaps being the only evidence remaining that a biface was used on a site^56^. And now we know that Neandertals used the surfaces of these tools for fire making, as well as for flintknapping, retouching and other percussive activities. Together, these complimentary uses of the bifaces support the very logical conclusion that these tools were taken as personal gear during displacements.

Ample evidence for Neandertal fire use during the late MP provides a degree of support for our suspicion that late Mousterian bifaces occasionally functioned as fire making tools (for comprehensive lists of MP sites exhibiting evidence of fire use, see^27,28^). Nearly all of the bifaces and bifacial thinning flakes with mineral use traces from CPN were recovered from a layer (SW-US07) with relatively high proportions of burned bone (~8%)^41,57,58^. The MTA layers at Pech I have multiple evidences for fire use, including hearth features, combusted/charred bone and heated lithics^42,59–61^. Heated lithics and possible structured hearths are noted at Fonseigner^32,62^, while a minor amount of heated lithics were recovered at Le Prissé^39^. Moreover, the pyrotechnic capabilities of manganese dioxide as a possible tinder enhancer has recently been demonstrated^29^, where the ignition temperature of the tinder is lowered by around 100 °C. Manganese dioxide is a blackish mineral common to Mousterian contexts^63^, including hundreds of fragments recovered from the MTA layers at Pech de l’Azé I^43^. The experiments performed for this study have confirmed that the addition of powdered manganese dioxide to tinder indeed improves the efficacy of the material by making it more readily accepting of sparks produced using the biface-and-pyrite fire making method (see Supplementary Video S1).

Nevertheless, we recognise that the associations between bifaces with probable fire making traces and evidence of fire use could be considered circumstantial, since not every site bearing evidence for the manufacture and use of bifaces possesses strong evidence for fire use^64^. If Neandertals were indeed capable of producing fire at will, it does not necessarily mean they would have made it at every site they visited^28^. And given the long use-lives of MTA bifaces, it is entirely possible for a biface to have been used to make fire at one site and then discarded at another where fire was not used. Moreover, considering the great variability with which fire residues and fire proxies (i.e. fire-affected lithic artefacts and faunal remains) are produced and preserved, not every site where fire was used will retain strong evidence of its presence (see^65^). Finally, and perhaps counterintuitively, it could be possible that possessing fire making technology could at times *reduce* archaeological fire signals^28^. Having the ability to make fire as needed would negate the need to constantly maintain fires captured from natural sources (e.g. wildfires) for long periods of time so as a preservation measure. This would be especially important during colder periods when woody fuel was less abundant in the environment and fuel economisation was paramount. This potentially has major implications for how archaeologists interpret anthropogenic fire signals during the MP. Moreover, our demonstration here that Neandertals were able to produce fire at will during the MTA implies that they were also capable of making fire during other periods when different technological strategies for flake and stone tool manufacture were being employed. The potential variability in how fire making tools manifest within these systems could be one of the major reasons why so few have been identified to date^14^.

## Conclusion

Numerous Neandertal bifacial tools and bifacial thinning flakes from late MP contexts in France, especially those attributed to the Mousterian of Acheulean Tradition, possess macroscopic and microscopic use traces suggesting repeated contact with a mineral material. Some of these traces result from flintknapping and retouching activities that create linear gouges in the surface of the flint, while others can be attributed to various pounding activities. Other traces, more friction-like and often times accompanied by clusters of C-shaped percussion marks—both indicating unidirectional motion—are more quizzical, the process(es) by which these traces were produced remaining largely unexplained until this study. After careful comparison with different types of mineral use traces produced on experimental bifaces, we have concluded that those resulting from repeated forceful contact with pyrite for the express purpose of producing sparks for fire making conform best to the unidentified archaeological traces. Moreover, the resultant fire making traces on the experimental bifaces are distributed in a manner consistent with those on the archaeological pieces. Together, these points support the hypothesis that some of these bifaces were occasionally used as fire making tools. While no associated pyritic residues were observed that could provide additional support for this interpretation, this is probably due to the corrosive, and therefore ephemeral, nature of this mineral. Nevertheless, it is still possible that optically invisible pyritic micro-residues could remain on some artefacts, and we are currently looking into the applicability of various chemical analytical methods like SEM-EDAX, μ-XRF and RAMAN spectroscopy as prospection tools for identifying these residues, if present (c.f.^6,12,21^). Ultimately, the prevalence of probable ‘strike-a-light’ use traces among late Mousterian biface-bearing lithic assemblages suggests for the first time that the use of bifaces for fire production may have been an important regional technocultural phenomenon at the end of the MP in France. This has significant implications for our understanding of Neandertal cognitive abilities, including increased planning depth and the use of multicomponent tools, and further highlights the intimate relationship these peoples had with fire.

## Methods

### Archaeological corpus

We know of at least 59 late MP bifaces (and 14 bifacial thinning flakes) from 17 sites in France and one in the Netherlands that exhibit percussive and/or frictive traces related to undefined activities involving some sort of ‘mineral’ material(s)^32,34,37,38,40,41,43,46,51,55^ (Supplementary Tables S1 and S2), with fire making perhaps being among these tasks. While some bifaces edges exhibit heavy crushing and edge removals consistent with percussive contact with hard mineral materials^41^, the zones of interest to this study are those located not on the edge of the tools, but instead on their flat or convex ‘faces’. Of these bifaces, 27 examples from seven sites in SW France were examined for this study (Fig. 4; Supplementary Table S1): Layers US08-06 at CPN^38,41^, and Layer 4 at Pech I^43^, the MP level at BdV^40,44^, Archaeological Levels B and D-supérieur at Fonseigner^32,41^, Layer 4 at Le Prissé^39,40^, and surface scatters at Sarlat and Meyrals (unpublished findspots; A. Turq, pers. comm.). Moreover, nine bifacial thinning flakes and one indeterminate flake from CPN exhibiting mineral use traces were also analysed, making a total of 49 utilized surfaces analysed. Detailed descriptions of the tools and their associated sites can be found in the original publications listed in Supplementary Table S1.

### Experimental methods

Archaeologists use functional experiments to help determine how archaeological tools were used by attempting to replicate macroscopic and microscopic use traces observed on these tools^66–70^. The findings of Claud^37,38,41^ indicate that the agent (or agents) responsible for these traces is a hard mineral material, though despite the implementation of several comparative experiments (e.g. use as a retoucher, percussor or abrader on various stone types, grinding mineral pigments, etc.), the precise nature of these friction traces remains largely unknown. However, the first author observed that some of these traces resembled those produced experimentally by percussive fire making using pyrite^14^, thus providing the impetus for this study.

Careful study of the location and character of the archaeological use traces has been undertaken to guide our experiments. All experiments were therefore performed using the flat/convex faces of the bifaces (Supplementary Table S3). Moreover, given the oriented nature of the percussion marks and striations, all of the experiments performed utilised gestures employing unidirectional or bidirectional motions (as opposed to non-directional percussive tasks, e.g. using the biface as an anvil surface, that have an angle of incidence close to 90°). For the fire making experiments, pyrite fragments with different crystal habits, including a nodule fragment with a fine-grained fibroradial crystal habit, a granular aggregate comprised of fine- to medium-grained crystals, and a large euhedral cubic crystal, were used to test for possible variability. In addition, prior to experimentation, different gripping systems and several methods of application of force were practiced in order to test for spark production efficiency and comfort of use (e.g. Supplementary Video S1). These helped us to set up the experimental protocol. Experimental bifaces were struck using tangential blows or forcibly rubbed between 1 and 30 minutes, depending on the experiment, and regularly produced sparks that were captured by tinder material (primarily tinder fungus, *Fomes fomentarius*, mixed with manganese dioxide power^29^).

The other mineral experiments utilized rock and mineral specimens common to MP archaeological sites, including flint, quartzite, quartz, limestone, sandstone, iron oxide minerals (hematite, goethite) and manganese dioxide. Most of these were rubbed against the bifaces, while some of the experiments involving flint were percussed to simulate flintknapping/retouching (Supplementary Table S3). These experiments ranged from 1 to 10 minutes.

Prior to microscopic examination, all experimental bifaces were washed using soap and water. Bifaces with persistent siliceous residues were then placed in a sonic bath at 60 °C for 90 minutes for further cleaning, as were bifaces with carbonate residue after brief immersion (1–2 minutes) in 10% hydrochloric acid. Experiments using pyrite and iron oxide minerals required alternative cleaning protocols to remove stubborn residues that can often obscure microscopic traces. Bifaces with pyrite residues were soaked in a super-saturated sodium bicarbonate (NaHCO3, aka baking soda) solution, either for three days at room temperature, or placed inside a sonic bath at 60 °C for 90 minutes. Bifaces with iron oxide residues were soaked in 10% oxalic acid at 60 °C for 90 minutes in a sonic bath. Bifaces soaked in these solutions were then allowed to cool, rinsed, and then returned to the sonic bath in clean water for 90 additional minutes to remove any remaining chemicals.

### Analytical methods

Both experimental and archaeological bifaces were examined at the mesoscale using a binocular microscope (low-magnification, 10–60x) and at the microscale using a metallographic reflected light microscope (high-magnification, 50x, 100x and 200x). Low-magnification analysis allows for the identification and characterisation of utilized zones based on the presence of macroscopically observable damage to the surface of the tool and/or associated residues that often provide insight into the type of mineral that was worked (hard vs. soft, metallic/submetallic, etc.), as well as the motion employed^46,71–78^, while high-magnification analysis provides greater insight into the precise nature of the observed traces^66–68^. Zones exhibiting mineral microwear traces are delineated in the figures. No apparent associated traces were observed outside these zones.

Complementing the experiments performed specifically for this study, extant experimental reference collections (i.e. the Leiden Material Culture Studies Laboratory Experimental Reference Collection) were also consulted to help evaluate use traces evident on the archaeological material^14,41^.

### Data Availability

All data generated or analysed during this study are included here and in the Supplementary Information file.

## Electronic supplementary material

Supplementary Information

Supplementary Video S1
