# Supplementary material for: Neandertal fire-making technology inferred from microwear analysis
Source: Sci Rep. 2018 Jul 19;8:10065. doi: 10.1038/s41598-018-28342-9 (PMC6053370; doi:10.1038/s41598-018-28342-9)
Supplement: Supplementary file 1 — Supplementary Information [file 41598_2018_28342_MOESM1_ESM.pdf]

Supplementary Information

Neandertal fire-making technology inferred from microwear analysis

A.C. Sorensen[1]*, E. Claud[2,3], M. Soressi[1]

[1] Faculty of Archaeology, Leiden University, Leiden, The Netherlands
[2] INRAP, GSO, Bègles, France
[3] UMR 5199 PACEA, Université de Bordeaux, Pessac, France

*Corresponding author: a.c.sorensen@arch.leidenuniv.nl

Supplementary Video

Supplementary Video S1. Video demonstrating the biface and pyrite method of fire making. Video credit: Weiya Li, Leiden University.

Supplementary Tables

Supplementary Table S1. Table of archaeological bifaces subjected to microwear analysis for this study and their interpretations.

# in Fig. 4	Site	Artefact #	Provenience	Type	Side	General location of traces	Percussion marks	Location (in relation to microwear traces)	Orientation	Linear gouges	Orientation	Crushing	Striations	Orientation	Possible contact material	Inferred function	Notes	Other observed microwear traces and locations	Relevant literature and page numbers	Fig. #
**1**	Chez-Pinaud/ Jonzac (Charente-Maritime)	CPN 03 D18 s4 7s 5,55-5,60		Biface	A	Mesial-central	Yes (n=~20)	Proximal-Mesial	Most open distally	Yes	Random, clustered to the right of other mineral traces	Yes, along flake scar ridge in percussion zone	Yes		Pyrite, flint	Strike-a-light? and retoucher	Central zone of percussion with C-shaped marks opening distally likely related to mineral traces, while right percussive zone contains linear gouges that likely correspond to a brief episode of use as a retoucher.	Cutting hide (left edge, mesio-distal part) and cutting meat or hide (butchery) (right edge, mesio-distal part)	Claud 2008 (p. 318, 319)[1], 2012[2]	S1
**1**	Chez-Pinaud/ Jonzac (Charente-Maritime)	CPN 99 W9	Level 8, locus W	Biface	A	Mesial-distal	Very few	Proximal					Abundant	Parallel long axis	Pyrite?	Strike-a-light?	Noticeable rounding of flake scar ridges. Traces cover entire distal half of the tool.	None observed	Claud 2008[1], 2012[2]	S2
**1**	Chez-Pinaud/ Jonzac (Charente-Maritime)	CPN 99 W9	Level 8, locus W	Biface	B	Mesial-central	Yes	N/A	Random	Yes			Few		Flint	Retoucher		None observed	Claud 2008[1], 2012[2]	S2
**1**	Chez-Pinaud/ Jonzac (Charente-Maritime)	CPN D16-316	SW-US06.1	Biface	A	Proximal-Central	Yes (n=>12)	Throughout	Generally open proximally				Yes, minor	Subparallel to long axis, parallel to left lateral edge	Traces not very diagnostic	Uncertain, possibly a strike-a-light?	Very brief use	None observed	Claud 2008[1], 2012[2]	S3
**1**	Chez-Pinaud/ Jonzac (Charente-Maritime)	CPN D16-486	SW-US06.1	Bifacial thinning flake	Dorsal	Distal tip							Yes	Possibly two sets of striations sub-parallel to one another, both oriented roughly perpendicular to long axis	Pyrite	Strike-a-light		Cutting soft material (left edge, mesio-proximal part)	Claud 2008 (p. 421, h)[1], 2012[2]	S4
**1**	Chez-Pinaud/ Jonzac (Charente-Maritime)	CPN D18-190	SW-US07.3	Biface	A	Proximal-central	Yes		Some open proximally	A few		Yes			Unclear	Uncertain percussive task	Percussive traces similar to CPN E13-718, but no associated microwear traces observed.	None observed	Claud 2008[1], 2012[2]	S5
**1**	Chez-Pinaud/ Jonzac (Charente-Maritime)	CPN D18-200	SW-US07.3	Bifacial thinning flake	Dorsal	Mesial-Central	Yes			Yes		Yes			Flint	Flintknapping, retoucher	No associated mineral traces	None observed	Claud 2008[1], 2012[2]	S6
**1**	Chez-Pinaud/ Jonzac (Charente-Maritime)	CPN D19-823	SW-US07.3	Biface	A	Left-Central				Yes (n=7)	Generally perpendicular to long axis				Flint	Retoucher		None observed	Claud 2008[1], 2012[2]	S7
**1**	Chez-Pinaud/ Jonzac (Charente-Maritime)	CPN E13-718	SW-US07.4	Biface	B	Proximal-mesial-central	Yes, many	Throughout	Ambiguous	Few		Yes, extensive at proximal end	Yes	Subparallel to long axis	Pyrite, flint?	Strike-a-light, perhaps brief use as retoucher	Perhaps at least two gestures represented: pyrite used as active towards proximal end, and proximal end of biface used as active (distal end up)	None observed	Claud 2008[1], 2012[2]	S8
**1**	Chez-Pinaud/ Jonzac (Charente-Maritime)	CPN E13-748	SW-US07.4	Biface	A	Mesial-central	Yes (n=>50), C-shaped	Proxial-Mesial	Open distally				Yes	Parallel long axis	Pyrite	Strike-a-light		Possible cutting/chopping medium hard material (right edge, mesial part) and percussion traces (wedge?) (proximal part)	Claud 2008 (p. 315-317)[1], 2012[2]	1, S9

# in Fig. 4	Site	Artefact #	Provenience	Type	Side	General location of traces	Percussion marks	Location (in relation to microwear traces)	Orientation	Linear gouges	Orientation	Crushing	Striations	Orientation	Possible contact material	Inferred function	Notes	Other observed microwear traces and locations	Relevant literature and page numbers	Fig. #
**1**	Chez-Pinaud/ Jonzac (Charente-Maritime)	CPN E13-748	SW-US07.4	Biface	B	Mesial-central	Yes		Random	Yes	Perpendicular long axis		Few		Flint	Retoucher		Possible cutting/chopping medium hard material (left edge, mesial part) and percussion traces (wedge?) (proximal part)	Claud 2008 (p. 315-317)[1], 2012[2]	1, S9
**1**	Chez-Pinaud/ Jonzac (Charente-Maritime)	CPN E14-161	SW-US07	Bifacial thinning flake	Dorsal	Distal-Left							Yes	Oblique to long axis	Pyrite?	Strike-a-light?	Traces moderately well-developed at best	Scraping hard material (right edge, distal part) and indeterminated movement on soft material (left edge)	Claud 2008 (p. 422, f)[1], 2012[2]	S10
**1**	Chez-Pinaud/ Jonzac (Charente-Maritime)	CPN E14-243	SW-US07	Bifacial thinning flake	Dorsal	Distal-Mesial-Left							Yes	Oblique to long axis	Pyrite	Strike-a-light	Very well-developed traces, strong flake scar ridge rounding	Cutting soft material (left edge, proximal part)	Claud 2008 (p. 421, k)[1], 2012[2]	3, S11
**1**	Chez-Pinaud/ Jonzac (Charente-Maritime)	CPN E14-276	SW-US07	Bifacial thinning flake	Dorsal	Distal-Left							Yes	Oblique to long axis	Pyrite?	Strike-a-light?	Traces moderately well-developed at best in small localized area	Cutting meat (left edge)	Claud 2008 (p. 421, j)[1], 2012[2]	S12
**1**	Chez-Pinaud/ Jonzac (Charente-Maritime)	CPN E15-324	SW-US07	Biface, distal portion	A	Distal-Proximal-Central							Yes	Parallel long axis and right lateral edge	Pyrite	Strike-a-light	Zone of percussion, if originally present, was perhaps on lost proximal portion of the biface, or at distal tip and subsequently removed during resharpening(?)	Cutting meat (right edge) and scraping hard mineral material (left edge)	Claud 2008 (p. 343, 344)[1], 2012[2]	S13
**1**	Chez-Pinaud/ Jonzac (Charente-Maritime)	CPN E15-370	SW-US07	Bifacial thinning flake	Dorsal	Distal-Central							Yes	Oblique to long axis	Pyrite	Strike-a-light		None observed	Claud 2008[1], 2012[2]	S14
**1**	Chez-Pinaud/ Jonzac (Charente-Maritime)	CPN E16-550	SW-US07	Bifacial thinning flake/ convex side scraper	Dorsal	Proximal-Mesial-Central							Yes	Variable, roughly parallel to long axis	Pyrite	Strike-a-light	Possibly more than one use epises. Traces likely imparted prior to removal. Much of piece is heavily polished, possibly due to prehension and use as a scraper.	None observed	Claud 2008[1], 2012[2]	S15
**1**	Chez-Pinaud/ Jonzac (Charente-Maritime)	CPN E18-30	SW-US07.3	Biface	A	Mesial-left half	Yes (n=3)	Central, proximal side of flake scar ridges	Open distally				Yes	Two directions, parallel long axis and to right lateral edge	Pyrite	Strike-a-light	Possibly more than one use episodes. May have used a forceful rubbing gesture, which could account for few percussion marks; strong rounding of flake scar ridges	Cutting soft to medium hard material (butchery?) (left edge, mesio-distal part)	Claud 2008 (p. 334, 335)[1], 2012[2]	1, S16
**1**	Chez-Pinaud/ Jonzac (Charente-Maritime)	CPN E18-32	SW-US07.3	Bifacial thinning flake	Dorsal	Mesial-Left-Central	Yes (n=>20)		Many open towards right lateral edge						Unclear	Uncertain percussive task	Truncated percussion marks in right flake negative. No other mineral traces observed.	Cutting meat (left edge)	Claud 2008 (p. 421, g)[1], 2012[2]	S17
**1**	Chez-Pinaud/ Jonzac (Charente-Maritime)	CPN E19-318	SW-US07.3	Bifacial thinning flake	Dorsal	Distal-Mesial-Right	Yes (many, heavily clustered)	Throughout	Variable, though many appear to open distally			Yes	Yes	Oblique to long axis	Pyrite	Strike-a-light	Very well-developed traces	None observed	Claud 2008[1], 2012[2]	3, S18
**1**	Chez-Pinaud/ Jonzac (Charente-Maritime)	CPN E19-425	SW-US07.3	Biface	B	Central							Yes	Subparallel to long axis, parallel to left lateral edge	Pyrite	Strike-a-light		None observed	Claud 2008[1], 2012[2]	S19

# in Fig. 4	Site	Artefact #	Provenience	Type	Side	General location of traces	Percussion marks	Location (in relation to microwear traces)	Orientation	Linear gouges	Orientation	Crushing	Striations	Orientation	Possible contact material	Inferred function	Notes	Other observed microwear traces and locations	Relevant literature and page numbers	Fig. #
1	Chez-Pinaud/ Jonzac (Charente-Maritime)	CPN F15-55	SW-US07	Biface	A	Distal-Proximal-Central	Yes, numerous	Throughout, also a cluster of truncated Hertzian cones in flake negative at proximal end	Ambiguous, many open distally, but some proximally	Yes, a few	Perpendicular long axis		Yes	Two intersecting directions, one roughly parallel to long axis/ right lateral edge and another parallel to left lateral edge	Pyrite, flint	Strike-a-light and retoucher	Perhaps 2 to 3 use episodes, as suggested by variable directionality	None observed	Claud 2008[1], 2012[2]	S20
1	Chez-Pinaud/ Jonzac (Charente-Maritime)	CPN F15-397	SW-US07	Bifacial thinning flake	Dorsal	Distal-Mesial-Central							Yes	Oblique to long axis	Pyrite	Strike-a-light	Well-developed traces and flake scar ridge rounding	None observed	Claud 2008[1], 2012[2]	S21
1	Chez-Pinaud/ Jonzac (Charente-Maritime)	CPN F16-73	SW-US07	Biface	A	Mesial-central-left	Yes (n=~25)	Throughout	Most open distally				Yes	Subparallel to long axis	Unclear, possibly quartzite	Use as retoucher or for flinknapping	Quartzite appears to be less inclined to create linear gouges during knapping than flint; width/ depth/length and "smoothness" of striations more like those produced when knapping quartzite	Cutting soft to medium hard material (butchery?) (right edge, mesio-distal part)	Claud 2008 (p. 315)[1], 2012[2]	S22
1	Chez-Pinaud/ Jonzac (Charente-Maritime)	CPN F16-73	SW-US07	Biface	B	Mesial-central							Yes	Parallel long axis	Quartzite?	Use as etoucher or for flinknapping	Very brief use, minimal polish, similar to Side A but no percussion marks	Cutting soft to medium hard material (butchery?) (left edge, mesio-distal part)	Claud 2008 (p. 315)[1], 2012[2]	S22
2	Fonseigner (Dordogne)	77, 31, Fons 22, D sup 13	Couche 1, Niveau D supérieur 13	Biface	A	Mesial-Distal							Yes	Parallel to long axis	Pyrite	Strike-a-light	Possible prehension traces on proximal end	None observed (uncertain butchery traces on distal part and left edge, unpublished data)	Geneste 1985[3]; Claud 2008[1]	S23
2	Fonseigner (Dordogne)	77, 31, Fons 22, D sup 13	Couche 1, Niveau D supérieur 13	Biface	B	Right-Mesial-Distal							Yes	Two intersecting directions: parallel to the long axis throughout, as well as parallel to the left lateral edge in the more proximal portion	Pyrite	Strike-a-light	Possible prehension traces on proximal end. Noticeable amounts of reddish residue, mainly in "prehension" area.	None observed (uncertain butchery traces on distal part and right edge, unpublished data)	Geneste 1985[3]; Claud 2008[1]	S23
2	Fonseigner (Dordogne)	77, A2 Base Foyer, Niveau B	Couche 0, A2 Base Foyer, Niveau B	Biface	A	Central							Yes	Parallel to long axis	Pyrite	Strike-a-light	Well-developed flake scar ridge rounding. Possible prehension traces on proximal end.	No information available	Geneste 1985[3]; Claud 2008[1]	S24
2	Fonseigner (Dordogne)	77, A2 Base Foyer, Niveau B	Couche 0, A2 Base Foyer, Niveau B	Biface	B	Central-Mesial-Distal							Yes	Parallel to long axis	Pyrite	Strike-a-light	Well-developed flake scar ridge rounding. Possible prehension traces on proximal end.	No information available	Geneste 1985[3]; Claud 2008[1]	S24
3	Bout des Vergnes (Dordogne)	BdV 28	Middle Palaeolithic layer	Biface	A	Mesial-Proximal	Yes	Mostly in right lateral portion	Ambiguous, though generally open towards distal end				Yes	Two intersecting directions: parallel to the long axis, and perpendicular to the long axis	Unclear, possibly pyrite and siliceous material?	Strike-a-light and ?	At least two use episodes. Opposite face bears zone of heavy metallic traces/damage from contact with a metal tool (shovel?)	None observed	Ihuel (in prep.)[4]; Brenet et al. 2017[5]	S25
3	Bout des Vergnes (Dordogne)	BdV 781	Middle Palaeolithic layer	Biface	A	Right-Mesial Distal	Yes (few)	Throughout	Ambiguous				Yes	Subparallel to long axis and right lateral edge	Pyrite	Strike-a-light and ??	Central ridge has heavy metallic traces/damage from contact with a metal tool (shovel?)	None observed	Ihuel (in prep.)[4]; Brenet et al. 2017[5]	S26

# in Fig. 4	Site	Artefact #	Provenience	Type	Side	General location of traces	Percussion marks	Location (in relation to microwear traces)	Orientation	Linear gouges	Orientation	Crushing	Striations	Orientation	Possible contact material	Inferred function	Notes	Other observed microwear traces and locations	Relevant literature and page numbers	Fig. #
**3**	Bout des Vergnes (Dordogne)	BdV 781	Middle Palaeolithic layer	Biface	B	Central-Mesial	Yes (few)	Throughout	Ambiguous				Yes	Parallel to long axis	Pyrite	Strike-a-light		None observed	Ihuel (in prep.)[4]; Brenet et al. 2017[5]	S26
**3**	Bout des Vergnes (Dordogne)	BdV 1651	Middle Palaeolithic layer	Biface	A	Proximal-Central	Yes (n=~20)			Yes					Flint	Retoucher		None observed	Ihuel (in prep.)[4]; Brenet et al. 2017[5]	S27
**3**	Bout des Vergnes (Dordogne)	BdV 2629	Middle Palaeolithic layer	Biface	A	Proximal-Central	Yes (n=1)		Opening towards proximal end						Unclear	Percussor?		None observed	Ihuel (in prep.)[4]; Brenet et al. 2017[5]	S28
**3**	Bout des Vergnes (Dordogne)	BdV 2629	Middle Palaeolithic layer	Biface	B	Central	Yes (n=~20)		Ambiguous, more opening towards distal end	Yes (a few)	Perpendicular to long axis				Flint?	Retoucher		None observed	Ihuel (in prep.)[4]; Brenet et al. 2017[5]	S28
**3**	Bout des Vergnes (Dordogne)	BdV 2692	Middle Palaeolithic layer	Biface	A	Central	Yes	Throughout, but main concentration in center	Ambiguous			Yes	Yes	Subparallel to long axis and left lateral edge	Pyrite?	Strike-a-light?	Heavy pounding, could indicate larger pyrite nodule fragment and/or less oblique percussion	Cutting soft to medium hard material (butchery?) (right edge, distal part)	Ihuel (in prep.)[4]; Brenet et al. 2017[5]	2, S29
**3**	Bout des Vergnes (Dordogne)	BdV 2692	Middle Palaeolithic layer	Biface	B	Central-Mesial	Yes	Throughout	Ambiguous				Yes	Two intersecting directions: parallel to the left lateral edge and in the right distal section, parallel also to the right lateral edge	Pyrite	Strike-a-light		Cutting soft to medium hard material (butchery?) (left edge, distal part)	Ihuel (in prep.)[4]; Brenet et al. 2017[5]	2, S29
**3**	Bout des Vergnes (Dordogne)	BdV 7931	Middle Palaeolithic layer	Biface	A	Central-Mesial-Proximal				Yes (few)					Flint?	Retoucher	No associated mineral traces	None observed	Ihuel (in prep.)[4]; Brenet et al. 2017[5]	S30
**3**	Bout des Vergnes (Dordogne)	BdV 7931	Middle Palaeolithic layer	Biface	B	Center-Right-Mesial Distal	Yes (few)						Yes	Parallel to right lateral edge	Pyrite?	Strike-a-light?		None observed	Ihuel (in prep.)[4]; Brenet et al. 2017[5]	S30
**3**	Bout des Vergnes (Dordogne)	BdV 12582	Middle Palaeolithic layer	Biface	A	Central							Yes	Parallel to long axis	Pyrite?	Strike-a-light?	Rounded flake scar ridges. Striations present, but only very weak polish (taphonomic?)	Cutting hide (left edge, non-resharpened small part)	Ihuel (in prep.)[4]; Brenet et al. 2017[5]	S31
**3**	Bout des Vergnes (Dordogne)	BdV 12582	Middle Palaeolithic layer	Biface	B	Central-Proximal-Distal	Yes (few)						Yes	Parallel to long axis	Pyrite?	Strike-a-light?	Notable rounding of flake scar ridges. Flattened/dulled and polished along proximal edge, from resting biface on (hard) substrate (during fire making)?	Cutting hide (right edge, non-resharpened small part)	Ihuel (in prep.)[4]; Brenet et al. 2017[5]	S31

# in Fig. 4	Site	Artefact #	Provenience	Type	Side	General location of traces	Percussion marks	Location (in relation to microwear traces)	Orientation	Linear gouges	Orientation	Crushing	Striations	Orientation	Possible contact material	Inferred function	Notes	Other observed microwear traces and locations	Relevant literature and page numbers	Fig. #
**4**	Meyrals (Dordogne)		Surface find	Biface	A	Mesial-Proximal	Yes	Central	Somewhat ambiguous, but generally open towards proximal end			Some	Yes	Towards distal tip and parallel to right lateral edge	Pyrite	Strike-a-light	Bi-directionality of the striations could indicate two episodes of use. Numerous truncated percussion marks in flake negatives around central zone of percussion	No information available	Unpublished surface find by Mr. Lajoinie; A. Turq, pers. comm	2, S32
**5**	Sarlat (Dordogne)		Surface find	Biface	A	Central-Mesial-Proximal	Yes	Throughout, but mostly in right-proximal portion	Generally towards distal tip or parallel to right lateral edge			Very minor	Yes	Two directions, parallel to right lateral edge and towards dital tip	Somewhat unclear, possibly multiple (pyrite and flint?)	Strike-a-light, abrader, percussor?	Streaky whitish patination (additive residue?) could indicate abrasion with a siliceous material.	No information available	Unpublished surface find by Mr. Bigotto; A. Turq, pers. comm	S33
**6**	Pech de l'Azé I (Dordogne)	PAI F12-404	Layer 4	Biface	A	Central-Proximal-Distal	Yes	Proximal end	Ambiguous	Yes	Random	Minor	Yes	Towards distal tip	Somewhat unclear, possibly multiple (pyrite and flint?)	Strike-a-light and retoucher	Possibly multiple overlapping zones of percussion; sugary texture of the flint makes identifying percussion marks difficult	Cutting soft to medium hard material (butchery?) (right edge, mesio-distal part)	Soressi et al. 2008[6]	S34
**6**	Pech de l'Azé I (Dordogne)	PAI F12-404	Layer 4	Biface	B	Central-Mesial-Distal	Yes	Proximal end	Generally towards distal tip			Minor	Yes	Towards distal tip	Pyrite	Strike-a-light	Sugary texture of the flint makes identifying percussion marks difficult	Cutting soft to medium hard material (butchery?) (left edge, mesio-distal part)	Soressi et al. 2008[6]	S34
**7**	Le Prissé (Pyrénées Atlantiques)	BP 20423	PM2	Biface	A	Center	Yes (n=2)								Unknown	Unknown	Noticeable rounding of flake scar ridges, but minimal associated microwear traces	None observed	Colonge et al. 2015[7]; Brenet et al. 2017[6]	S35
**7**	Le Prissé (Pyrénées Atlantiques)	BP 20423	PM2	Biface	B	Center	Yes					Yes			Unknown	Uncertain (technological?)	Percussion marks may indicate attempt to thin biface via deep flake negative	None observed	Colonge et al. 2015[7]; Brenet et al. 2017[6]	S35
**7**	Le Prissé (Pyrénées Atlantiques)	BP 20746	PM1	Biface	A	Central-Proximal	Yes	Center-Proximal	Generally towards left distal end			Yes	Yes	Roughly parallel to right lateral edge	Unclear	Uncertain		Cutting soft to medium hard material (right edge, distal part)	Colonge et al. 2015[7]; Brenet et al. 2017[6]	S36
**7**	Le Prissé (Pyrénées Atlantiques)	BPR 10 22302	PM1	Biface	A	Distal	Yes	Two zones unrelated to microwear traces in mesial and proximal areas		Yes			Yes	Parallel to right lateral edge	Pyrite? and flint?	Strike-a-light? and retoucher	A few linear gouges appear in percussion zones suggesting brief use as a retoucher.	Cutting soft to medium hard material (left edge, mesio-distal part)	Colonge et al. 2015[7]; Brenet et al. 2017[6]	S37

Supplementary Table S2. Table listing other Middle Palaeolithic sites possessing bifaces with mineral traces on their flat/convex 'faces' that were not included in this study.

# in Fig. 1	Site	Remarks	Relevant literature
**1**	Chez-Pinaud/Jonzac (Charente-Maritime)	One biface (CPN F16-71) with mineral friction traces not included in this study, and five with mineral traces along the edges (CPN E15-118, CPN 205 F13 s3 n8i 4,9, CPN 96 F14 s3 n6 4900, CPN 99 2, CPN E19-612)	Claud 2008[1], 2012[2]
**6**	Pech de l'Azé I (Dordogne)	One bifacial thinning flake with mineral friction traces not included in this study	Soressi et al. 2008[6]
**7**	Le Prissé (Pyrénées Atlantiques)	Two additional bifaces not included in this study exhibiting percussive (No. 21715) and mineral friction traces (No. 11707)	Colonge et al. 2015[7]; Brenet et al. 2017[5]
**8**	Bas-du-Mont des Bruyères (Saint-Amand-les-Eaux)	Ten artefacts (nine bifaces, one bifacial thinning flake) with 14 zones of mineral friction and/or percussion traces	Feray 2014[8]; Claud 2014[9]
**9**	La Quina (Charente-Maritime)	Two bifaces with mineral friction traces	Observed by E. Claud, via S.-J. Park
**10**	Les Bessinaudes (Dordogne)	One biface with percussion traces and ridge rounding	Brenet et al. 2017[5]
**11**	Coursac (Dordogne)	One biface with a cluster of percussion marks	Geneste 1985[3]
**12**	La Rochette (Dordogne)	One biface with mineral friction traces	Claud 2008[1]; Soressi et al. 2008[6]
**13**	Canolle (Dordogne)	Four bifaces and two bifacial thinning flakes exhibiting eight or nine zones with mineral friction and/or percussion traces	Bourguignon (in prep.)[10]
**14**	Les Vieux Coutets (Dordogne)	One biface with percussion traces, linear gouges (likely from flintknapping)	Ortega (in prep.)[11]
**15**	Grotte XVI (Dordogne)	Four bifaces with crushing and/or mineral grinding traces on lateral edges; two of these also possess mineral grinding traces on their flat sides	Soressi and Hays 2003[12]; Soressi 2002[13]
**16**	Latrote (Saint-Gein, Landes)	One biface with a zone of percussion	Brenet et al. 2017[5]
**17**	Bayonne Jupiter (Pyrénées Atlantiques)	Three bifaces, one with mineral friction traces, two with evidence of percussion (flinknapping/retouching)	Colonge et al. 2015[7]
**Not on map**	Assen (The Netherlands)	One biface with evidence of percussion, including heavy crushing and some linear gouging suggesting use as a flintknapping tool	Niekus et al. 2016[14]

Supplementary Table S3. Table listing the experimental tools created for this study and used in combination with various mineral materials for comparison with the archaeological bifaces.

Exp #	Zone	Tool type	Contact material	Location	Time/# of strikes (x)	Biface as the active/passive element	Action	Direction	Special Cleaning	Notes	Fig. #
**3470**		Biface	Pyrite (nodule frag, larger, rounded surface)	Side 1, Right lateral	30 min (10 min intervals)	P	Oblique percussion	Towards proximal end	Sodium bicarbonate	Sparks common, reduced efficacy over time	5c, 6a, S38
**3471**		Biface	Pyrite (nodule frag, small, flattish surface)	Side 1, Left lateral	5 min	P	Oblique percussion	Towards distal tip, transverse to flake edge	Sodium bicarbonate	Sparks common	5a, 6b, S39
**3472**		Biface	Pyrite (half nodule, flat surface)	Side 1, Proximal end, Center	500x	A	Oblique percussion	Proximal end brought down across pyrite surface, parallel to long axis	Sodium bicarbonate	25 sparks captured	5d, 6c, S40
**3473**	A	Biface	Flint (large biface edge)	Side 1, Right lateral	5 min	A	Abrading edge, rubbing back and forth	Parallel to lateral edge			7a, S41
**3473**	B	Biface	Flint (flake)	Side 1, Left lateral	5 min	P	"Backing" flake edge (forceful rubbing)	Towards distal tip, transverse to flake edge		Numerous "percussion" marks where flake dropped abruptly from high to low point	5e, S41
**3473**	C	Biface	Pyrite (aggregate, slightly rounded)	Side 2, Right lateral	5 min	P	Oblique percussion	Towards distal tip, roughly parallel to right lateral edge	Sodium bicarbonate	27 sparks captured	S42
**3473**	D	Biface	Pyrite (nodule frag, flat surface)	Side 2, Left lateral	2 min	P	Oblique percussion	Towards distal tip, roughly parallel to left lateral edge	Sodium bicarbonate	5 sparks captured	6d, S42
**3474**	A	Biface	Sandstone (fine-medium-grained)	Side 1, Right lateral	5 min	P	Rubbing back and forth	Parallel to lateral edge			7e–f, S43
**3474**	B	Biface	Quartz	Side 1, Left lateral	5 min	P	Rubbing back and forth	Parallel to lateral edge			7c–d, S43
**3474**	C	Biface	Iron-cemented sandstone (medium-grained)	Side 2, Left lateral	5 min	P	Rubbing back and forth	Parallel to lateral edge	Oxalic acid 10%	Fair amount of residue still adhering to piece	6f, 7g–h, S44
**3474**	D	Biface	Pyrite (euhedral, flat surface)	Side 2, Right lateral	2 min	P	Oblique percussion	Towards proximal end, roughly parallel to lateral edge	Sodium bicarbonate	1 spark captured, noticeably fewer than other pyrite types	5b, S44
**3475**	A	Biface	Limestone (some sand inclusions, Les Eyzies)	Side 1, Left lateral	5 min	A	Rubbing back and forth	Parallel to lateral edge	HCl, 30 sec		7m–n, S45
**3475**	B	Biface	Pyrite (nodule frag, flat surface)	Side 1, Right lateral	100x (~4 min)	P/A	Forceful rubbing, pulling up biface while pushing down pyrite	Towards distal tip	Sodium bicarbonate	3 sparks captured, not as effective as percussion	6e, S45
**3475**	C	Biface	Pyrite (nodule frag, flattened ridge)	Side 2, Left lateral, more distal	2 min	P	Oblique percussion, light/very glancing	Towards proximal end	Sodium bicarbonate	5 sparks captured, virtually no percussion marks produced	S46
**3475**	D	Biface	Calcareous cortex of flint nodule	Side 2, Right lateral	5 min	A	Rubbing back and forth	Parallel to lateral edge	HCl, 30 sec		7k–l, S46

Exp #	Zone	Tool type	Contact material	Location	Time/# of strikes (x)	Biface as the active/passive element	Action	Direction	Special Cleaning	Notes	Fig. #
**3476**	A	Biface	Flint (larger biface)	Side 1, Left proximal	100x	A	Knapping/retouching	Held distal end up, brought down across knapped edge			5f, S47
**3476**	B	Biface	Flint (flake)	Side 1, Right proximal	200x	P	Unifacial backing, flake active (transverse to flake edge), oblique percussive rubbing	Towards distal end			S47
**3476**	C	Biface	Quartzite (larger chunk, dark gray, medium-grained)	Side 1, Left Mesial-Distal	100x	A	Knapping/retouching	Held distal end up, brought down across knapped edge			7i, S47
**3476**	D	Biface	Quartzite (larger chunk, white, coarser-grained)	Side 1, Right distal	100x	A	Retouching (not too hard)	Held distal end up, brought down across knapped edge			7j, S47
**3476**	E	Biface	Flint (blade)	Side 2, Left proximal	100x	A	Unifacial backing, oblique percussive rubbing transverse to flake edge	Held distal end up, brought down across knapped edge			S48
**3476**	F	Biface	Flint (carinated scraper)	Side 2, Right proximal	100x	A	Unifacial retouching, lighter blows	Held proximal end up, brought down across knapped edge			5g, 7b, S48
**3476**	G	Biface	Pyrite (larger nodule frag); Flint (core)	Side 2, Left distal	3 min, 2 min	P	1st percussion with pyrite; 2nd abrasion with flint over pyrite traces	Towards distal end; rubbing back and forth near lateral edge	Rinsed with water between; Sodium bicarbonate	28 sparks captured	6g, S48
**3477**	A	Biface	Pyrite (large aggregate, rounded surface)	Side 1, Left proximal	3 min	A	Oblique percussion, biface on pyrite	Held distal end up, brought down across across pyrite surface	Sodium bicarbonate	7 sparks captured	S49
**3477**	B	Biface	Meta-quartz (medium sized fragment)	Side 1, Right lateral	2 min	P	Rubbing biface on larger flat surface	Back and forth along lateral edge			S49
**3477**	C	Biface	Flint (large flake)	Side 2, Left mesial-proximal	~200x	A	Knapping, unifacial retouch	Held distal end up, brought down across knapped edge			S50
**3477**	D	Biface	Iron-cemented sandstone (from Les Eyzies, medium-grained)	Side 2, Right lateral	20x, 1 min	P	percussion then rubbing/grinding	Striking towards proximal end, then rubbing back and forth parallel to lateral edge	Oxalic acid 10%	Fair amount of residue still adhering to piece	5h, S50
**3477**	E	Biface	Pyrite (nodule frag)	Side 1, Left distal	5 min	P	Striking (oblique)	Towards distal end	Sodium bicarbonate	9 sparks captured	6h, S49
**3478**	A	Unfacial double-scraper	Hematite	Left lateral edge	10 min	P	Grinding	Rubbing back and forth along lateral edge	Oxalic acid 10%		7o, S51
**3478**	B	Unfacial double-scraper	Hematite	Right lateral edge	5 min	P	Grinding	Rubbing back and forth along lateral edge	Oxalic acid 10%		7p, S51

Exp #	Zone	Tool type	Contact material	Location	Time/# of strikes (x)	Biface as the active/passive element	Action	Direction	Special Cleaning	Notes	Fig. #
**3479**		Unifacial scraper	Goethite	Right lateral edge	5 min	P	Grinding	Rubbing back and forth along lateral edge	Oxalic acid 10%; HCl 10%	Some residue still adhering to piece	7q–r, S52
**3480**		Unifacial scraper	Manganese dioxide (with sandy inclusions)	Dorsal surface, near distal edge	5 min	P	Grinding	Rubbing back and forth along lateral edge	HCl 10% (1 min)		7s, S53
**3481**		Unifacial scraper	Manganese dioxide (intercalated with calcite)	Dorsal surface, near right lateral edge	5 min	P	Grinding	Rubbing back and forth along lateral edge	HCl 10% (1 min)		7t, S54

Supplementary Figures

Archaeological pieces.

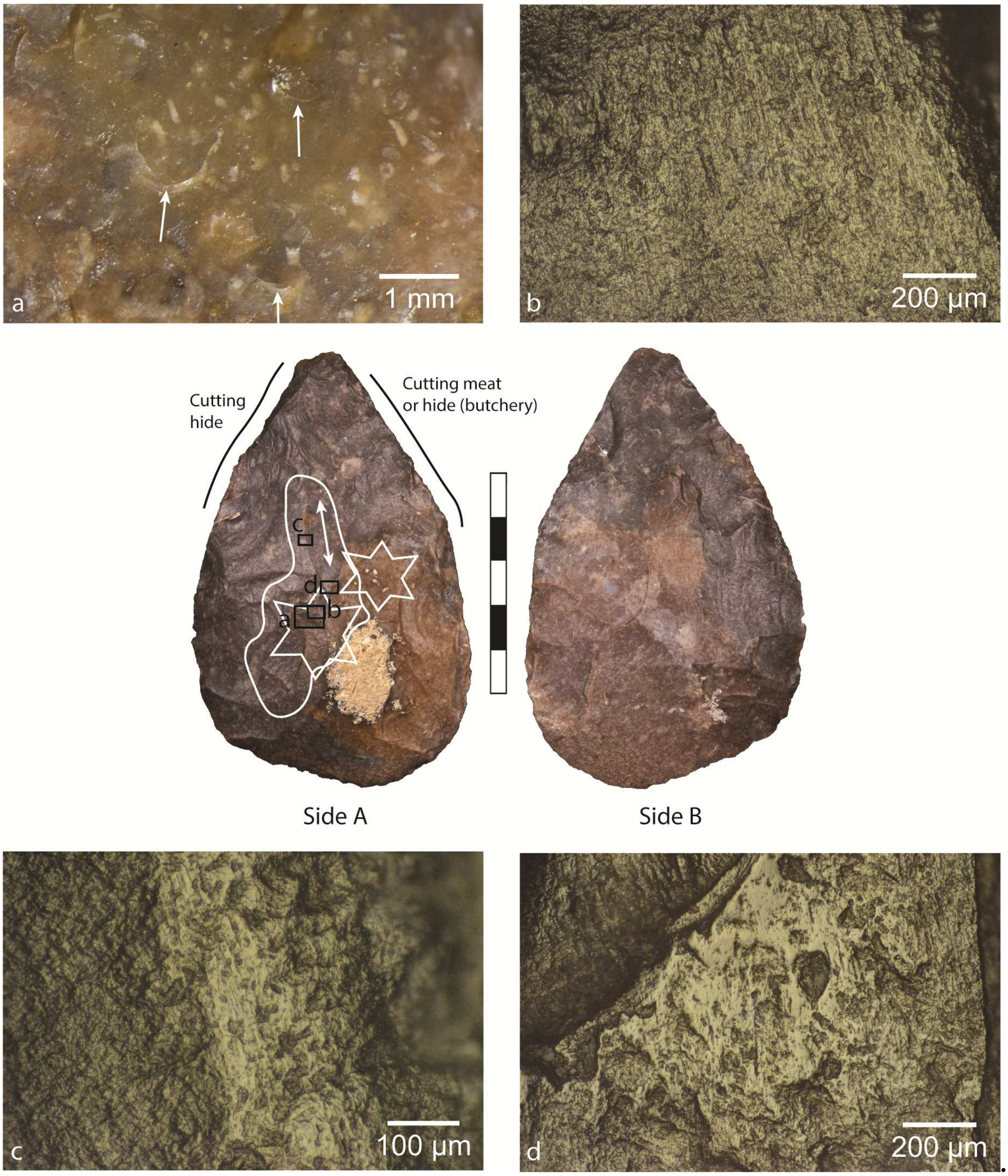

Supplementary Figure S1. Chez-Pinaud/Jonzac biface CPN 03 D18 s4 7s 5,55-5,60 (Charente-Maritime). The white line demarcates the zone of mineral use-wear traces somewhat similar to pyrite. The stars indicate two zones of percussion. The right cluster contains a few linear gouges suggesting brief use as a retoucher, while the left cluster contains C-shaped marks opening distally and some crushing along a flake scar ridges that are likely associated with the mineral traces. The arrow indicates the orientation of striations. Other observed microwear traces are indicated. a) Low-magnification image of distally opening C-shaped percussion marks. b) High-magnification images of striations and weakly developed mineral polish. c) High-magnification image of more well-developed mineral polish and striations along flake scar ridge. d) High-magnification image of isolated zone of very bright polish, possibly taphonomic resulting from prolonged contact with another flint artefact post-burial.

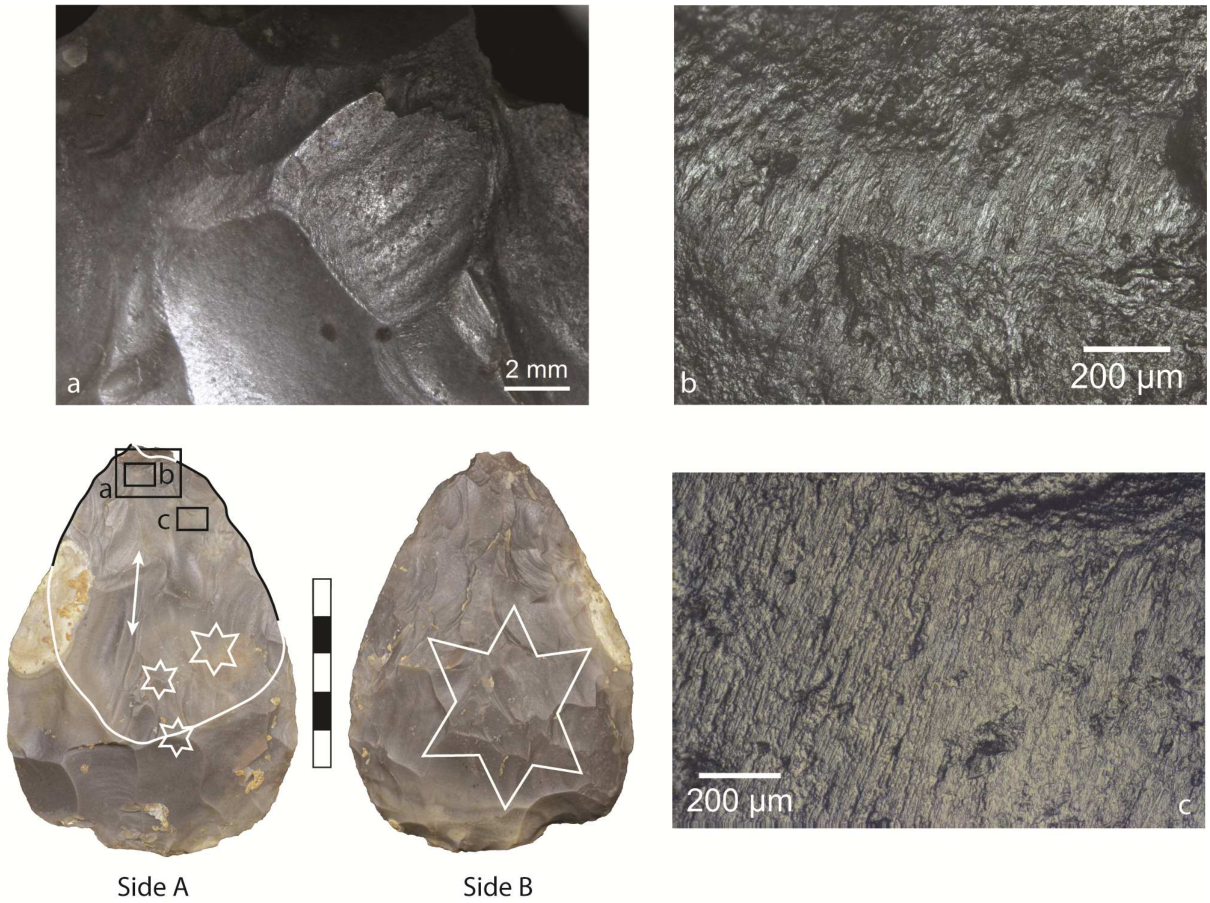

Supplementary Figure S2. Chez-Pinaud/Jonzac biface CPN 99 W9 (Charente-Maritime). The white and black line demarcates the zone of mineral use-wear traces comparable to pyrite. The arrow indicates the orientation of striations. The stars indicate zones of percussion. The small clusters on Side A contain only a few small percussion marks, possibly related to the mineral traces. The large cluster of percussion marks on Side B also contains linear gouges suggesting use as a retoucher. a) Low-magnification image showing rounding of the flake scar ridges. b) High-magnification image of mineral polish (fairly poorly developed) and striations along rounded flake scar ridge. c) High-magnification image of mineral polish and striations on flatter surface.

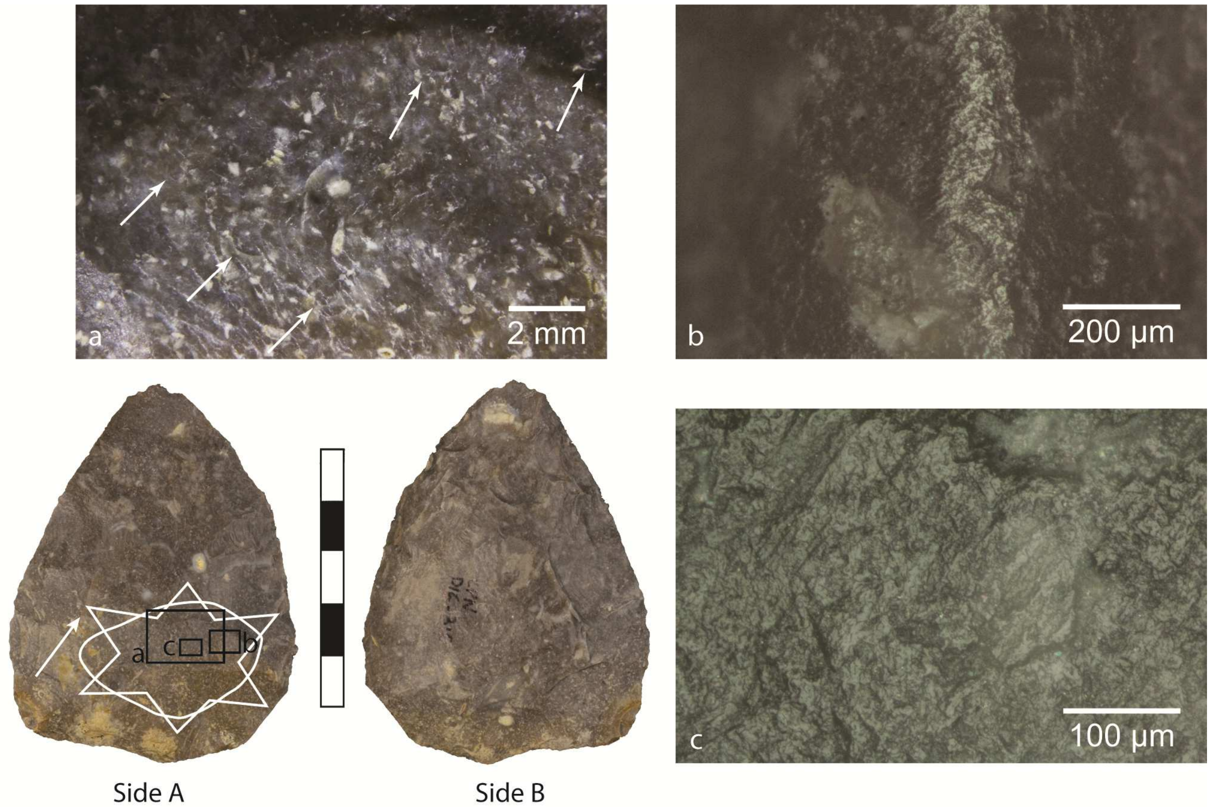

Supplementary Figure S3. Chez-Pinaud/Jonzac biface CPN D16-316 (Charente-Maritime). The white line demarcates the zone of mineral use-wear traces somewhat similar to pyrite, though this interpretation is limited given the poor development of the traces. The star indicates a zone of percussion containing C-shaped marks opening towards the distal end and right lateral edge, in line with the orientation of the striations (arrow). a) Low-magnification image of distally opening C-shaped percussion marks, indicated by the arrows which also indicate their directionality. b & c) High-magnification images of striations and associated weakly developed mineral polish.

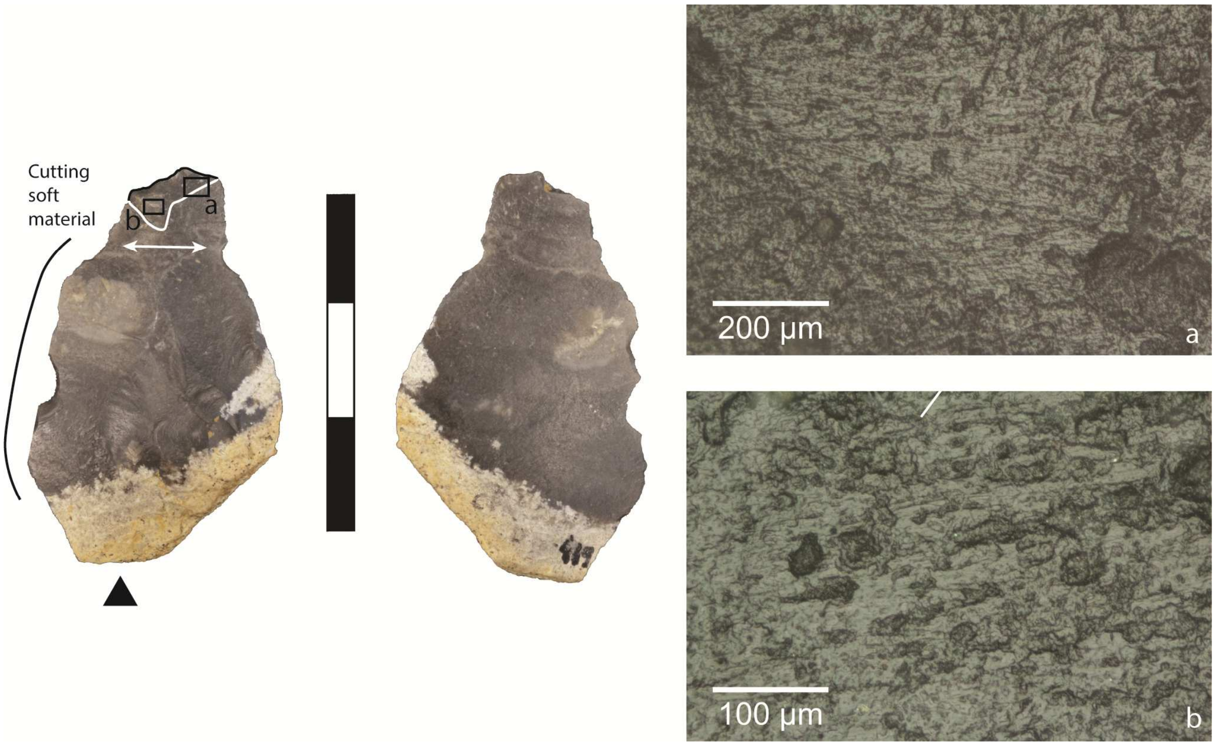

Supplementary Figure S4. Chez-Pinaud/Jonzac bifacial thinning flake CPN D16-486 (Charente-Maritime). The white and black line demarcates the zone of mineral use-wear traces comparable to pyrite. The arrow indicates the orientation of striations. Other observed microwear traces are indicated. a & b) High-magnification images of mineral polish and striations.

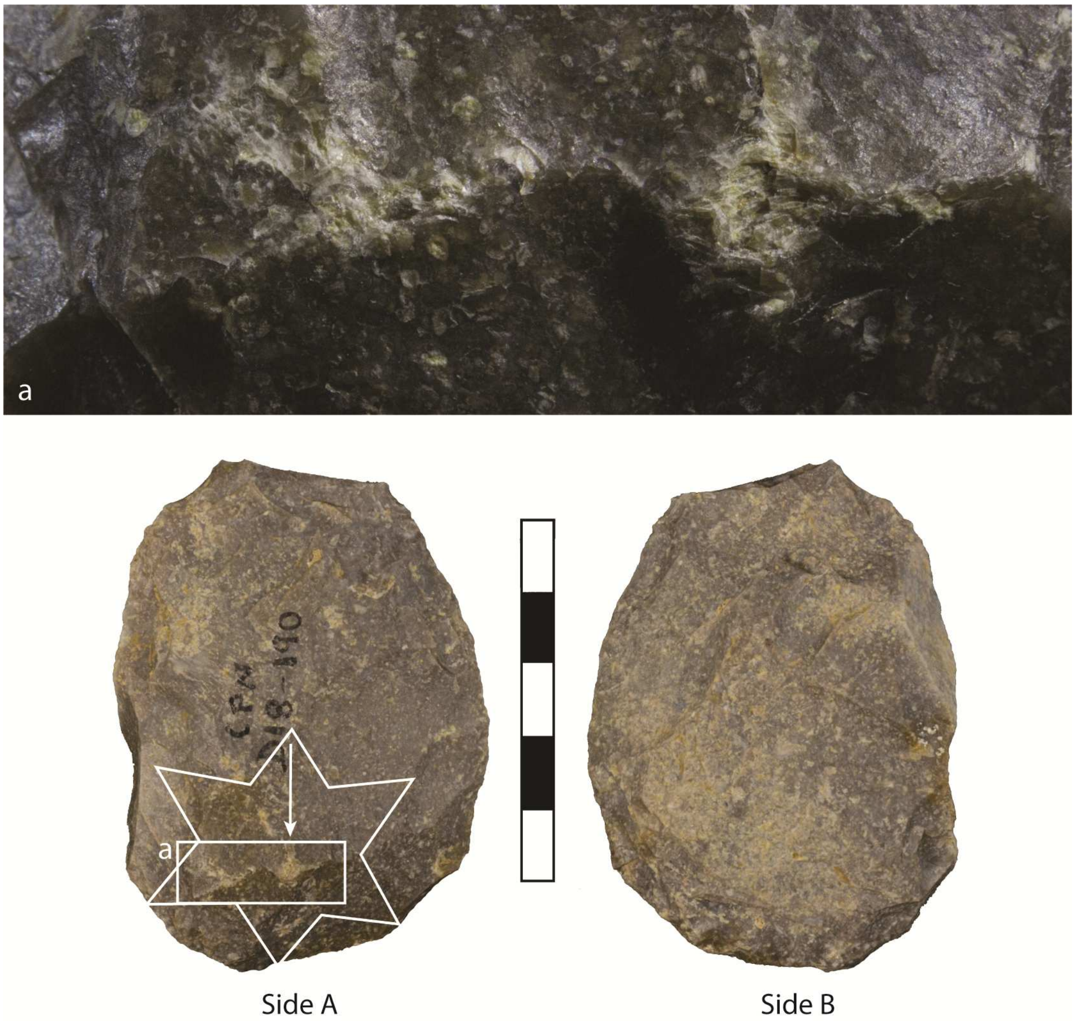

Supplementary Figure S5. Chez-Pinaud/Jonzac biface CPN D18-190 (Charente-Maritime). The star indicates the zone of percussion and associated crushing concentrated along a flake scar ridge produced during some unknown pounding activity, possibly flintknapping due to presence of a few linear gouges. The arrow indicates the probably direction of force due do some C-shaped percussion marks opening proximally and removals occurring on the proximal side of the ridge. There are no associated mineral traces. a) Low-magnification image of percussion marks and crushing.

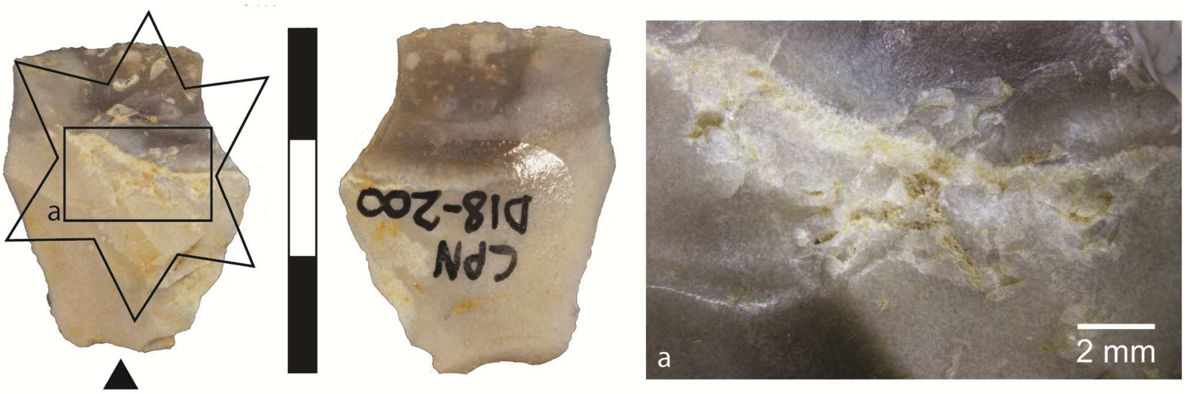

Supplementary Figure S6. Chez-Pinaud/Jonzac bifacial thinning flake CPN D18-200 (Charente-Maritime). The star indicates the zone of percussion (linear gouges/surface removals) concentrated along a flake scar ridge likely produced while flintknapping. There are no associated mineral traces. a) Low-magnification image of the surface removals/gouges.

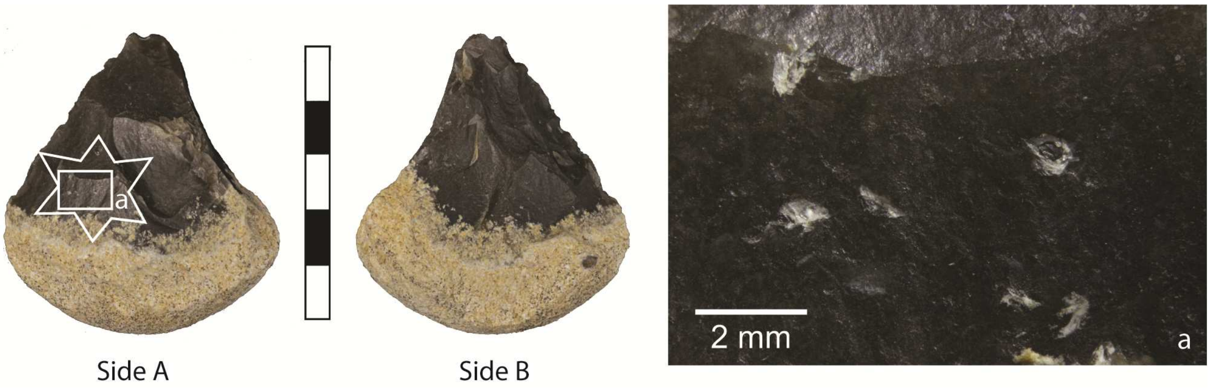

Supplementary Figure S7. Chez-Pinaud/Jonzac biface CPN D19-823 (Charente-Maritime). The star indicates the zone of percussion and associated surface gouges likely produced while flintknapping. There are no associated mineral traces. a) Low-magnification image of linear and ovate surface gouges.

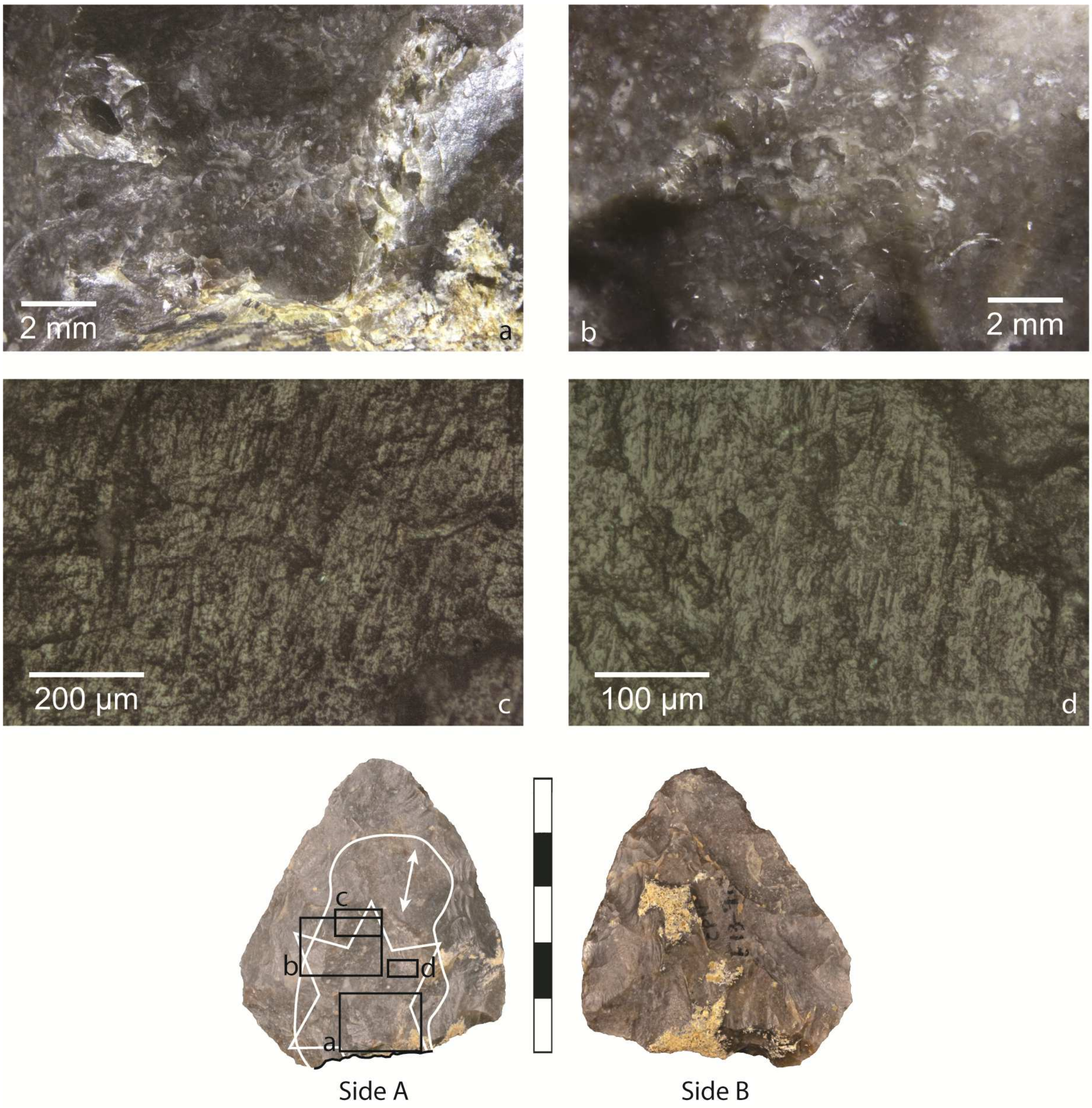

Supplementary Figure S8. Chez-Pinaud/Jonzac biface CPN E13-718 (Charente-Maritime). The white line demarcates the zone of mineral use-wear traces somewhat similar to pyrite. The arrow indicates the orientation of striations. The star indicates a zone of percussion containing percussion marks with ambiguous directionalities (b) and a zone of heavy crushing near the proximal end (a). The presence of a few linear gouges suggests this zone may have been used both for flintknapping/retouching and fire making. c & d) High-magnification images of striations and associated moderately developed mineral polish.

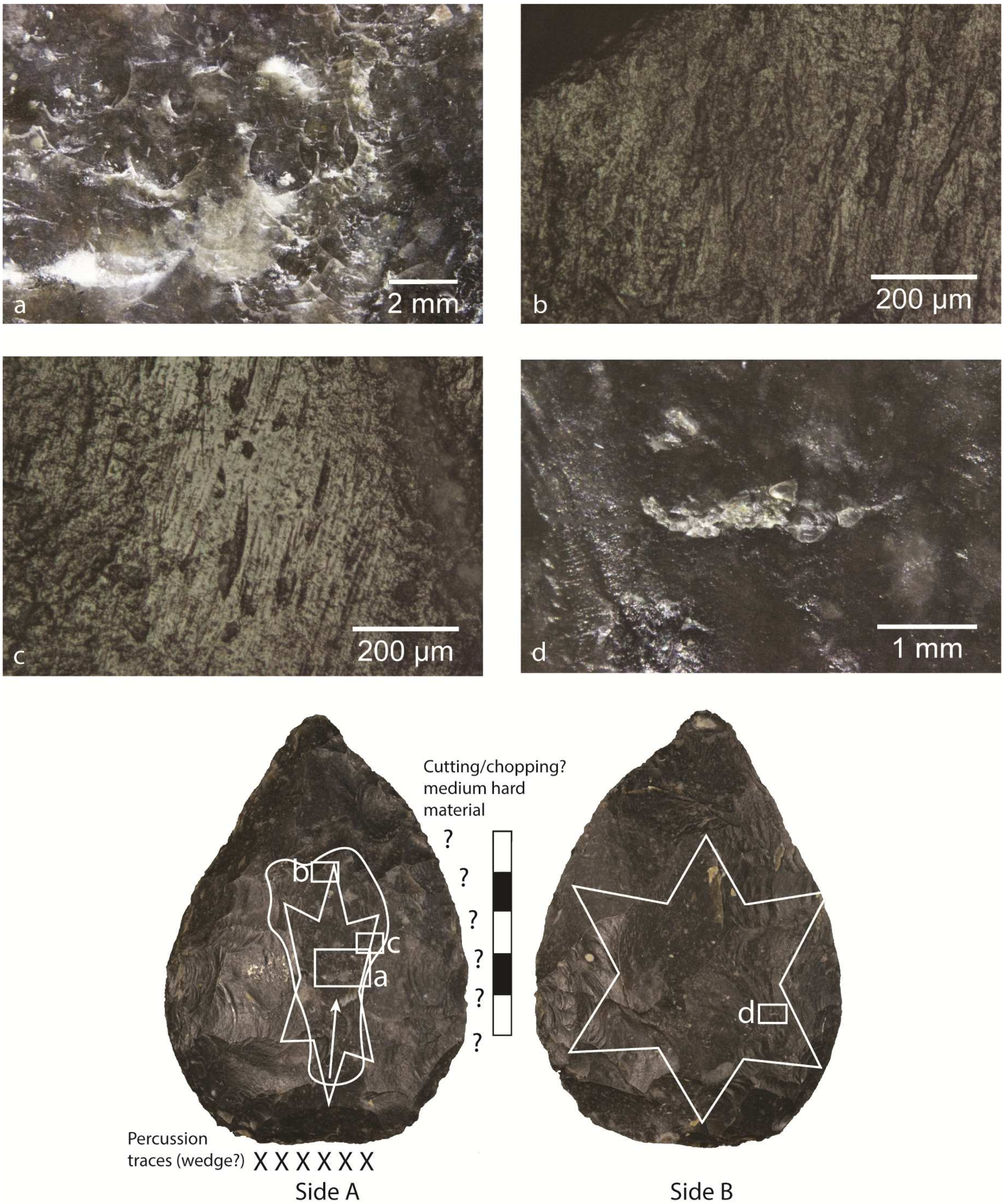

Supplementary Figure S9. Chez-Pinaud/Jonzac biface CPN E13-748 (Charente-Maritime). The white line demarcates the zone of mineral use-wear traces comparable to pyrite. The star on Side A indicates a zone of percussion containing numerous C-shaped percussion marks that open distally (a) in good agreement with the striations (arrow). On Side B, the star encompasses a zone of percussion containing multiple linear gouges (d) indicating this surface was used for retouching/flintknapping. b & c) High-magnification images of mineral polish and striations. Other observed microwear traces are indicated.

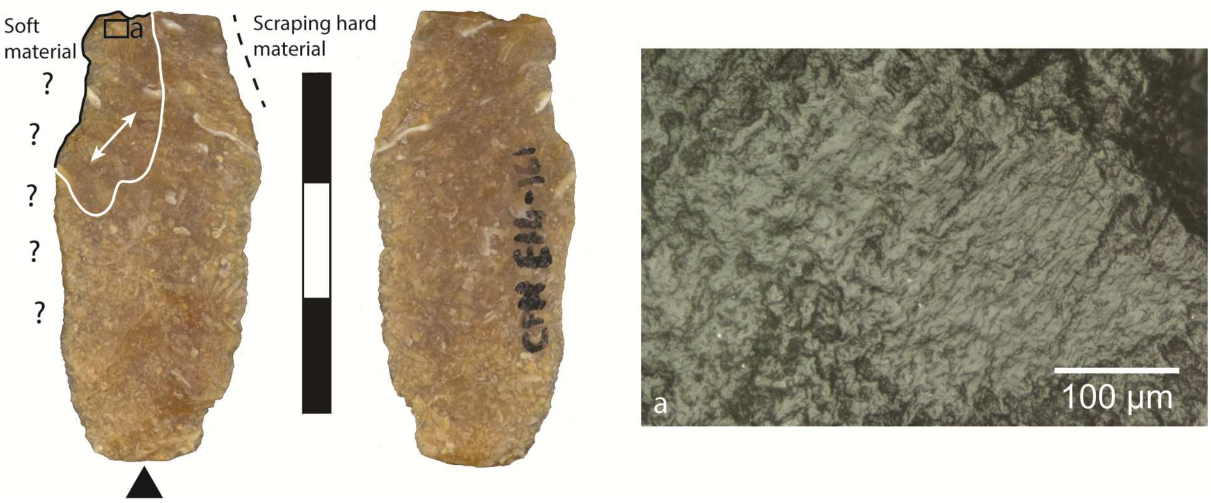

Supplementary Figure S10. Chez-Pinaud/Jonzac bifacial thinning flake CPN E14-161 (Charente-Maritime). The white and black line demarcates the zone of mineral use-wear traces comparable to pyrite. The arrow indicates the orientation of striations. Other observed microwear traces are indicated. a) High-magnification image of mineral polish and striations.

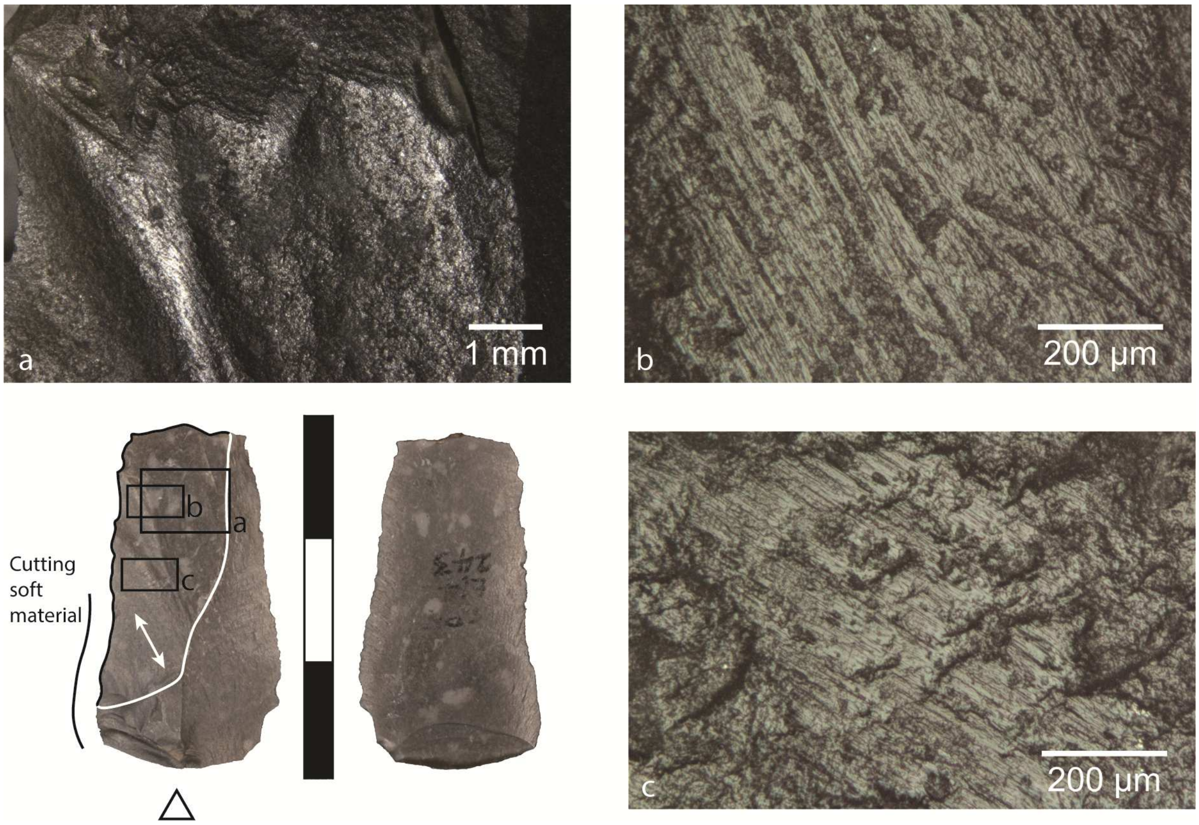

Supplementary Figure S11. Chez-Pinaud/Jonzac bifacial thinning flake CPN E14-243 (Charente-Maritime). The white and black line demarcates the zone of well-developed mineral use-wear traces comparable to pyrite. The arrow indicates the orientation of striations. Other observed microwear traces are indicated. a) Low-magnification image of the surface highlights the heavy rounding of flake scar ridges. b & c) High-magnification images of well-developed mineral polish, striations and slightly wider and deeper surface scratches.

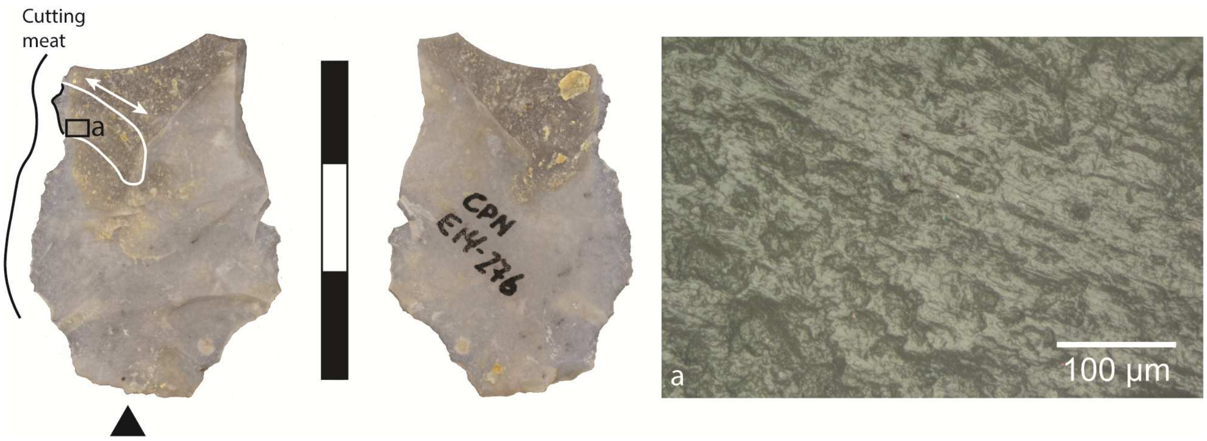

Supplementary Figure S12. Chez-Pinaud/Jonzac bifacial thinning flake CPN E14-276 (Charente-Maritime). The white and black line demarcates the zone of moderately to weakly developed mineral use-wear traces somewhat similar to pyrite. The arrow indicates the orientation of striations. Other observed microwear traces are indicated. a) High-magnification images of mineral polish and striations.

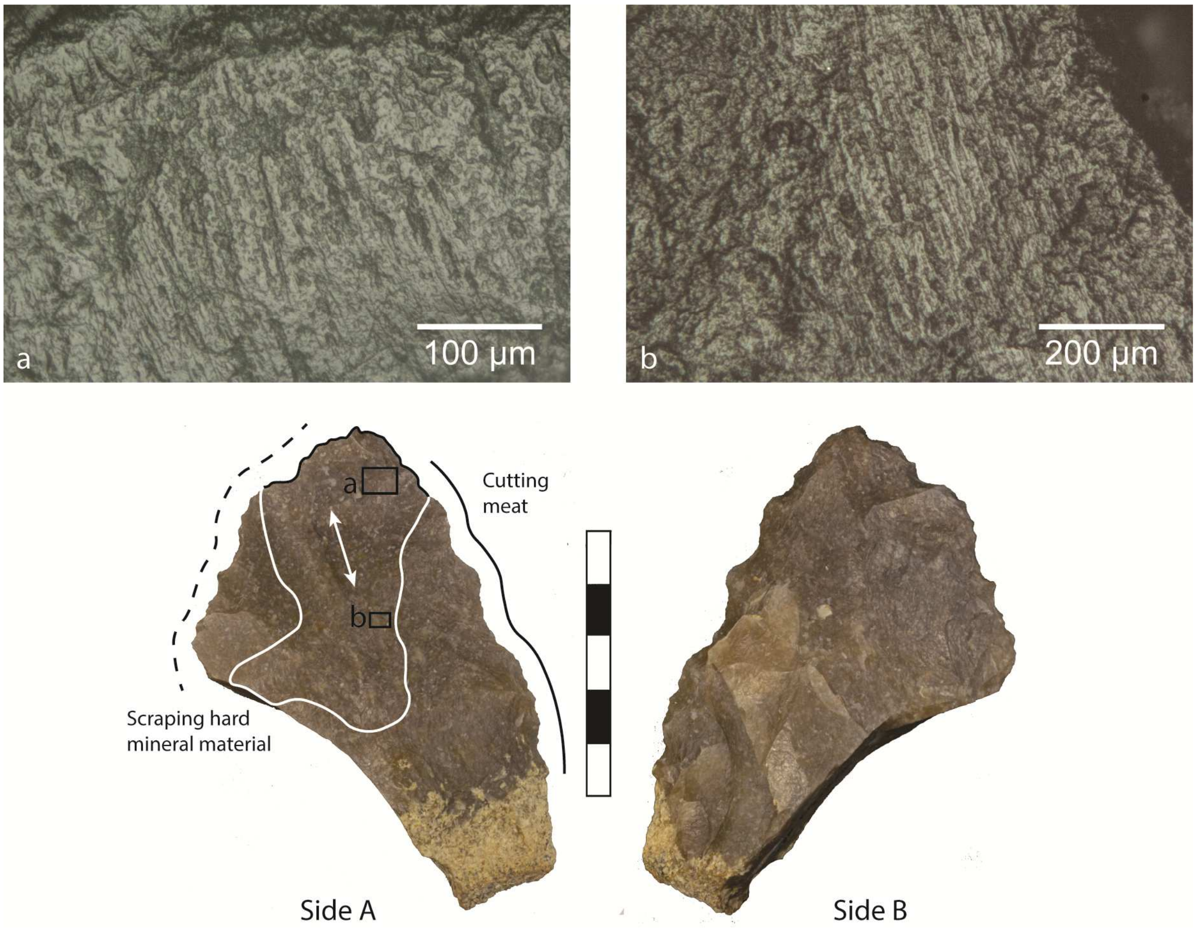

Supplementary Figure S13. Chez-Pinaud/Jonzac biface fragment CPN E15-324 (Charente-Maritime). The white and black line demarcates the zone of mineral use-wear traces comparable to pyrite. The arrow indicates the orientation of striations. Other observed microwear traces are indicated. a & b) High-magnification images of mineral polish and striations.

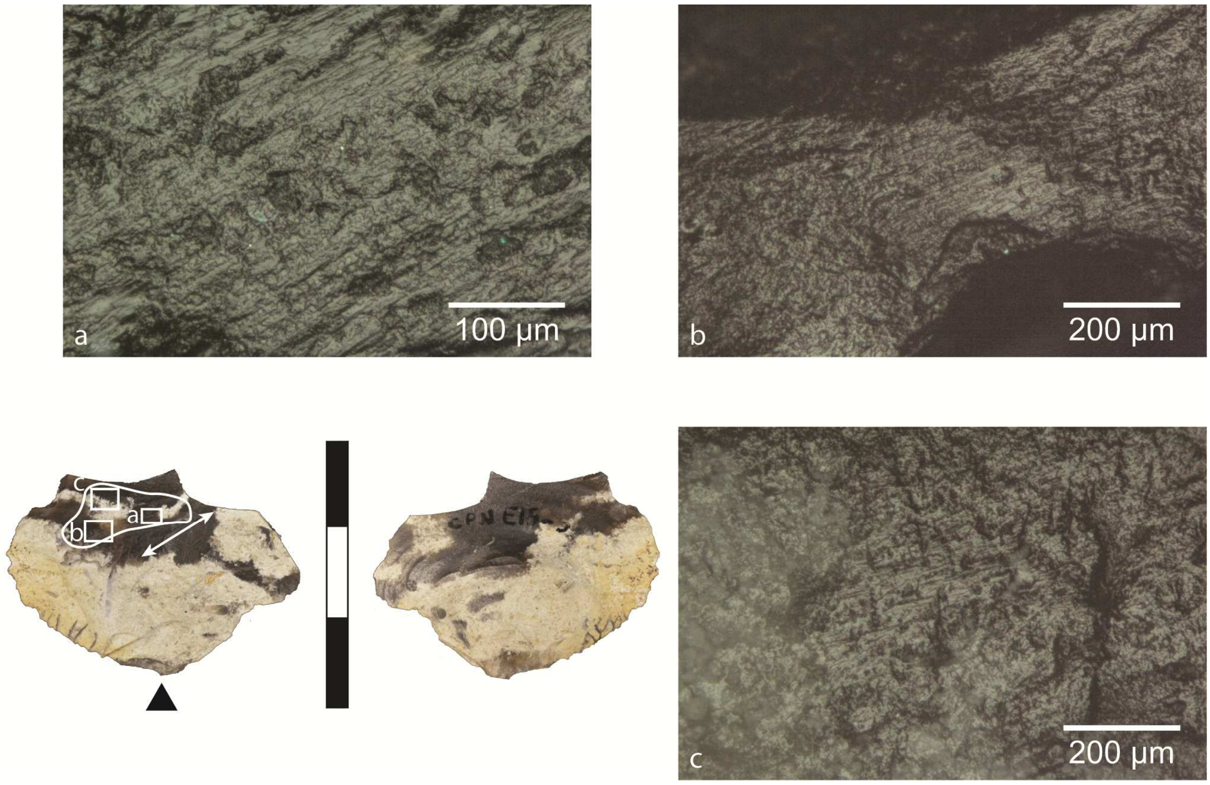

Supplementary Figure S14. Chez-Pinaud/Jonzac bifacial thinning flake CPN E15-370 (Charente-Maritime). The white line demarcates the zone of mineral use-wear traces comparable to pyrite. The arrow indicates the orientation of striations. a–c) High-magnification images of mineral polish and striations.

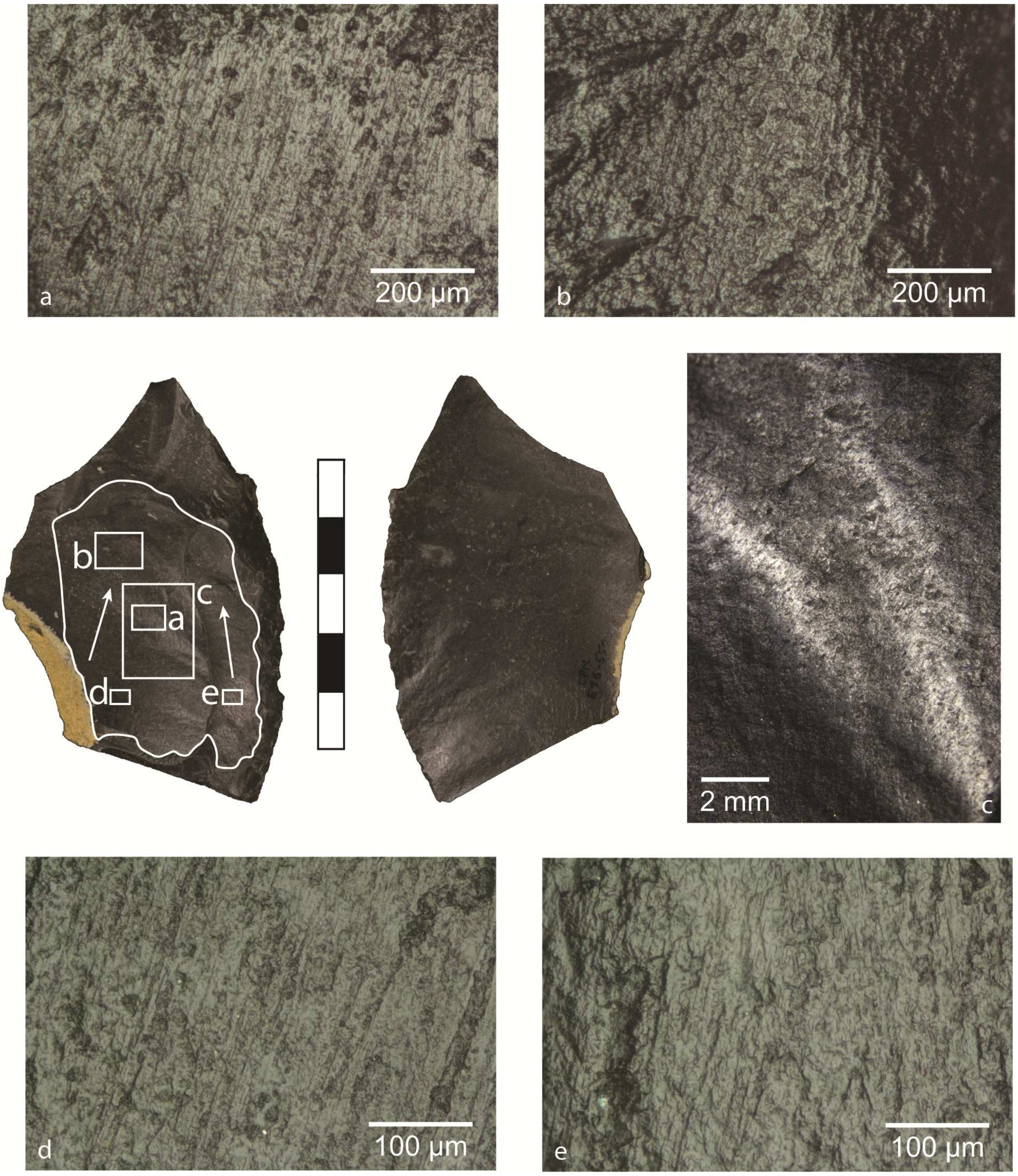

Supplementary Figure S15. Chez-Pinaud/Jonzac retouched bifacial thinning flake CPN E16-550 (Charente-Maritime). The white line demarcates the zone of mineral use-wear traces comparable to pyrite. The arrows indicate the slightly variable orientations of striations, possibly indicating more than one use episodes. c) Low-magnification image of the surface showing extent of ridge rounding and bright polish, possibly enhanced during use by prehension polish. a, b, d, e) High-magnification images of mineral polish and striations.

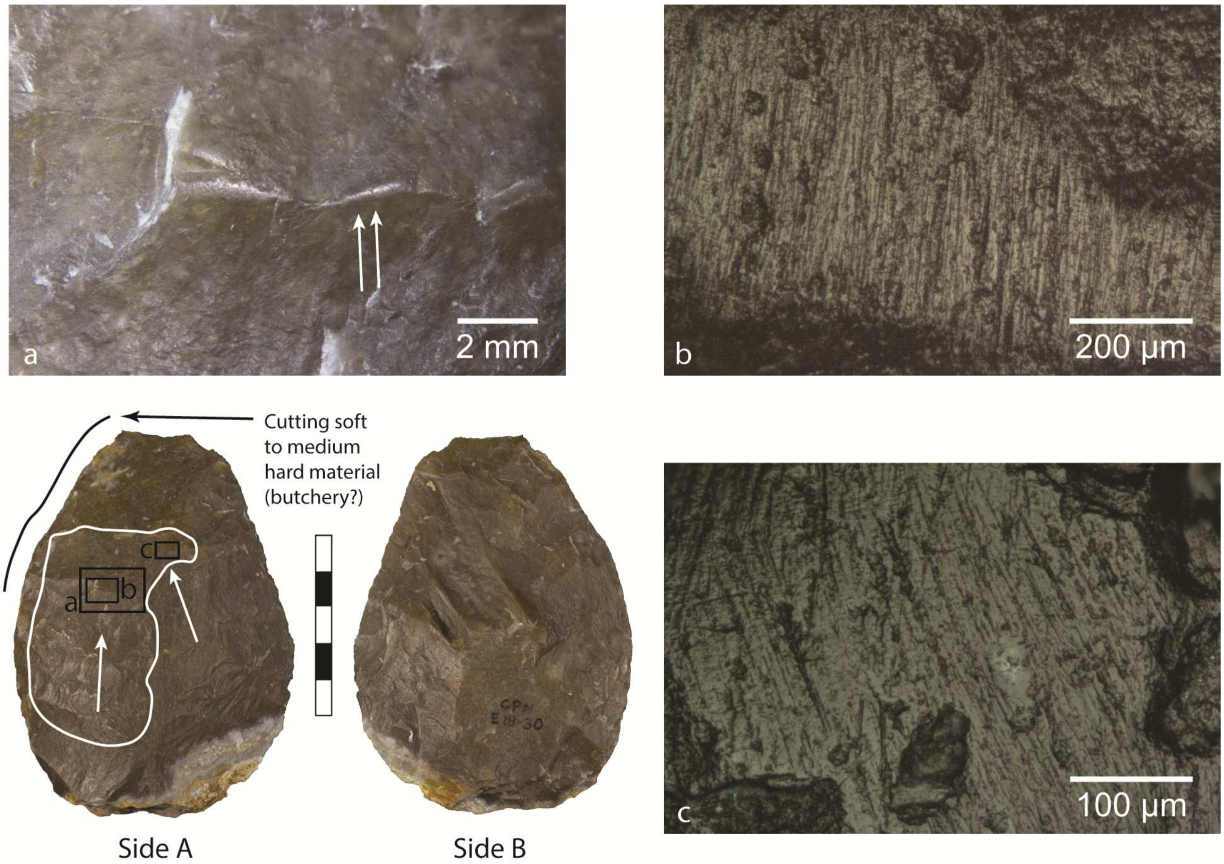

Supplementary Figure S16. Chez-Pinaud/Jonzac biface CPN E18-30 (Charente-Maritime). The white line demarcates the zone of mineral use-wear traces comparable to pyrite. The arrows indicate the slightly variable orientations of striations, possibly indicating more than one use episode. Other observed microwear traces are indicated. a) Low-magnification image of the surface showing extent of ridge rounding. Arrows indicate two small (difficult to see) distally opening percussion marks. a) High-magnification image of planed flake scar ridge with well-developed mineral polish and striations. b) High-magnification image of well-developed mineral polish and intersecting striations of different directionalities.

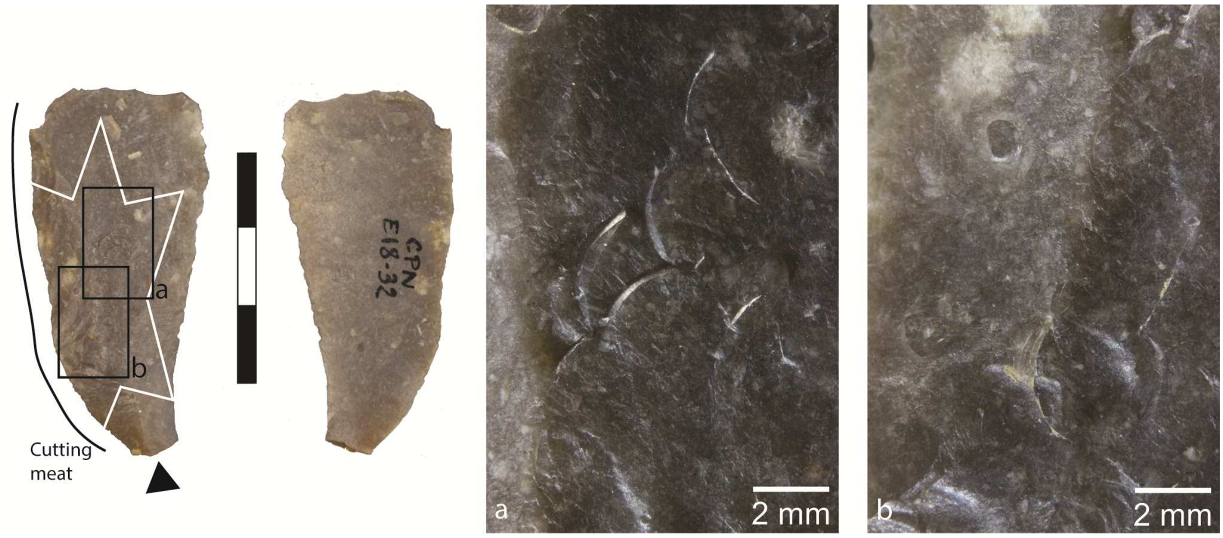

Supplementary Figure S17. Chez-Pinaud/Jonzac bifacial thinning flake CPN E18-32 (Charente-Maritime). The star indicates a zone of percussion, with the left flake scar containing percussion marks

with variable directionalities (a), and the right flake scar possessing numerous truncated percussion marks (b). There are no associated mineral traces. Other observed microwear traces are indicated.

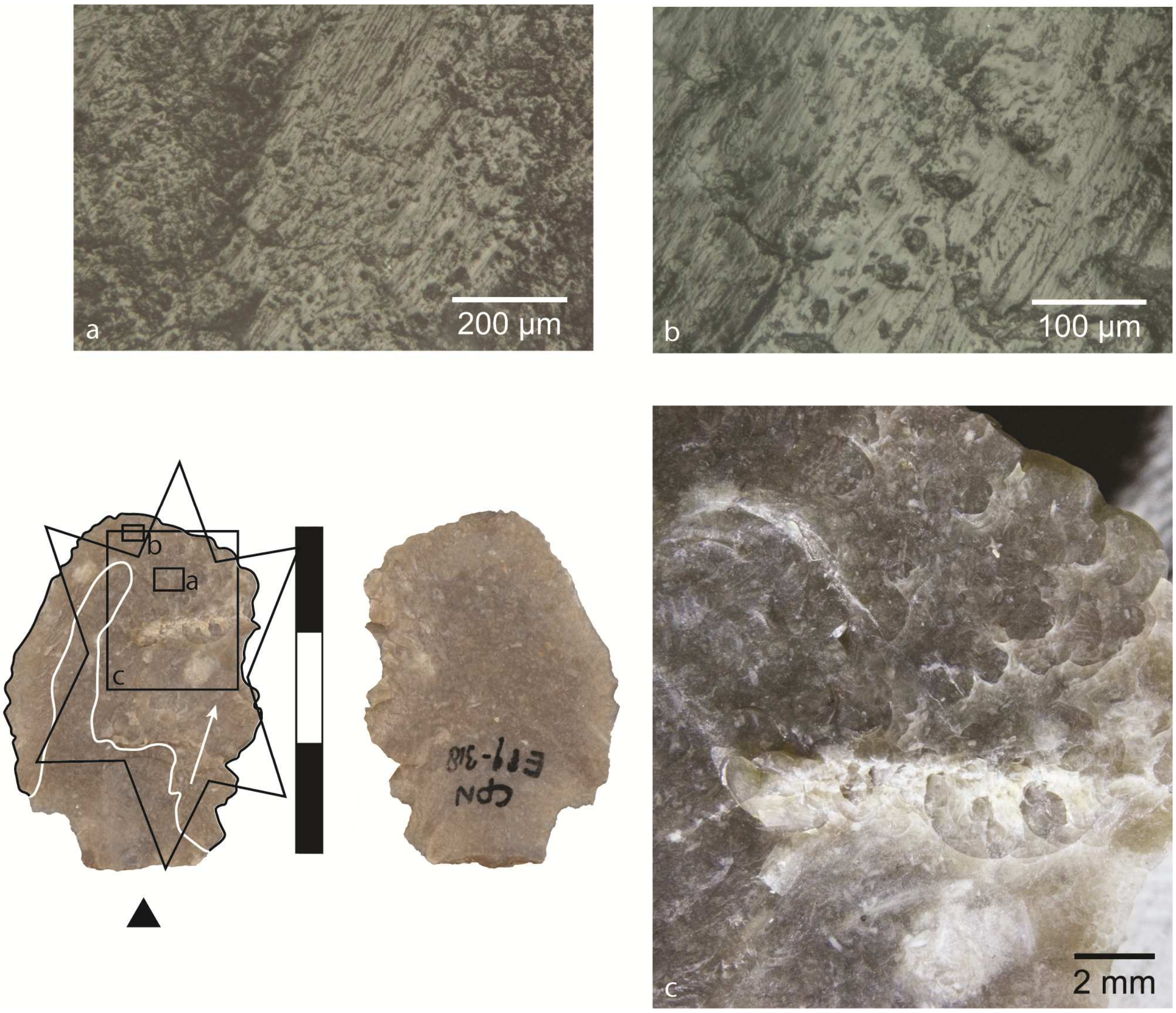

Supplementary Figure S18. Chez-Pinaud/Jonzac bifacial thinning flake CPN E19-318 (Charente-Maritime). The white and black line demarcates the zone of mineral use-wear traces comparable to pyrite. The arrow indicates the orientation of the striations. The star indicates these traces are located within a zone of heavy percussion and crushing (c), the percussion mark directionalities being variable, though many open distally in agreement with the striations. a & b) High-magnification images of well-developed mineral polish and striations.

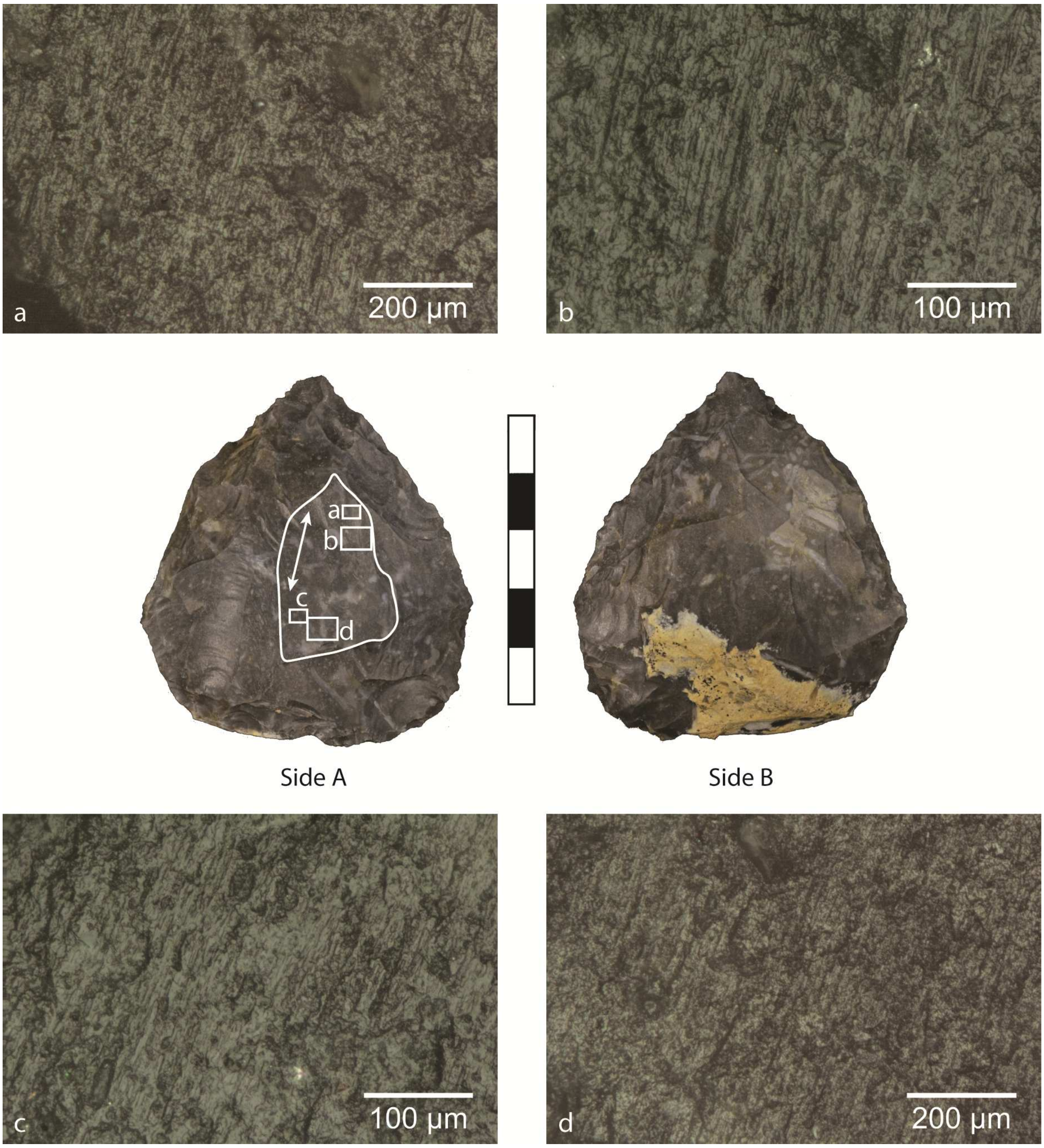

Supplementary Figure S19. Chez-Pinaud/Jonzac biface CPN E19-425 (Charente-Maritime). The white line demarcates the zone of mineral use-wear traces comparable to pyrite. The arrow indicates the orientation of the striations. a–d) High-magnification images of mineral polish and striations.

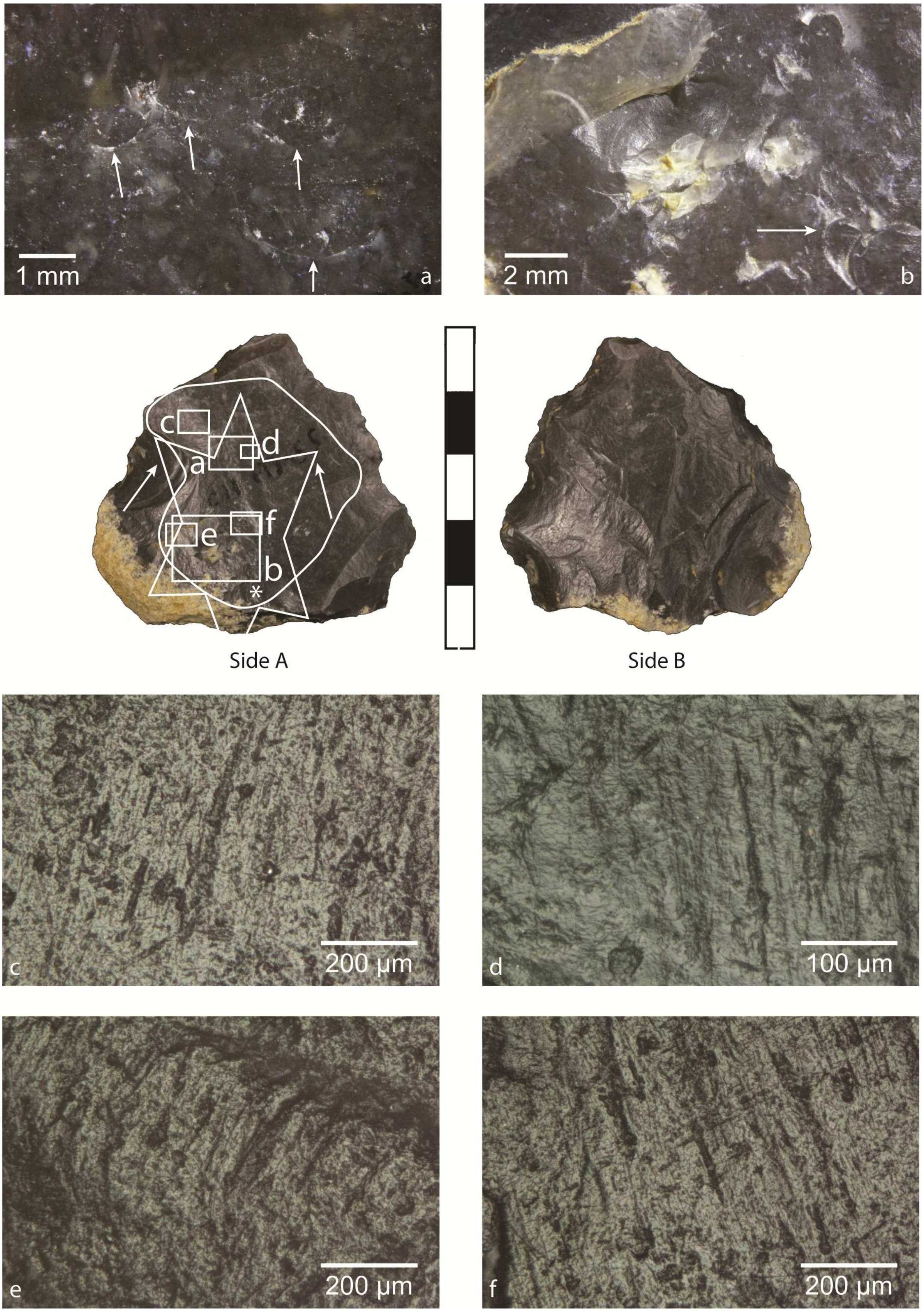

Supplementary Figure S20. Chez-Pinaud/Jonzac biface CPN F15-55 (Charente-Maritime). The white line demarcates the zone of mineral use-wear traces comparable to pyrite. The arrow indicates the variable orientations of the striations, suggesting more than one use episodes. The star indicates these traces are located within a zone of percussion, the C-shaped percussion marks oriented towards the

distal end (a) likely related to fire making (the arrows in the image indicating the locations and directionalities of the percussion marks), and the more variable direction percussion marks and linear gouges (b) suggesting the biface was also used for flintknapping/retouching (the asterisk on the bifaces and arrow in the low-magnification image points to a zone of percussion marks truncated by a later flake removal). c–f) High-magnification images of well-developed mineral polish and striations showing variable directionalities. The particularly deep striations may be related to the flintknapping/retouching activities.

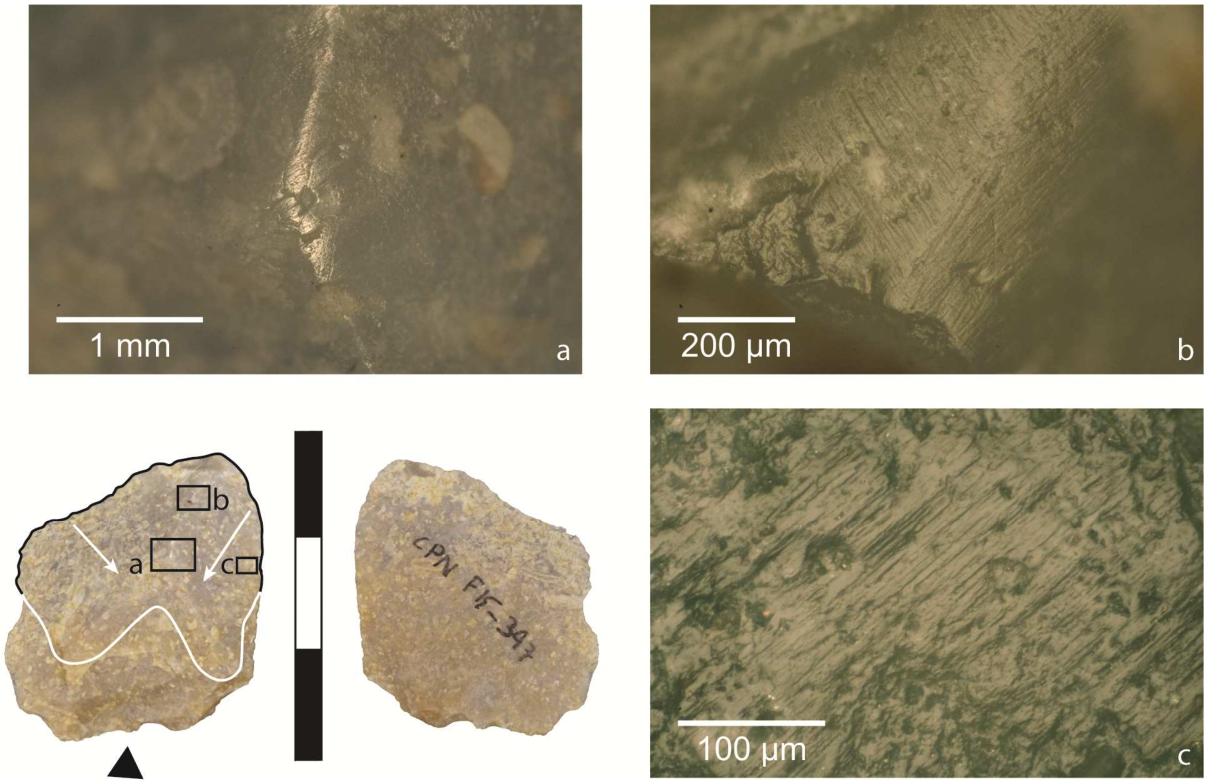

Supplementary Figure S21. Chez-Pinaud/Jonzac bifacial thinning flake CPN F15-397 (Charente-Maritime). The white and black line demarcates the zone of mineral use-wear traces comparable to pyrite. The arrows indicate the variable orientations of the striations, suggesting at least two episodes of use. a) Low-magnification image of rounding along a flake scare ridge and associated polish. b & c) High-magnification images of well-developed mineral polish and striations, with image b showing a location where the variably oriented striations intersect.

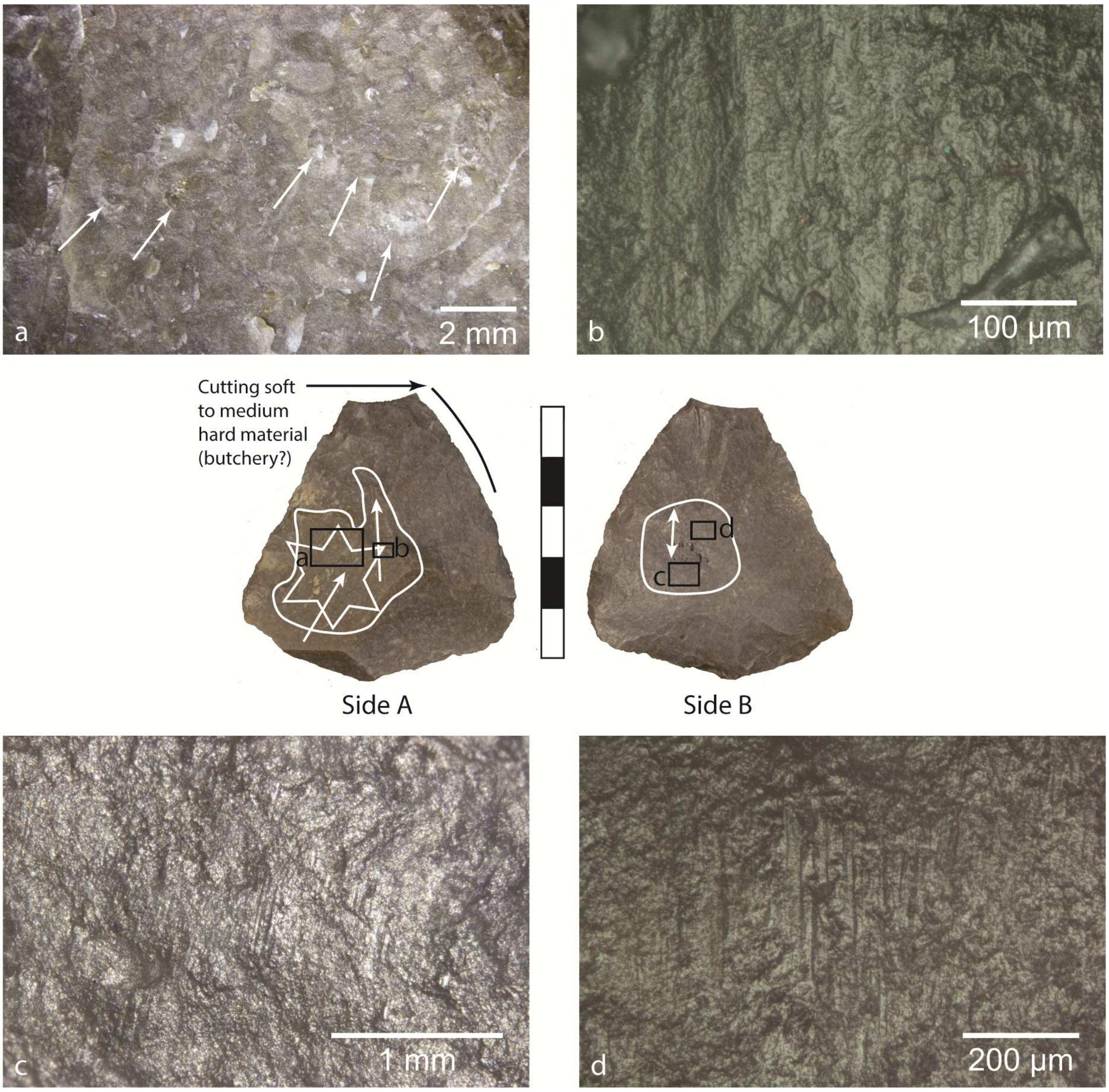

Supplementary Figure S22. Chez-Pinaud/Jonzac biface CPN F16-73 (Charente-Maritime). The white lines demarcate the zones of mineral use-wear traces. The arrows indicate the orientations of the striations, the bi-directional nature of the striations on Side A possibly suggesting at least two episodes of use. The star indicates these traces are associated with a zone of percussion, the generally C-shaped percussion marks on Side A (a) opening towards the right ride of the distal end (arrows in the image indicate the locations and directionality of these percussion marks). b) High-magnification image of deep, wide grooves/striations associated with weakly developed mineral polish, somewhat similar to experimental traces resulting from flintknapping/retouching quartzite. Quartzite results in minimal surface gouging compared to knapping flint. Low-magnification image (c) and high-magnification image (d) of deep linear grooves/striations on Side B. The isolated nature of the traces on Side B, along with absence of associated mineral polish and percussion marks suggests use of this side was minimal. Other observed microwear traces are indicated.

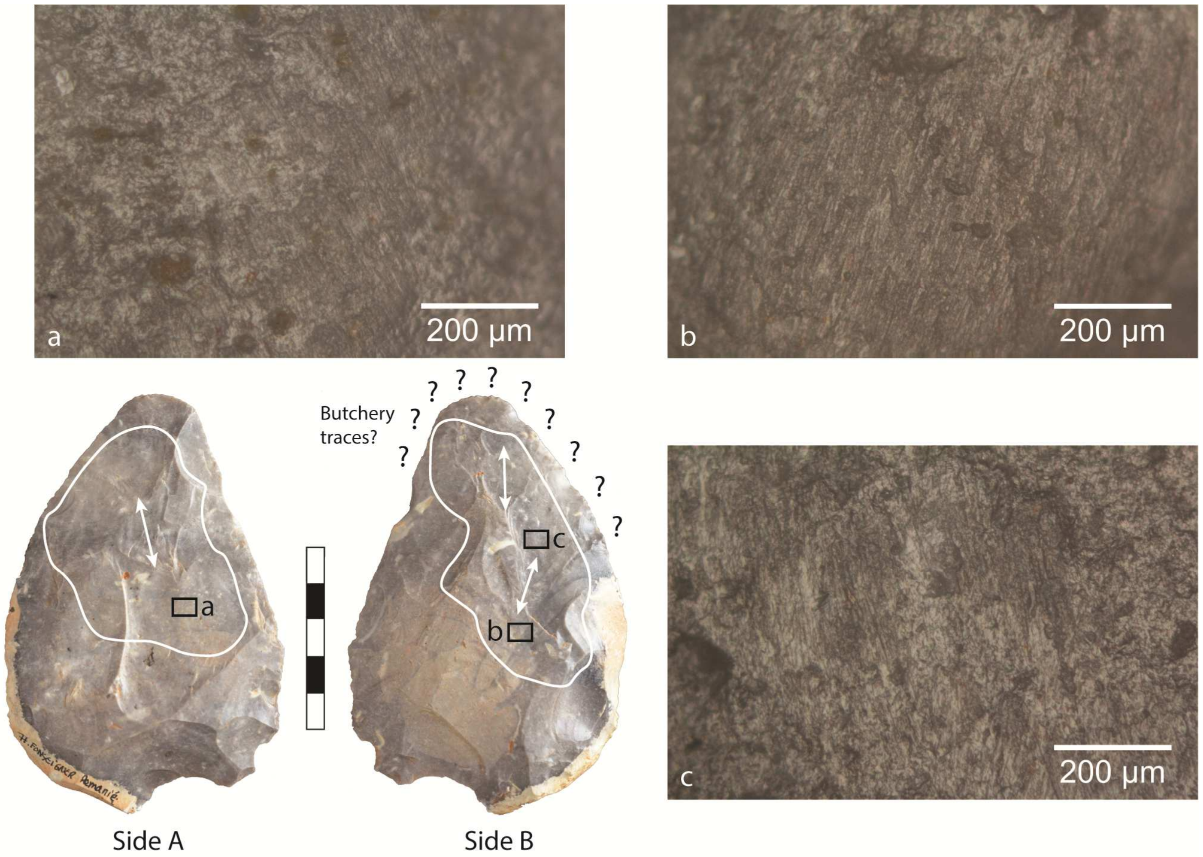

Supplementary Figure S23. Fonseigner 77-31 biface (Dordogne). The white lines demarcate the zones of mineral use-wear traces comparable to pyrite. The arrows indicate the orientations of the striations, the bi-directional nature of the striations on Side B possibly suggesting at least two episodes of use. a) High-magnification image of mineral polish and striations on Side A. b & c) High-magnification images of mineral polish and striations, with image c showing a location where the variably oriented striations overlap. Polish possibly related to prehension is present on the proximal half of the biface on both sides.

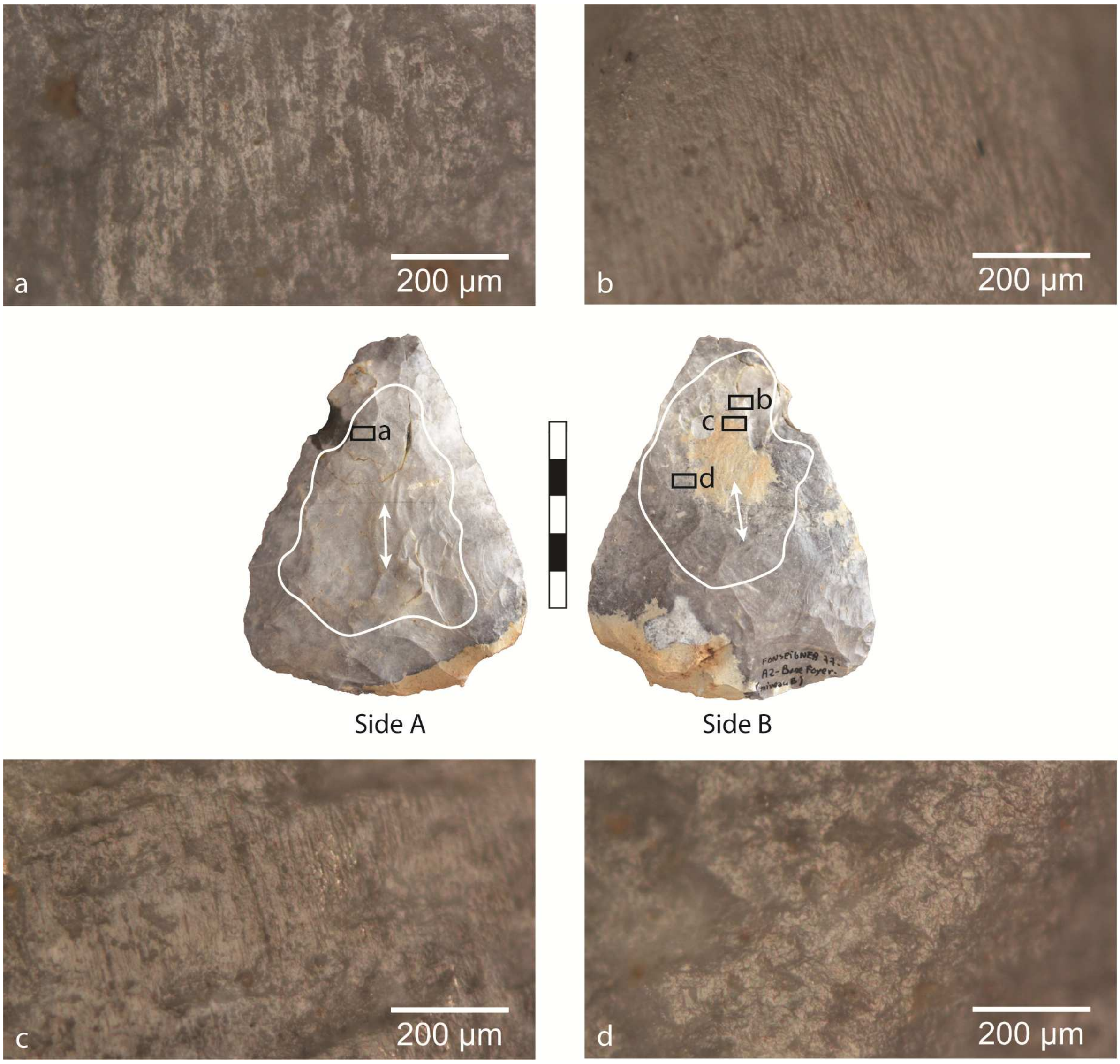

Supplementary Figure S24. Fonseigner 77-A2 biface (Dordogne). The white lines demarcate the zones of mineral use-wear traces comparable to pyrite, with notable rounding of flake scar ridges. The arrows indicate the orientations of the striations. a) High-magnification image of mineral polish and striations on Side A. b & c) High-magnification images of mineral polish and striations on Side B. d) High-magnification image of polish possibly resulting from prehension. Possible prehension polish is present on the proximal half of the biface on both sides.

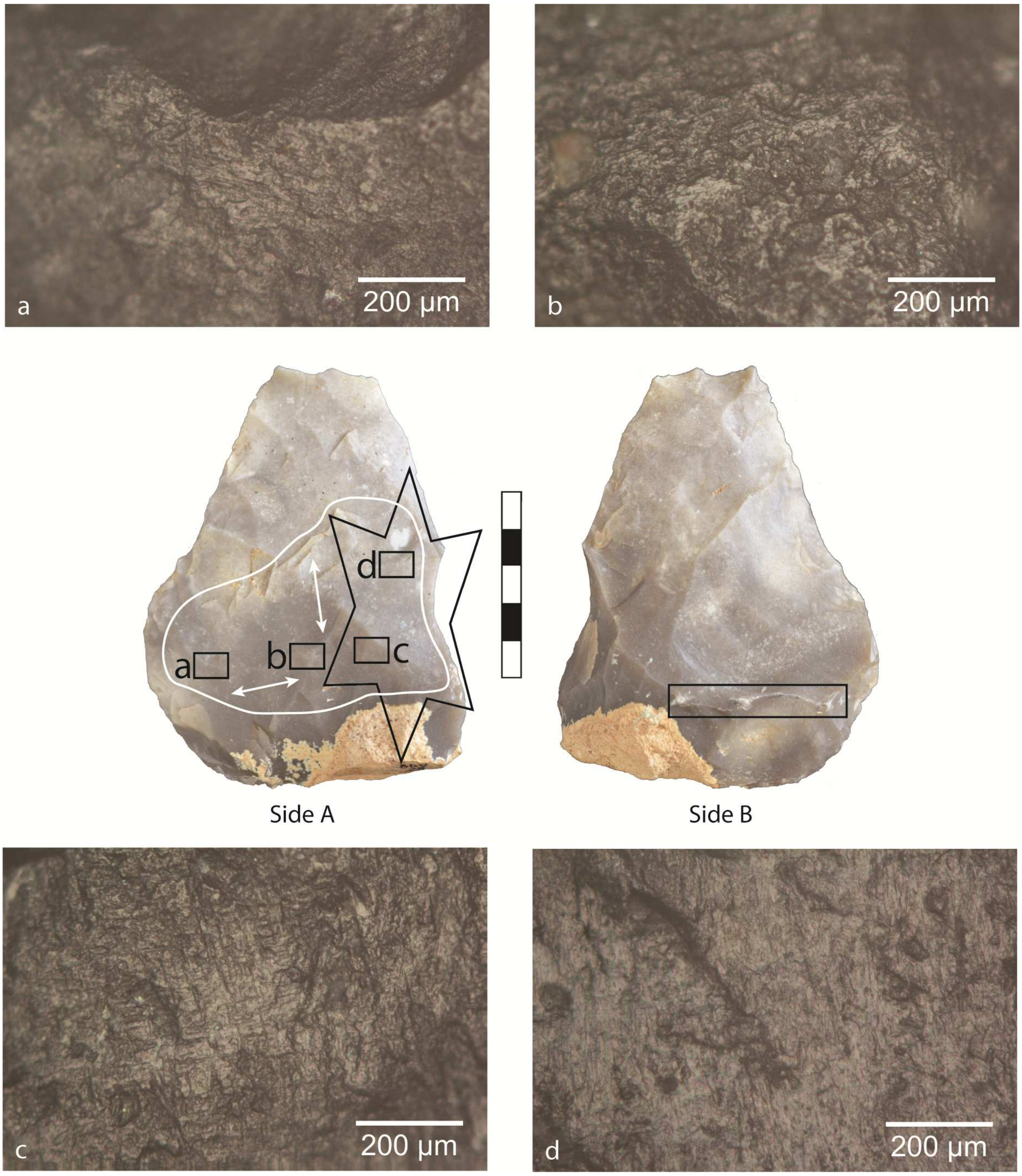

Supplementary Figure S25. Bous-des-Vergnes biface BdV 28 (Dordogne). The white line demarcates the zone where mineral use-wear traces somewhat comparable to pyrite are present. The arrows indicate the orientation of striations. The star indicates the primary zone of percussion, though isolated percussion marks appear throughout the use zone. Black box (Side B) indicates a zone of heavy damage and metallic traces from contact with metal (shovel?). a-d) High-magnification images of mineral polish and striations with perpendicular intersecting directionalities indicating at least two use episodes. This is the only archaeological piece with striations oriented perpendicular to the long axis of the tool.

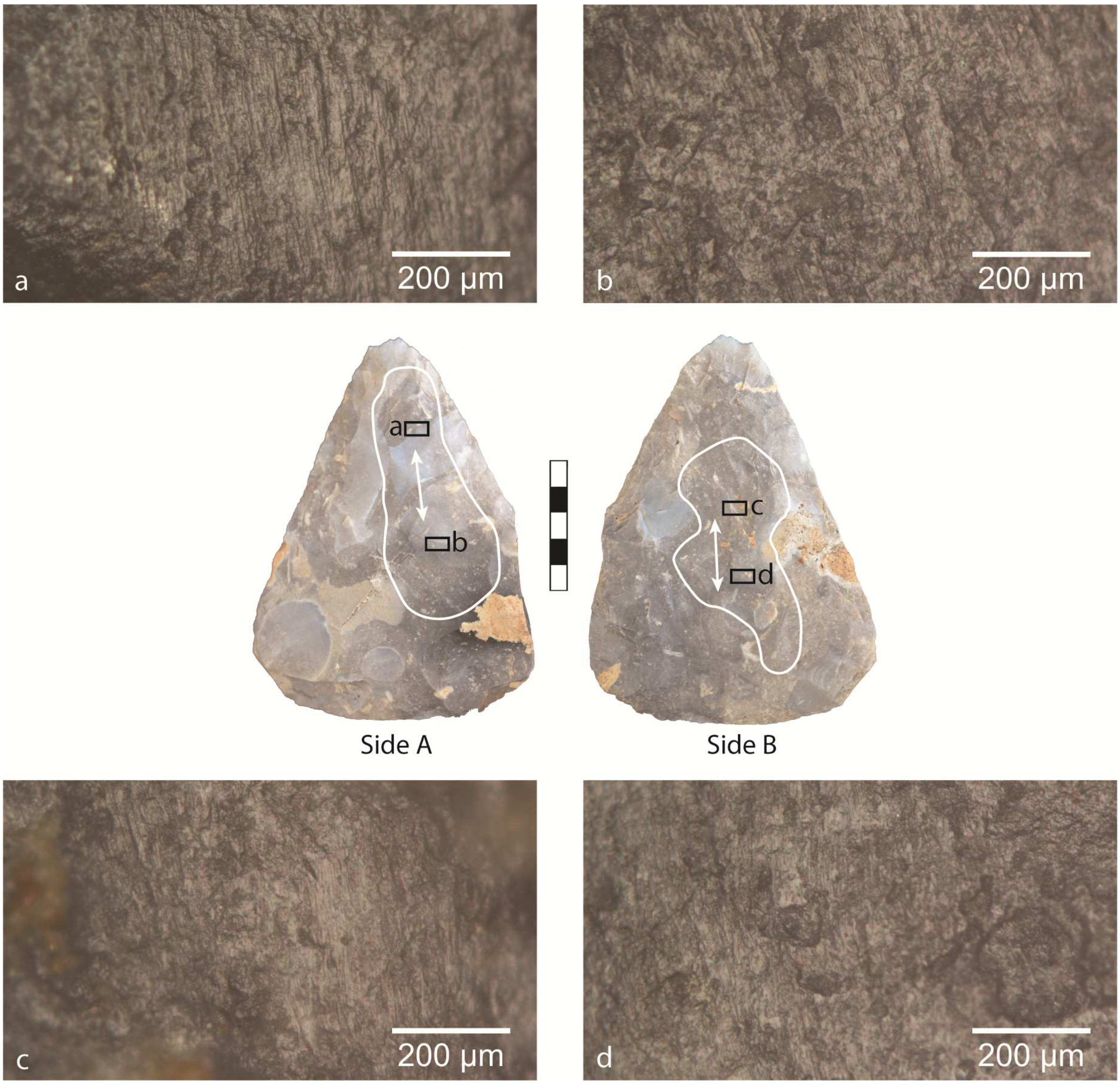

Supplementary Figure S26. Bous-des-Vergnes biface BdV 781 (Dordogne). The white lines demarcate the zones where mineral use-wear traces comparable to pyrite are present. The arrows indicate the orientation of striations. a-d) High-magnification images of mineral polish and striations oriented parallel to the long axis of the biface.

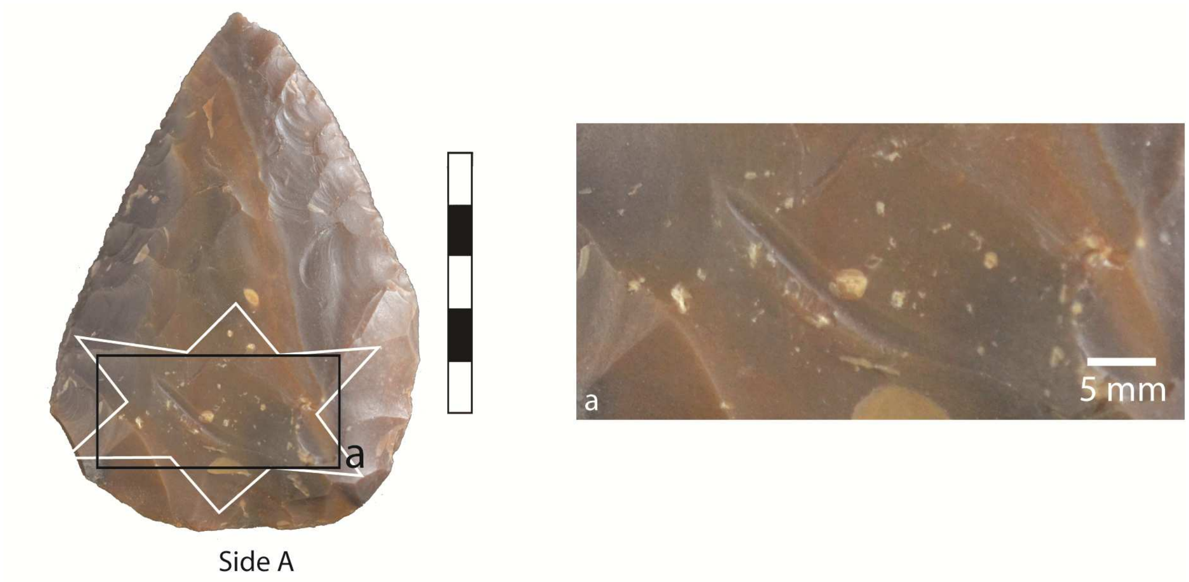

Supplementary Figure S27. Bous-des-Vergnes biface BdV 1651 (Dordogne). The star indicates the zone of percussion. a) Low-magnification images of the gouges in the surface of the flint resulting from retouching/flintknapping the sharp edge of another flint tool.

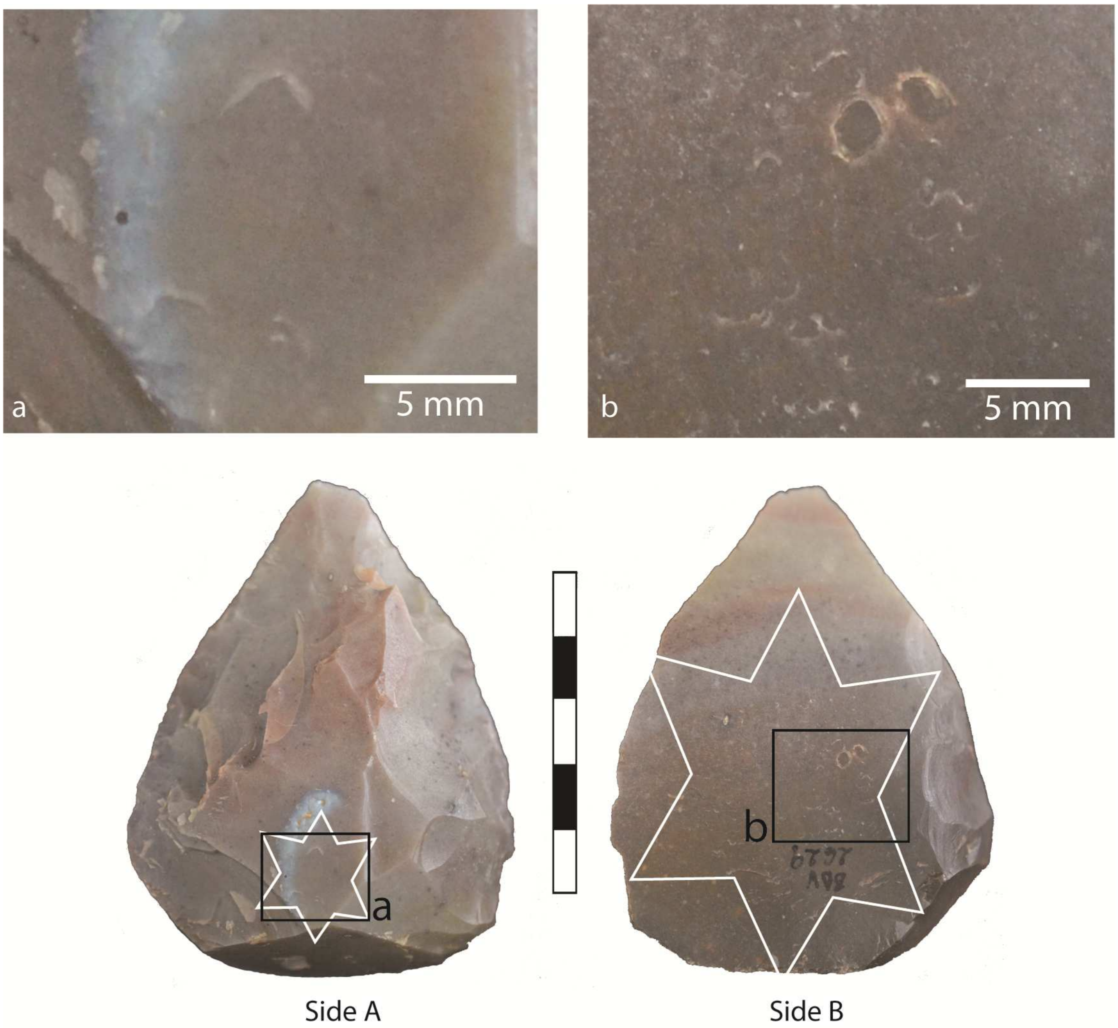

Supplementary Figure S28. Bous-des-Vergnes biface BdV 2629 (Dordogne). The stars indicate zones of percussion. a & b) Low-magnification images of percussion marks likely caused by an unknown pounding activity or flintknapping.

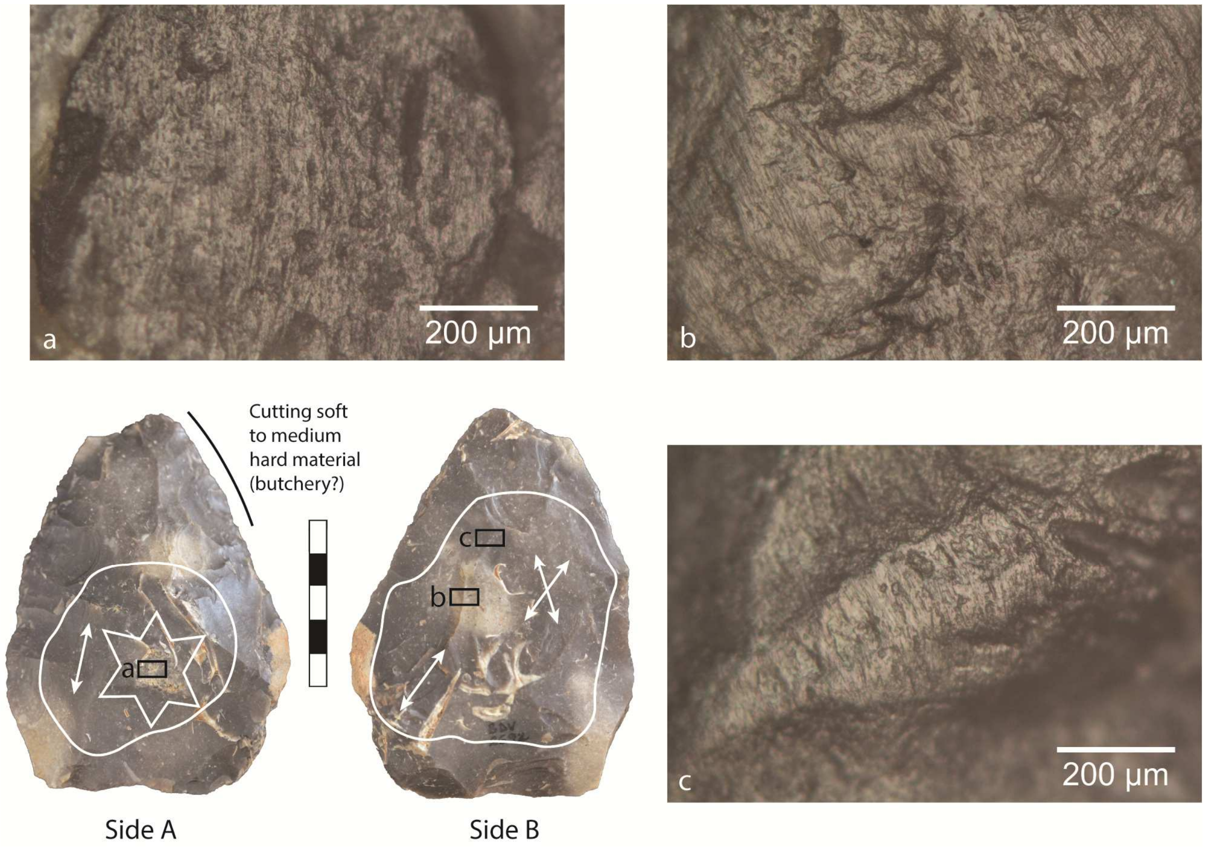

Supplementary Figure S29. Bous-des-Vergnes biface BdV 2692 (Dordogne). The white lines demarcate the zones where mineral use-wear traces comparable to pyrite are present. The star on Side A indicates the primary zone of percussion and heavy crushing, though percussion marks are present throughout use zone. The arrows indicates the orientation of striations. Other observed microwear traces are indicated. a) High-magnification image of mineral microwear traces within a percussion mark fracture. b) High-magnification image of mineral microwear traces with striations showing intersecting directionalities, suggesting more than one use episode. c) High-magnification image of mineral microwear traces on flake scar ridge.

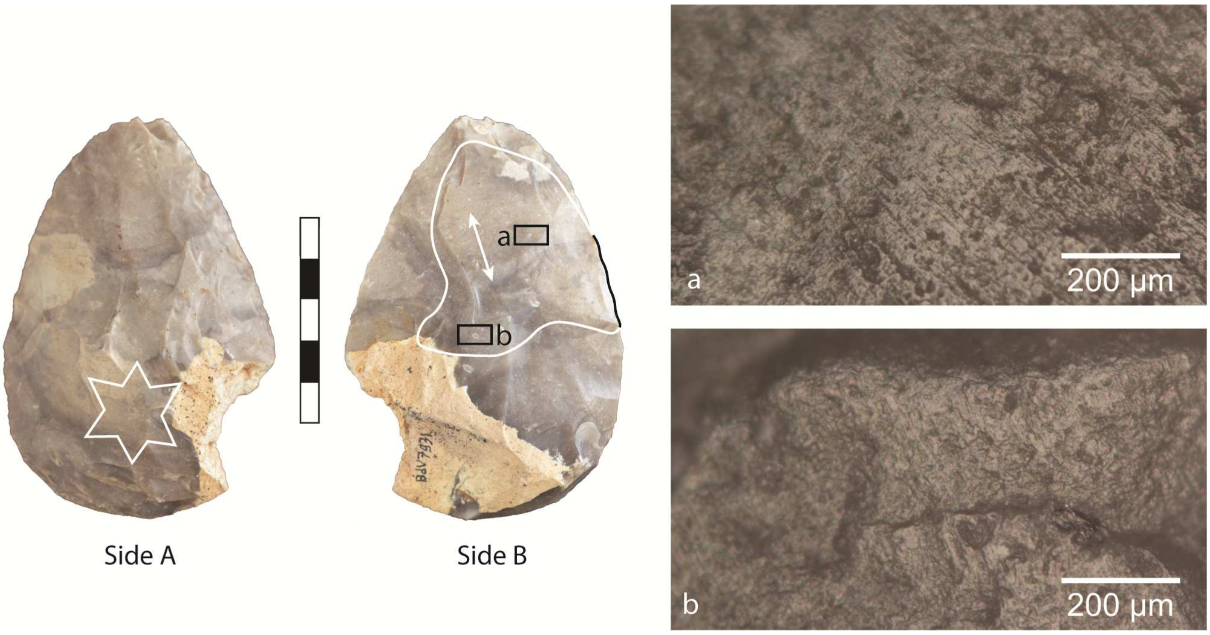

Supplementary Figure S30. Bous-des-Vergnes biface BdV 7931 (Dordogne). The white line on Side B demarcates the zones where mineral use-wear traces are present. The star on Side A indicates a zone of percussion with a few linear gouges, suggesting brief use as a retoucher. The arrow indicates the orientation of striations. a & b) High-magnification images of mineral polish and striations oriented roughly parallel to right lateral edge comparable to pyrite traces.

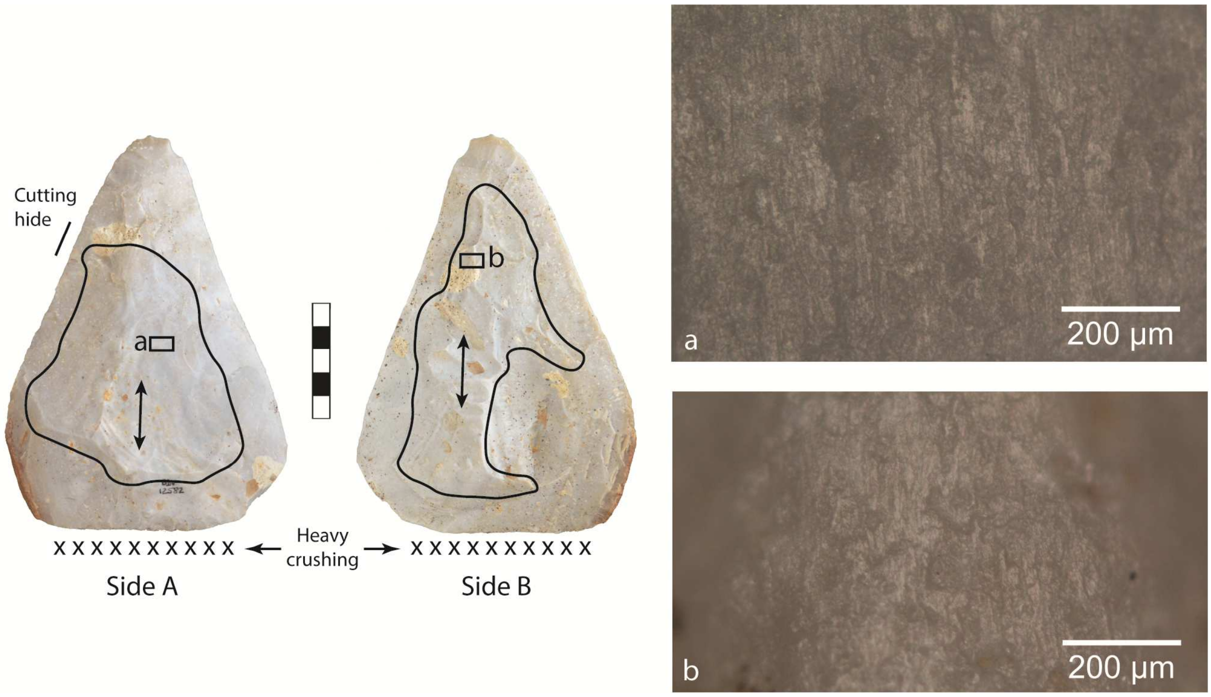

Supplementary Figure S31. Bous-des-Vergnes biface BdV 12582 (Dordogne). The black lines demarcate the zones where mineral use-wear traces are present (notable rounding of flake scar ridges, striations and relatively poorly developed polish). The arrows indicate the orientation of striations roughly parallel to the long axis of the biface. Other observed microwear traces are indicated. a & b) High-magnification images of mineral microwear traces (striations and poorly developed polish) somewhat comparable to pyrite.

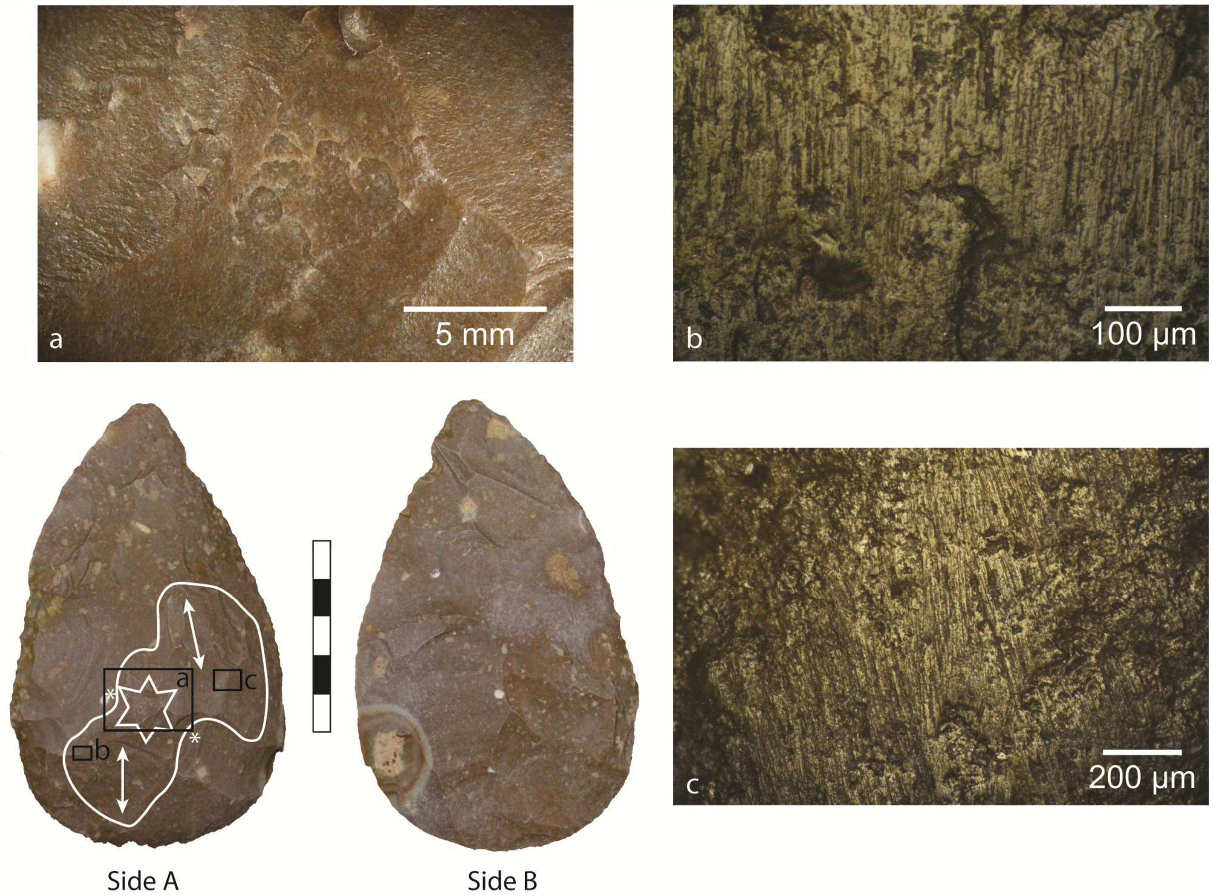

Supplementary Figure S32. Meyrals biface (Dordogne). The white line demarcates the zone where use-wear traces comparable to pyrite are present. The arrows indicate the orientation of striations. The star indicates the zone of percussion marks with ambiguous directionalities, though the majority open proximally. Asterisks indicate zones of percussion marks that have been truncated by subsequent flake removals. a) Low-magnification image of a cluster of percussion marks (center) and truncated percussion marks (left flake negative). b & c) High-magnification images of mineral microwear polish and striations showing slightly variable directionalities, possibly indicating at least two use episodes.

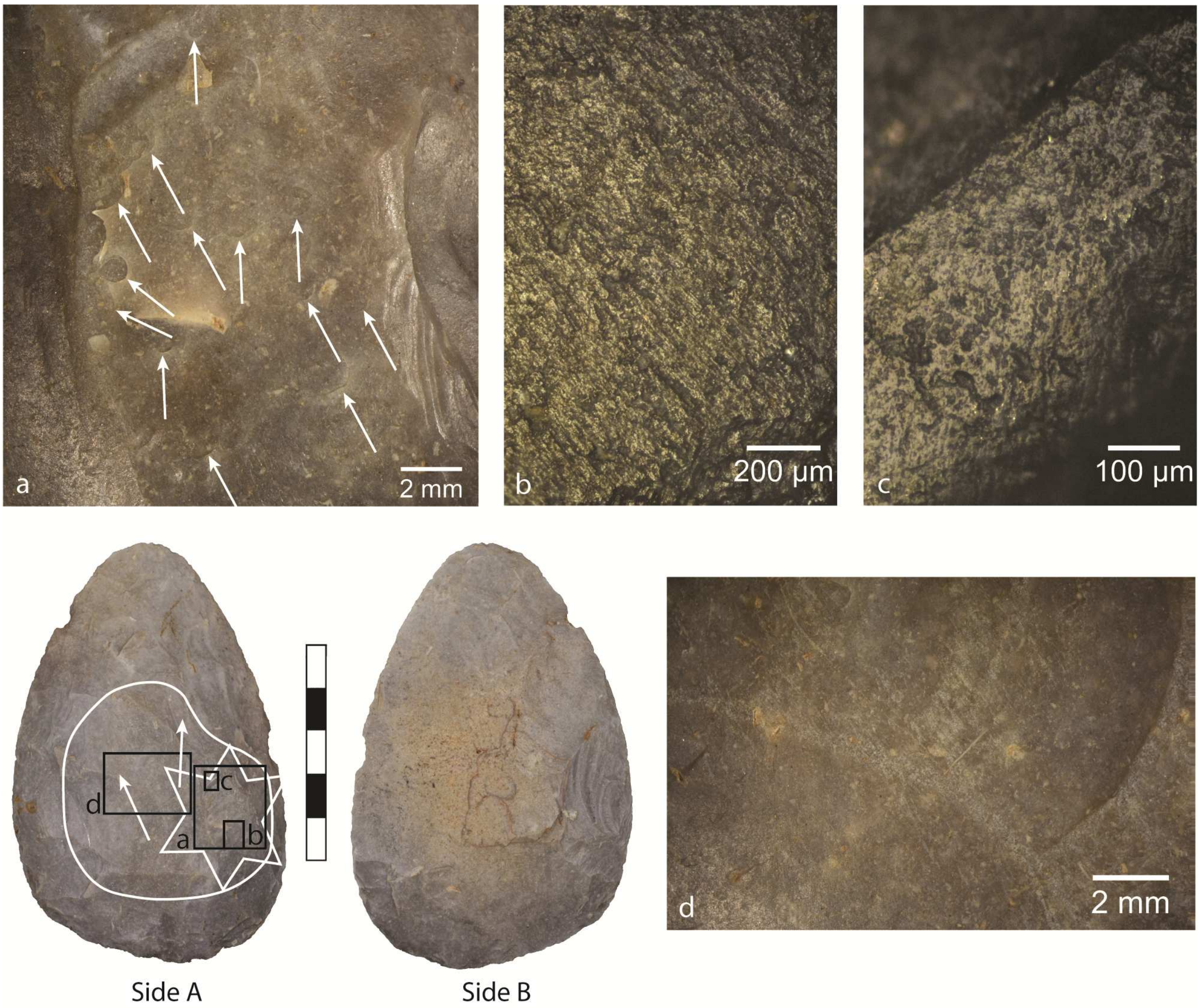

Supplementary Figure S33. Sarlat biface (Dordogne). The white line demarcates the zone where use-wear traces largely similar to pyrite are present. The star indicates the primary zone of percussion, though isolated percussion marks appear throughout the use zone. The arrows indicate the orientation of striations. a) Low-magnification image of a cluster of C-shaped percussion marks and the inferred direction of applied force (arrows). b) High-magnification image of mineral microwear polish (fairly weakly developed) and striations traces oriented roughly parallel to right lateral edge. c) High-magnification image of mineral polish and striations oriented towards distal tip. d) Low-magnification image of moderately worn flake scar ridges associated with lighter colored linear traces that could be attributed to variable patination of additive residues from rubbing a hard siliceous material (e.g. flint).

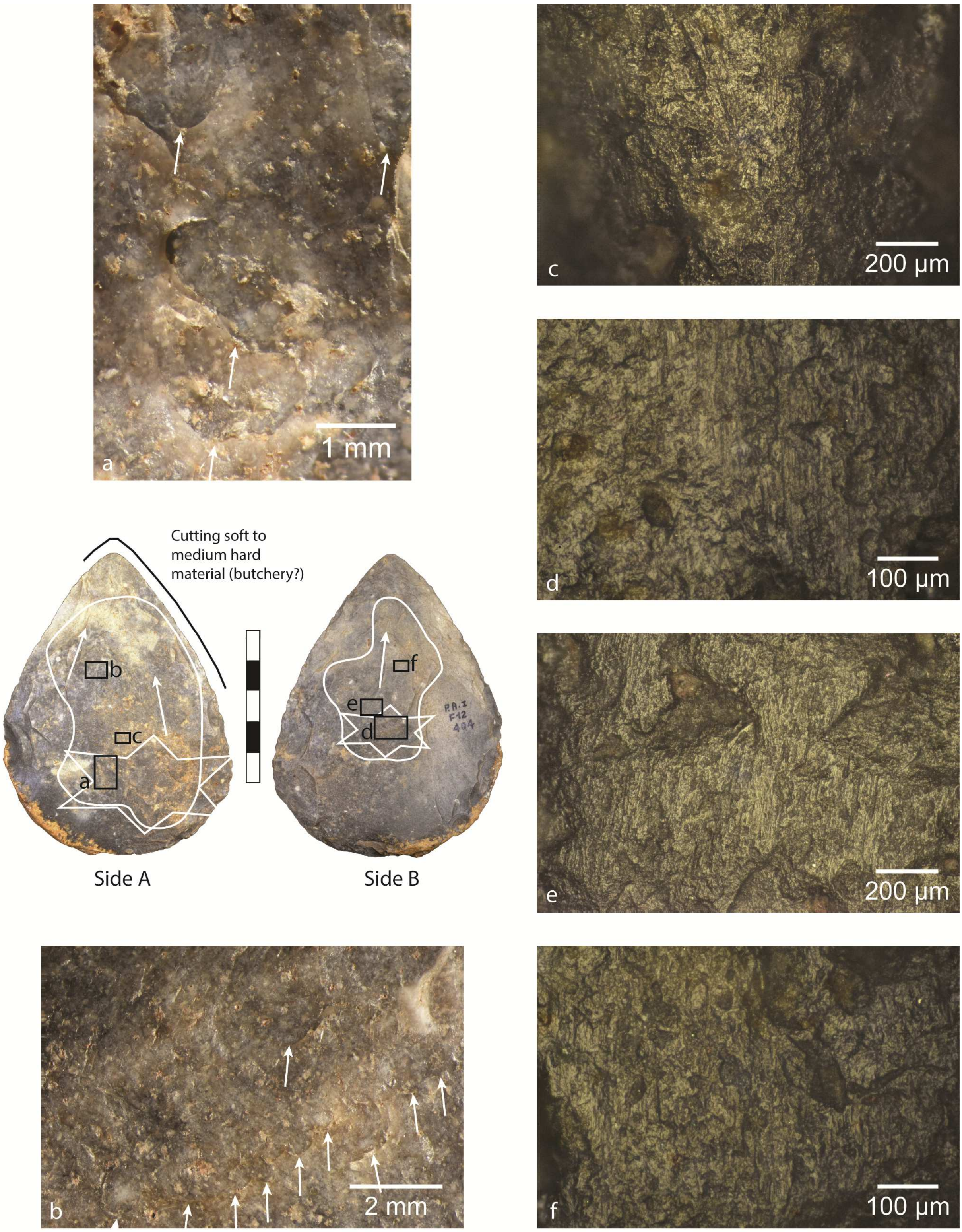

Supplementary Figure S34. Pech de l’Azé I biface PAI F12-404 (Dordogne). The white lines demarcate the zones of mineral use-wear traces comparable to pyrite. The arrows indicate the orientations of the striations, the bi-directional nature of the striations on Side A possibly indicative of at least two episodes of use. The stars encompass zones of percussion on both Side A (a) and B (b) comprised of C-shaped percussion marks opening distally (arrows). The coarser-grained nature of the flint makes these marks particularly difficult to see, especially in the photos. c–f) High-magnification images of mineral polish and striations on Sides A and B.

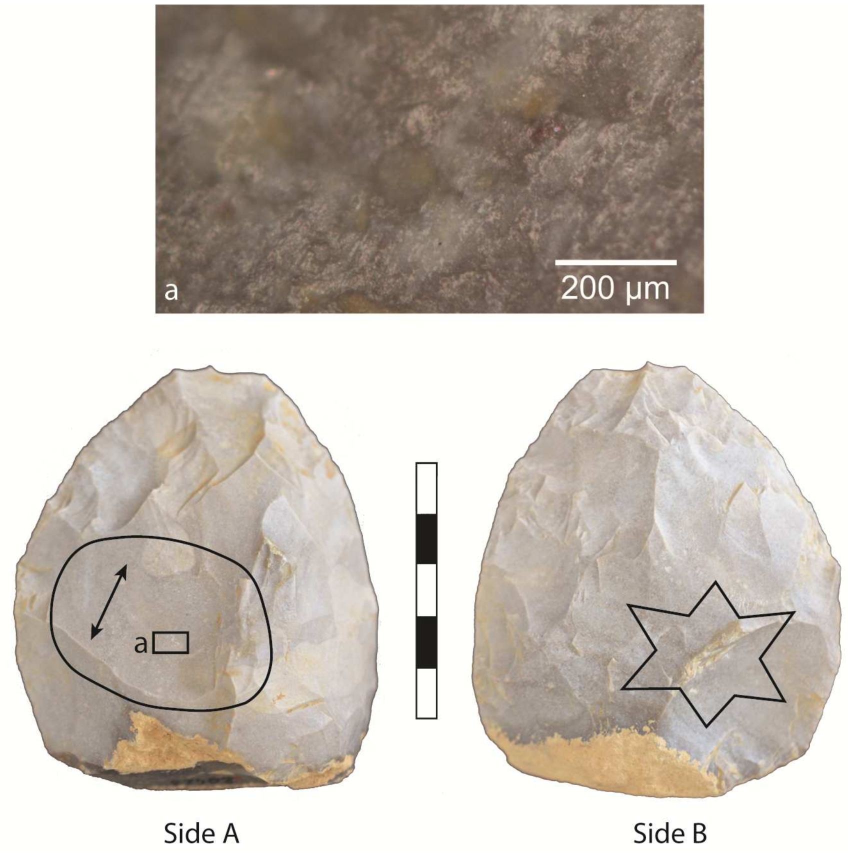

Supplementary Figure S35. Le Prissé biface BP 20423 (Pyrénées Atlantiques). The black line on Side A demarcates the zone where use-wear traces are present (moderate ridge rounding, poorly developed polish and striations and a couple of percussion marks), while the star on Side B indicates a zone of percussion, possibly related to an attempt to use the edges of this deep flake negative as a platform to further thin the biface. The arrow indicates the orientation of striations. a) High-magnification image of unidentified mineral traces (striations, minor polish) associated with an impact mark.

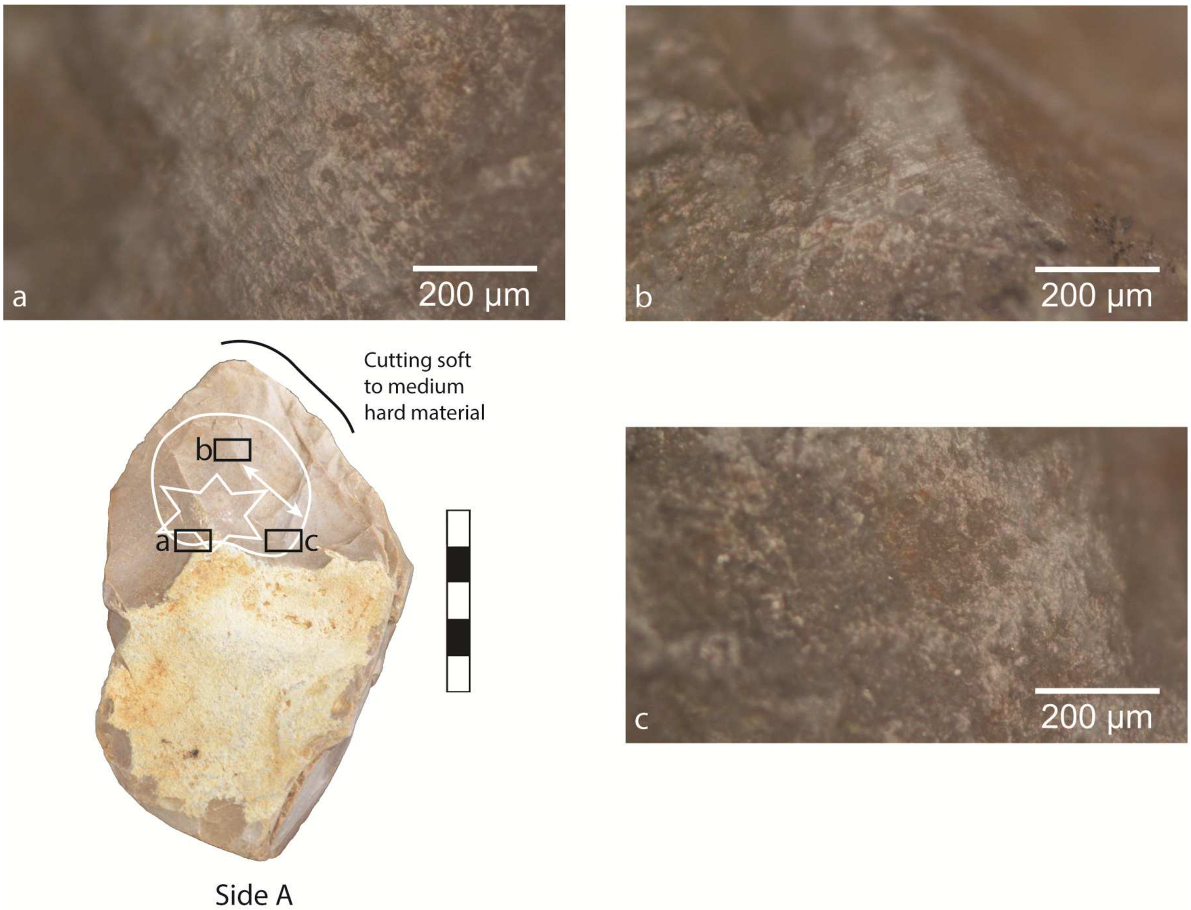

Supplementary Figure S36. Le Prissé biface BP 20746 (Pyrénées Atlantiques). The white line demarcates the zone of relatively poorly developed and ambiguous mineral use-wear traces are present. The arrow indicates the orientation of striations. The star indicates a zone of percussion with moderate crushing. Other observed microwear traces are indicated. a) Mineral polish and striations within a percussion mark. b & c) High-magnification images of unidentified mineral polish and striations. Side B (no traces) is missing due to a corrupted file.

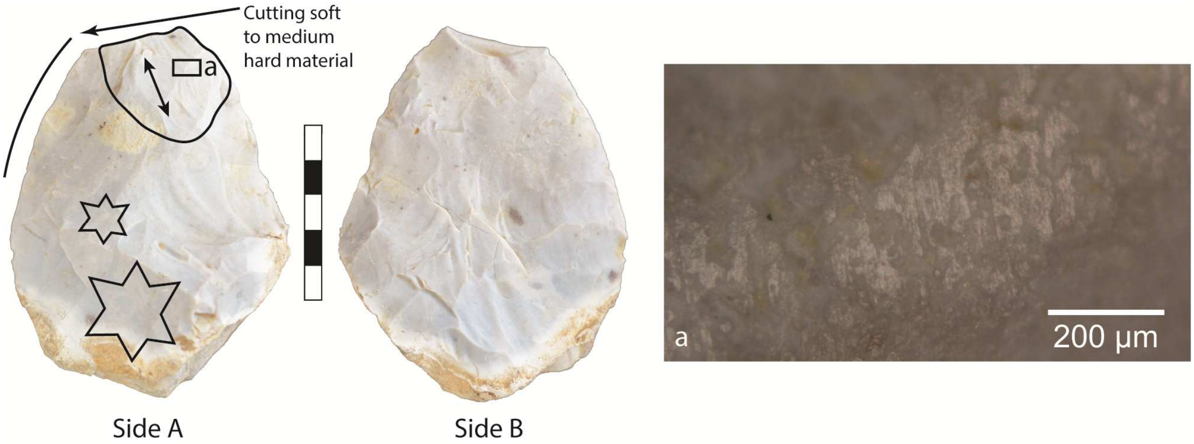

Supplementary Figure S37. Le Prissé biface BPR 10 22302 (Pyrénées Atlantiques). The stars indicate two zones of percussion, each containing a few linear gouges suggesting brief use as a retoucher. The black line demarcates the zone of mineral use-wear traces somewhat similar to pyrite that is apparently unrelated to the percussion zones. The arrow indicate the orientation of striations. Other observed microwear traces are indicated. a) High-magnification image of mineral polish and striations.

Experimental tools.

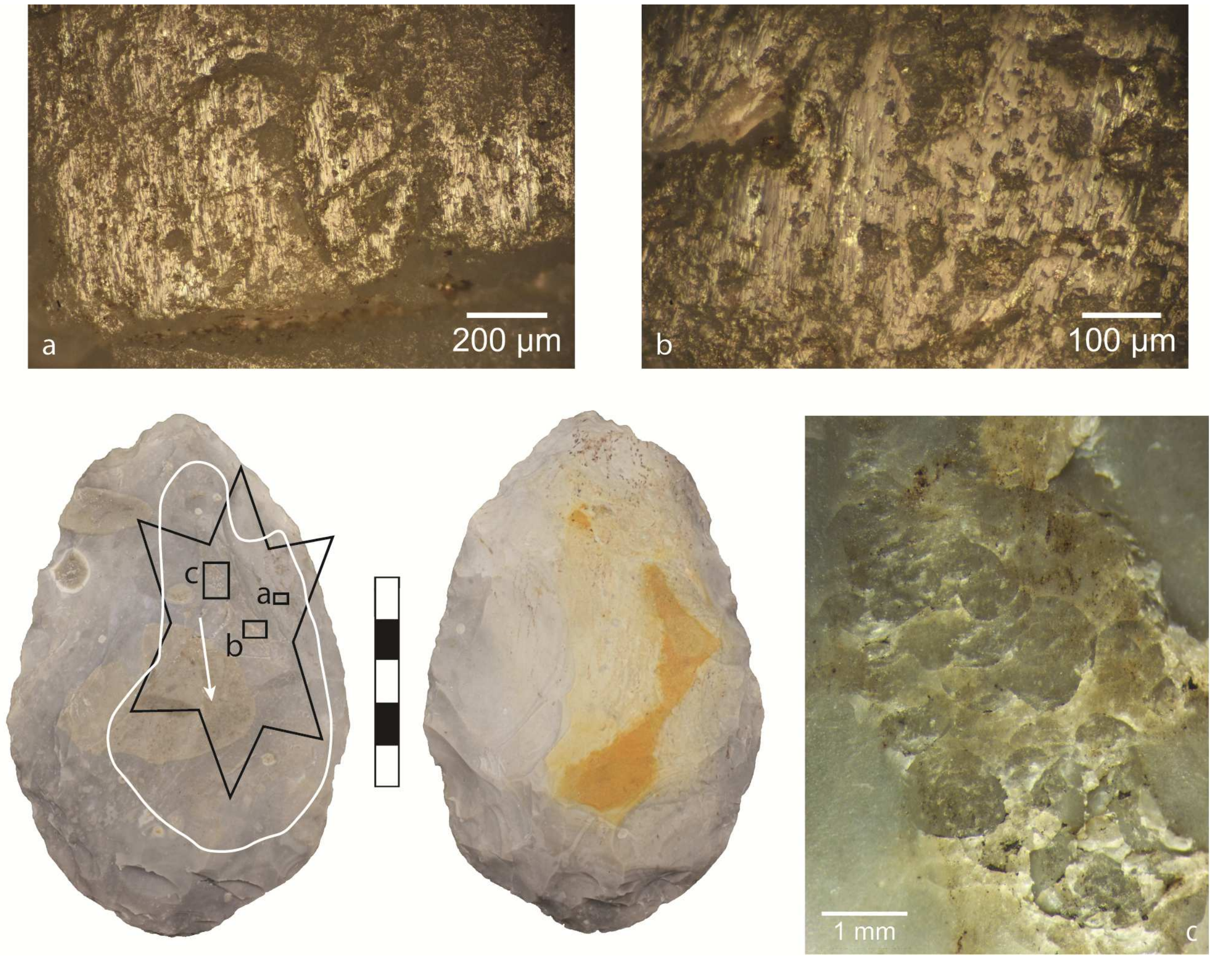

Supplementary Figure S38. Experimental biface 3470, percussed with pyrite nodule fragment for 30 minutes to make fire. The white line demarcates the resultant zone of pyrite use-wear traces. The star indicates the presence of a zone of heavy percussion. The white arrow indicates the direction of force applied by the pyrite (the active element). a & b) High-magnification images of pyrite mineral polish and striations. c) Low-magnification image of a dense cluster of percussion marks and crushing along a flake scar ridge.

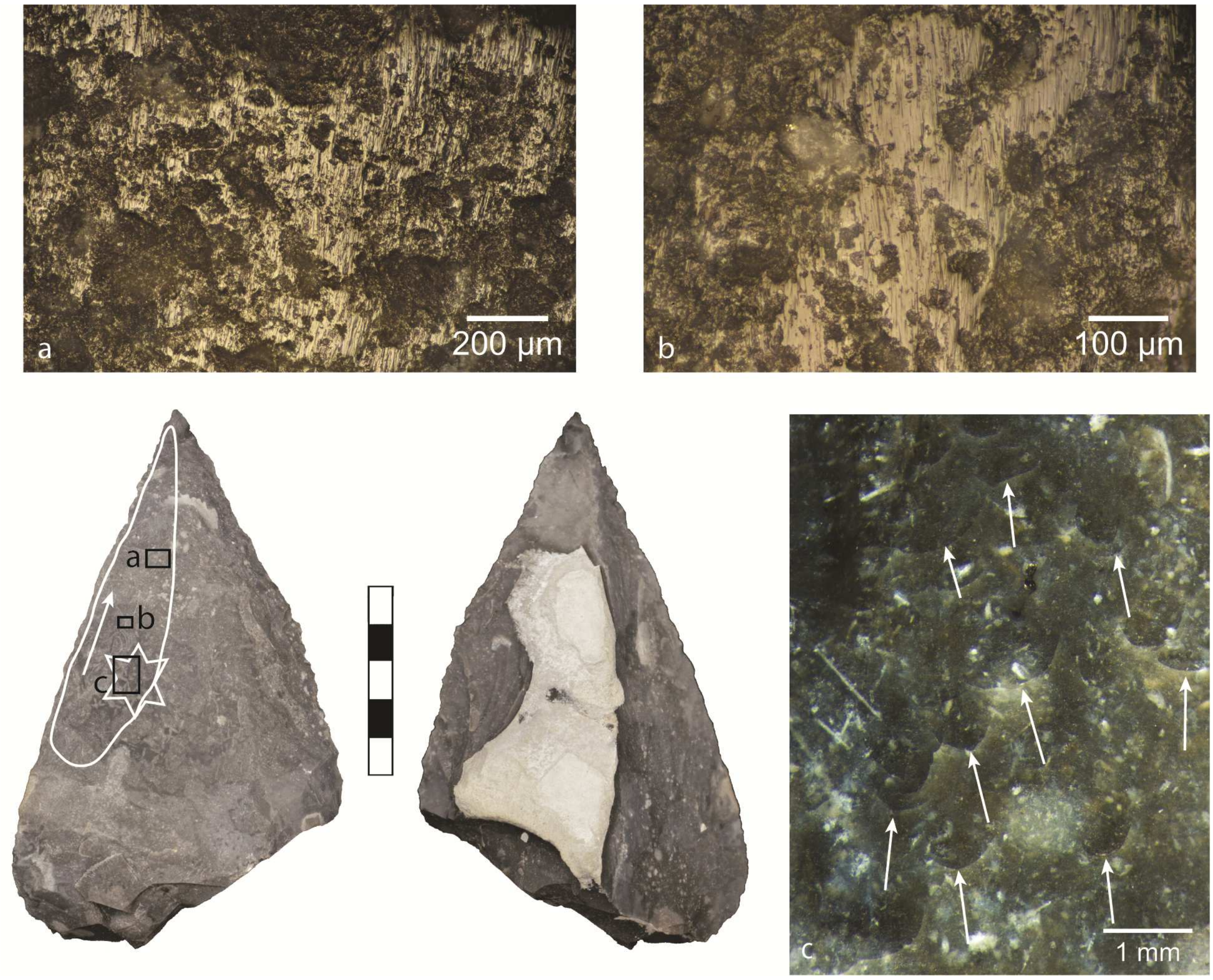

Supplementary Figure S39. Experimental biface 3471, percussed with pyrite nodule fragment for 5 minutes to make fire. The white line demarcates the resultant zone of pyrite use-wear traces. The star delineates a small zone of percussion comprised of C-shaped percussion marks opening distally (c; arrows indicate directionality). The arrow indicates the direction of force applied by the pyrite (the active element). a & b) High-magnification images of pyrite mineral polish and striations. c) Low-magnification image of small C-shaped percussion marks (arrows point to individual percussion marks and indicate the directionality).

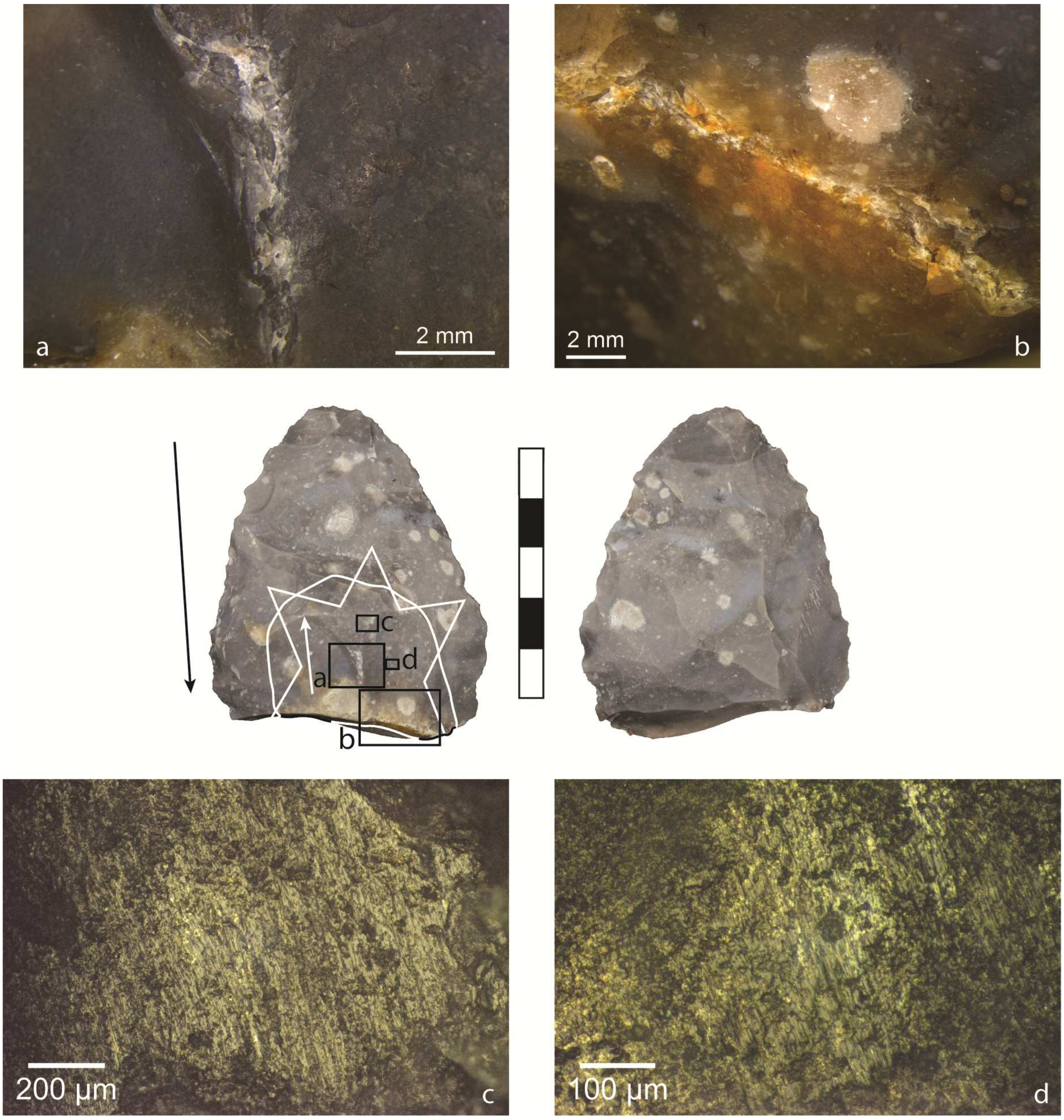

Supplementary Figure S40. Experimental biface 3472, percussed against pyrite nodule fragment 500 times to make fire. The white line demarcates the resultant zone of pyrite use-wear traces. The star indicates the presence of a zone of heavy percussion and crushing. The black arrow indicates the direction of force applied by the biface (the active element) against the pyrite, with the white arrow indicating the direction C-shaped percussion marks open. a) Low-magnification image of percussion marks and crushing along a flake scar ridge. b) Low-magnification image of crushing along the proximal edge of the biface. c & d) High-magnification images of pyrite mineral polish and striations. Gold-coloured, highly reflective areas correspond to pyrite residue remaining after cleaning.

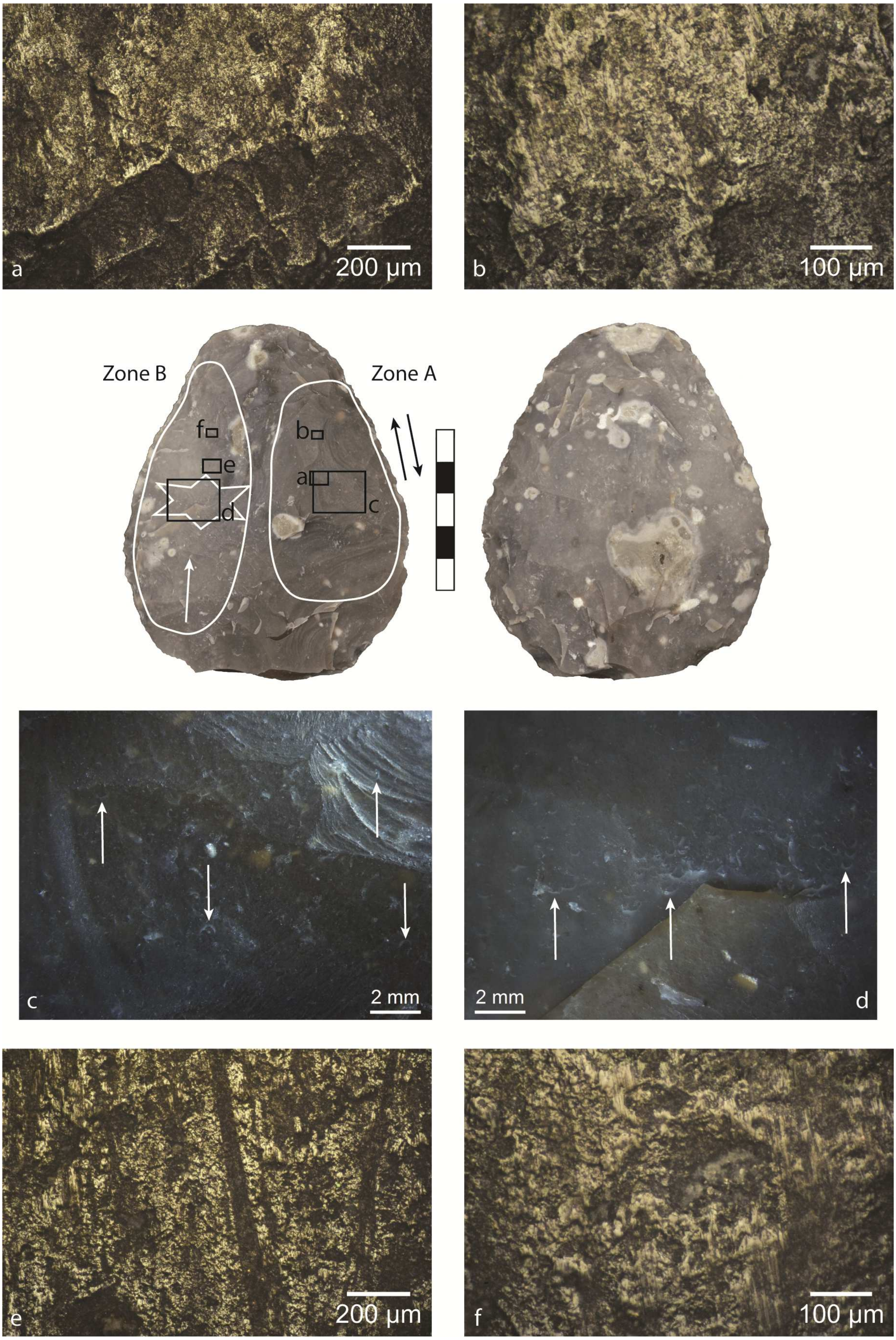

Supplementary Figure S41. Experimental biface 3473 (Side A). Zone A was used to abrade the edge of a large flint biface (passive element) for 5 minutes using a back-and-forth motion (black arrows).

Zone B used as a surface for “backing” the edge of a flint flake (active element), which was forcefully rubbed against the biface for 5 minutes transverse to the flake’s edge (direction of motion indicated by the white arrow). The white lines demarcate the resultant zones of flint use-wear traces. The star indicates the presence of a zone of percussion marks. a & b) High-magnification images of flint mineral polish and weak striations in Zone A. c) Low-magnification image of small, bidirectional C-shaped percussion marks (arrows indicate directionality) in Zone A. d) Low-magnification image of small, unidirectional C-shaped percussion marks (arrows indicate directionality) in Zone B, apparently caused by the sudden change in relief as the flake passed over the step-fracture and dropped onto the lower surface. e & f) High-magnification images of flint mineral polish and striations in Zone B.

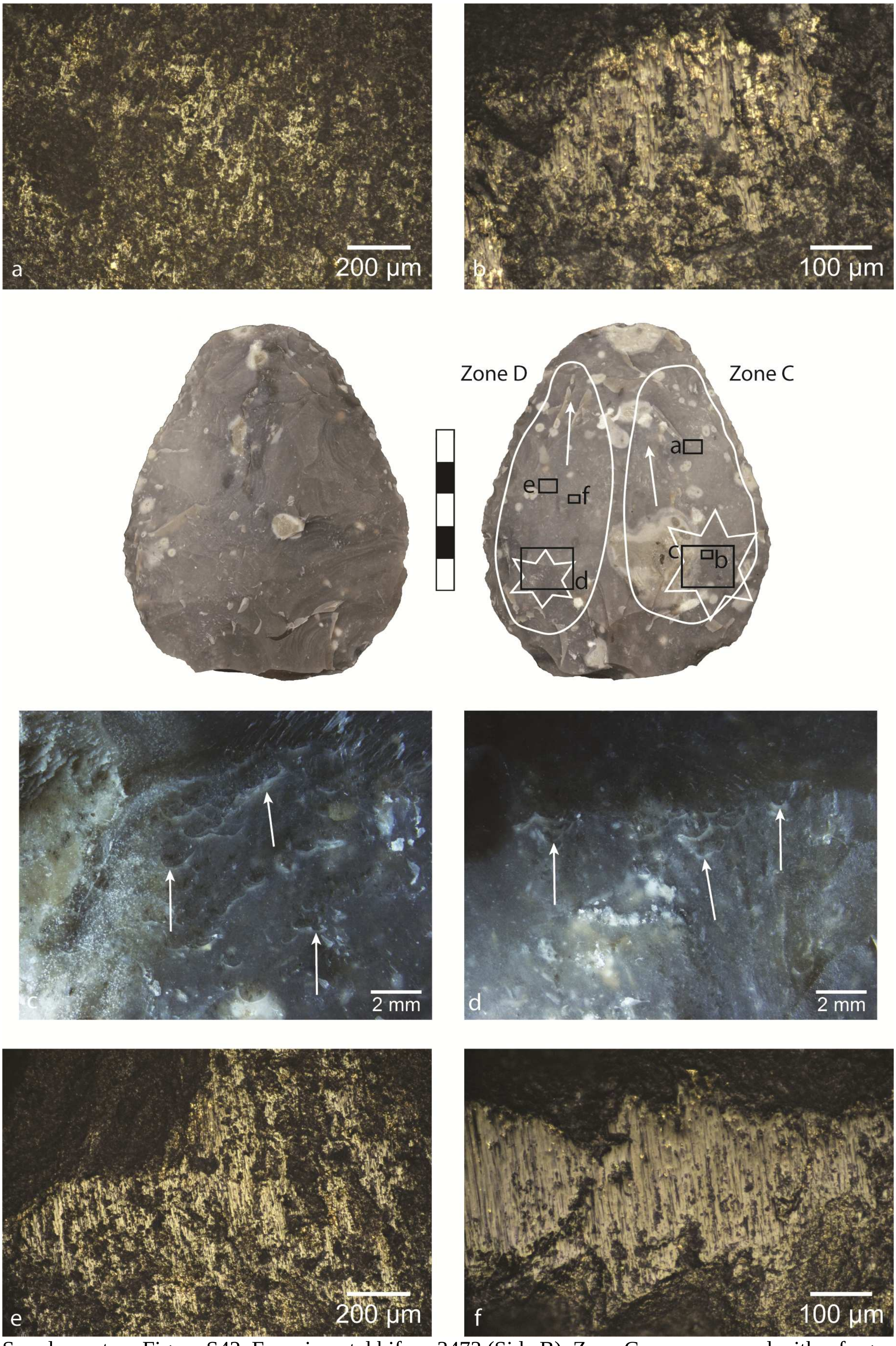

Supplementary Figure S42. Experimental biface 3473 (Side B). Zone C was percussed with a fragment of pyrite crystal aggregate for 5 minutes to make fire. Zone D was percussed with a pyrite nodule

fragment for 2 minutes to make fire. The white lines demarcate the resultant zones of pyrite use-wear traces. The stars indicate clusters of C-shaped percussion marks opening distally (low-magnification images c and d; arrows indicate directionalities). The white arrows indicate the direction of force applied by the pyrite (the active element) to each use zone. a & b) High-magnification images of pyrite mineral polish and striations in Zone A. c) Low-magnification image of small, bidirectional C-shaped percussion marks (arrows indicate directionality) in Zone C. e & f) High-magnification images of pyrite mineral polish and striations in Zone D.

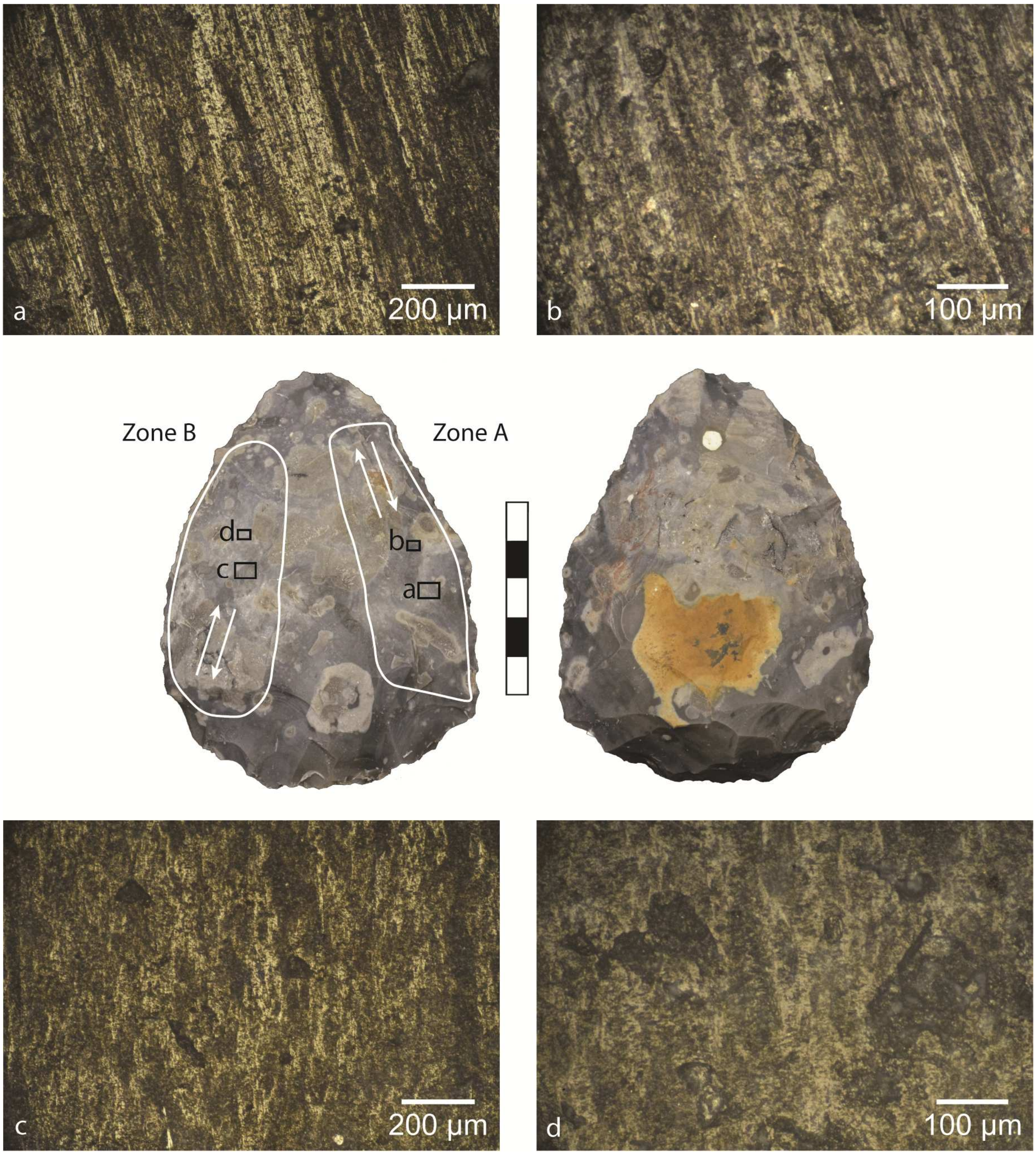

Supplementary Figure S43. Experimental biface 3474 (Side A). Zone A was abraded by a piece of sandstone (active element) for 5 minutes using a back-and-forth motion (white arrows). Zone B was abraded by a piece of river-rounded quartz (neocortex; active element) for 5 minutes using a back-and-forth motion (white arrows). The white lines demarcate the resultant zones of use-wear traces. a & b) High-magnification images of more streaky sandstone mineral polish and striations in Zone A. c & d) High-magnification images of quartz mineral polish and weak striations in Zone B, often exhibiting a more reticulated appearance.

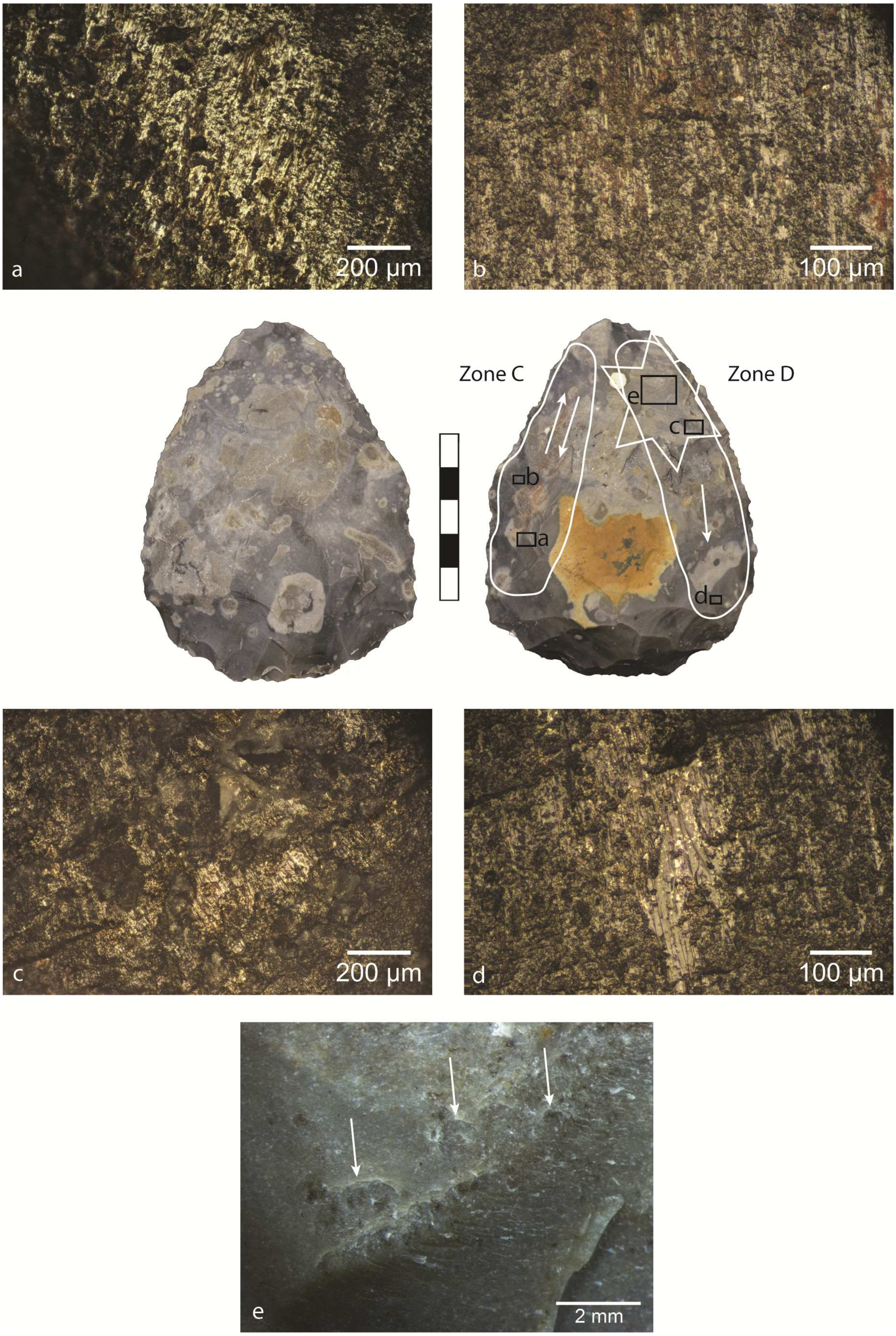

Supplementary Figure S44. Experimental biface 3474 (Side B). Zone C was abraded by a piece of iron-cemented sandstone (active element) for 5 minutes using a back-and-forth motion (white arrows).

Zone D was percussed with a large single euhedral pyrite crystal (active element) for 2 minutes to make fire (the white arrow indicates the direction of force). The white lines demarcate the resultant zones of use-wear traces. The star in Zone D indicates cluster of C-shaped percussion marks opening distally (e; arrows indicated the directionalities of the percussion marks). a & b) High-magnification images of iron-cemented sandstone mineral polish and striations in Zone C. Reddish areas are residues remaining after cleaning. c & d) High-magnification images of pyrite mineral polish and striations in Zone D.

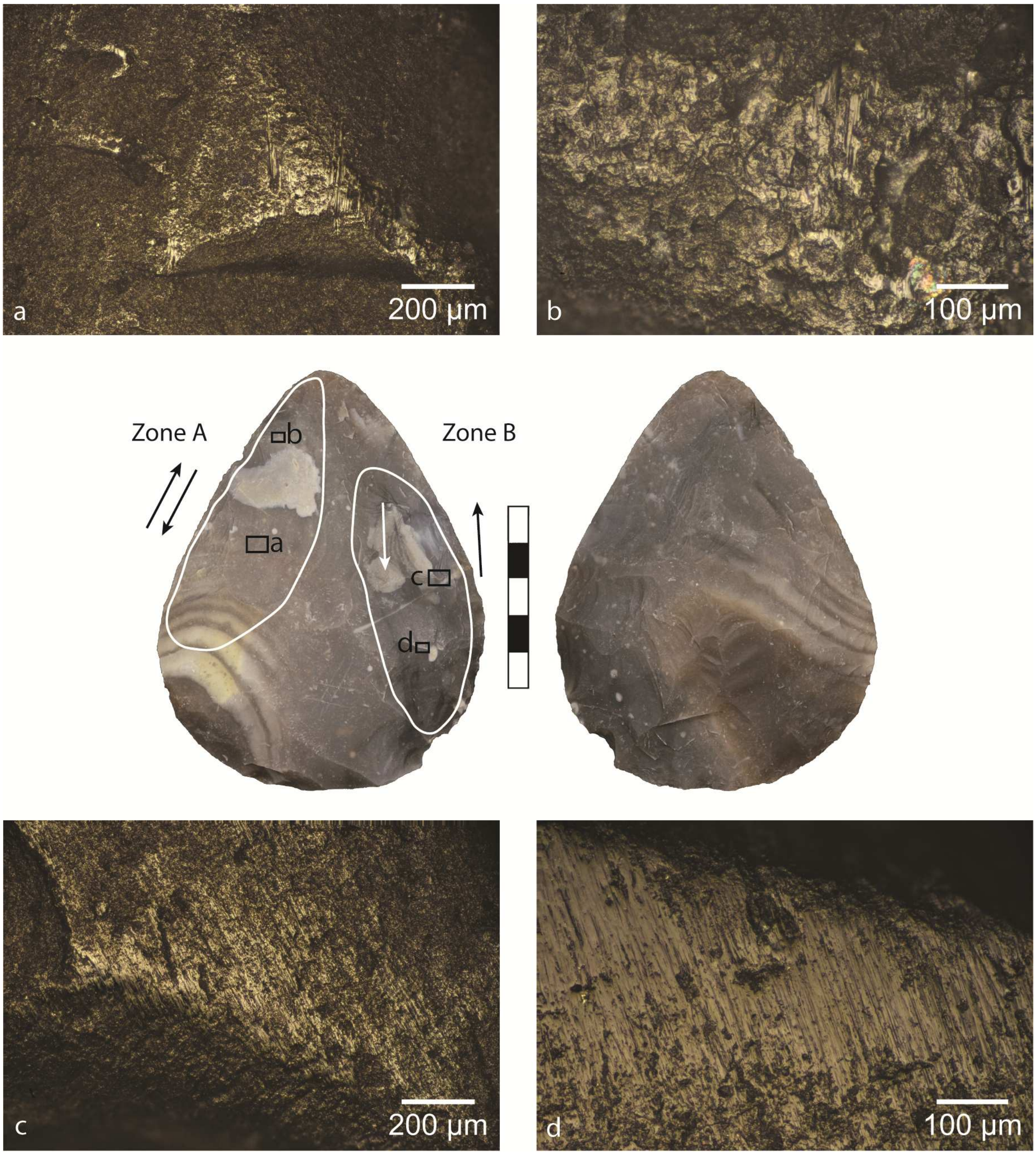

Supplementary Figure S45. Experimental biface 3475 (Side A). Zone A was used to abrade a piece of limestone with some sandy inclusions (passive element) for 5 minutes using a back-and-forth motion (black arrows). Zone B was forcefully rubbed with a halved pyrite nodule fragment 100 times to make fire (the white arrow indicates the direction of motion of the pyrite as it was pushed downward while the black arrow indicates the direction of motion of the biface as it was simultaneously pulled upward). The white lines demarcate the resultant zones of use-wear traces. a & b) High-magnification images of domed limestone mineral polish in Zone A. The deep striations/grooves are likely caused by sandy inclusions. c & d) High-magnification images of pyrite mineral polish and striations in Zone B.

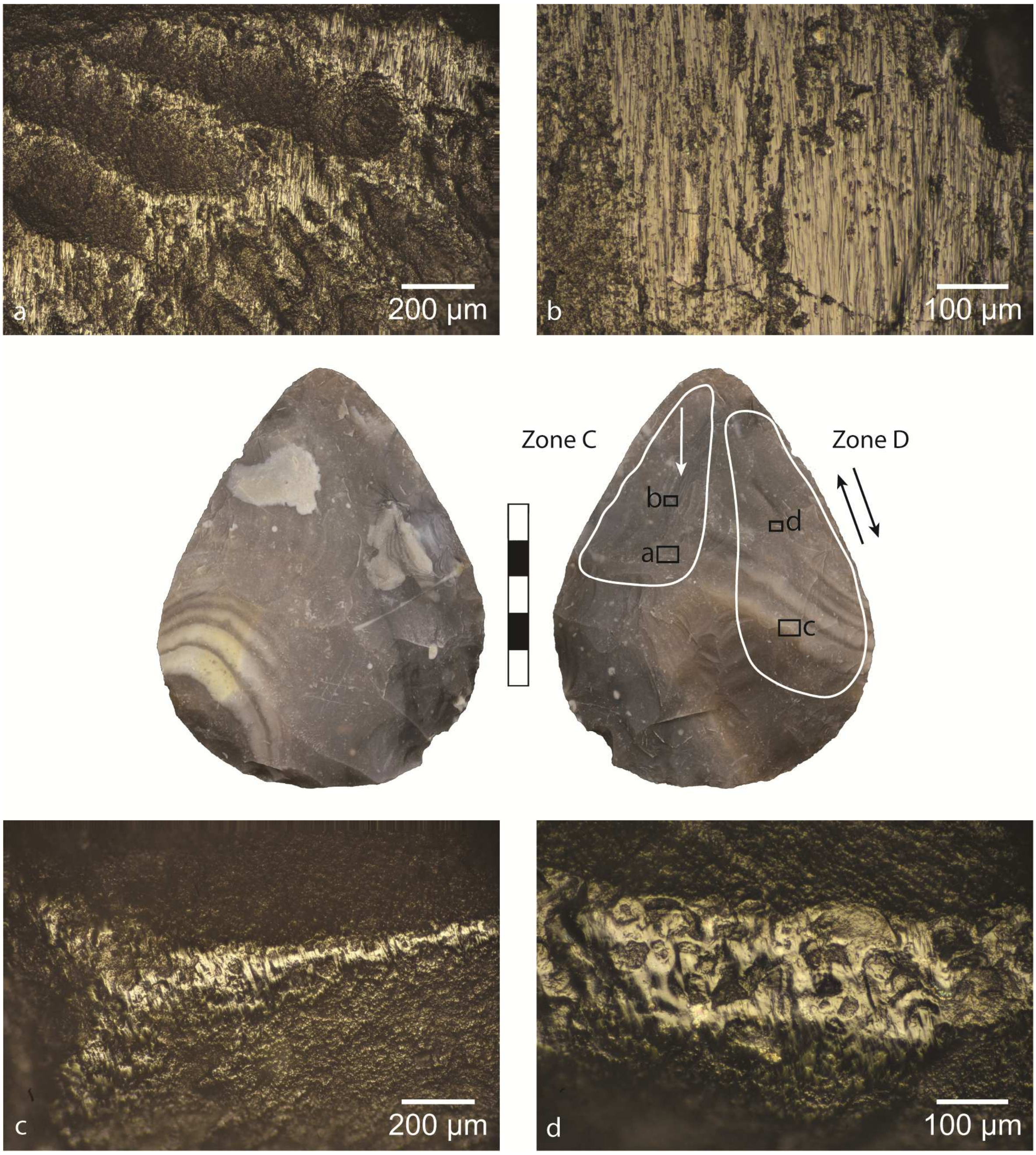

Supplementary Figure S46. Experimental biface 3475 (Side B). Zone C was percussed with a pyrite nodule fragment (active element) for 2 minutes to make fire (the white arrow indicates the direction of force). Zone D was used to abrade a the calcareous cortex of a flint nodule for 5 minutes using a back-and-forth motion (black arrows). The white lines demarcate the resultant zones of use-wear traces. a & b) High-magnification images of pyrite mineral polish, striations and larger scratches along flake scar ridges in Zone C. c & d) High-magnification images of domed limestone mineral polish and weaker, undulating striations in Zone D.

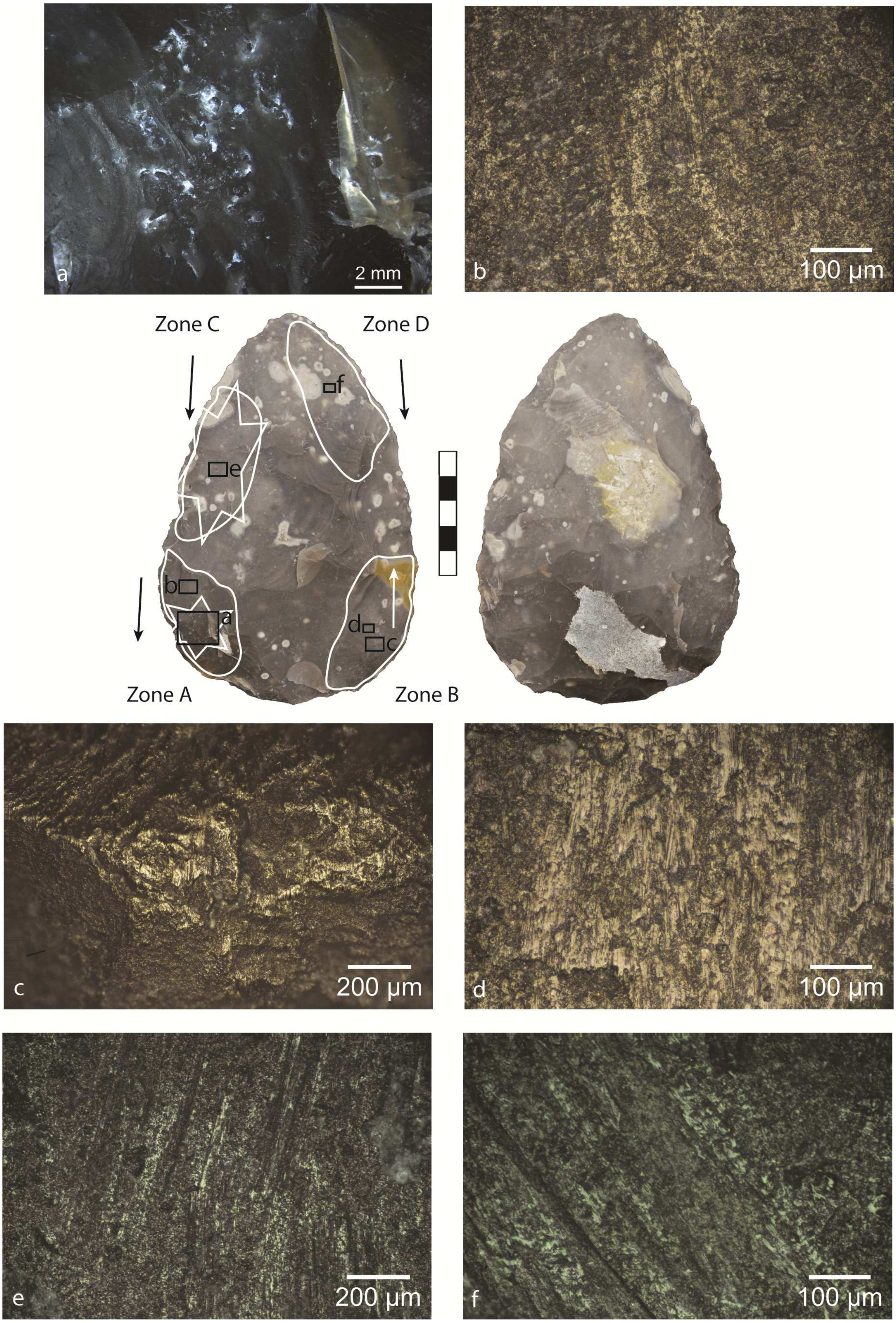

Supplementary Figure S47. Experimental biface 3476 (Side A). Zone A was used to flintknap/retouch a large flint biface (passive element) for 100 strikes. Zone B used as a surface for “backing” the edge of a flint flake (active element), which was forcefully rubbed against the biface 200 times transverse to

the flake's edge (direction of motion indicated by the white arrow). Zone C was used to flintknap/retouch the edge of a quartzite core (passive element) for 100 strikes. Zone D was used to more lightly flintknap/retouch the edge of a different quartzite core (passive element) for 100 strikes. The white lines demarcate the resultant zones of use-wear traces. The black arrows indicate the motion of the biface as it was used for flintknapping/retouching. Stars indicate zones of percussion. a) Low-magnification image of percussion marks and surficial gouging in Zone A. b) High-magnification image of flint mineral polish and weak striations in Zone A. c & d) High-magnification images of weakly reticulated flint mineral polish and striations in Zone B. e & f) High-magnification images of poorly developed quartzite mineral polish and wide, deep grooves in Zones C and D, respectively.

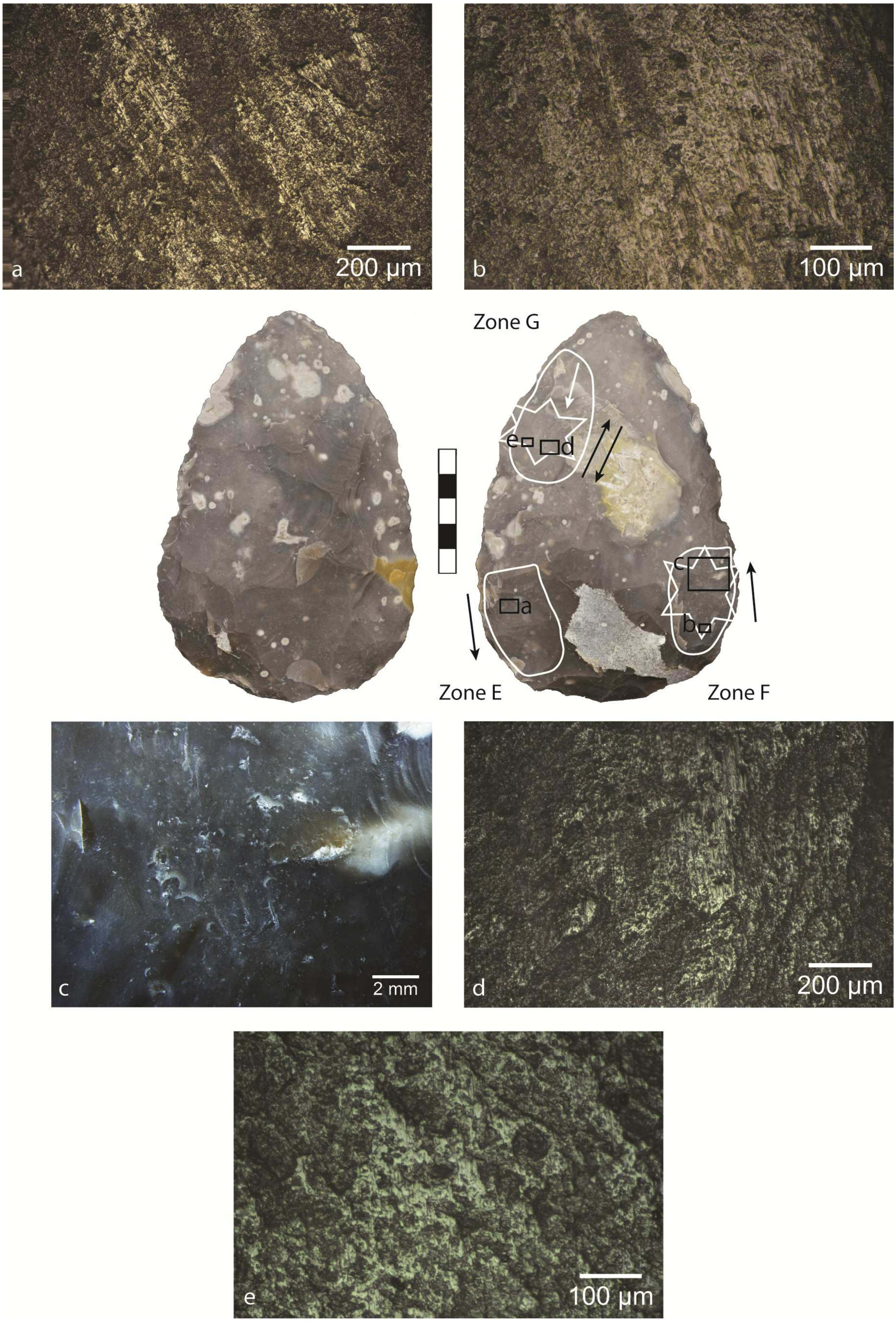

Supplementary Figure S48. Experimental biface 3476 (Side B). Zone E was used to back the edge of a flint blade (passive element), which was forcefully rubbed with the biface 100 times transverse to the blade's edge (direction of biface motion indicated by the black arrow). Zone F lightly retouch the edge

of a large carinated flint scraper (passive element) for 100 strikes (black arrow indicates the motion of the biface). Zone G was initially percussed with a pyrite nodule fragment (active element) for 3 minutes to make fire (the white arrow indicates the direction of force) and then abraded with the edge of a flint core (active element) for 2 minutes using a back-and-forth motion (black arrows). The white lines demarcate the resultant zones of use-wear traces. Stars indicate zones of percussion. a) High-magnification image of flint mineral polish and weak striations in Zone E. b) High-magnification image of flint mineral polish and striations in Zone F. c) Low-magnification images of percussion marks and surficial gouges in Zone F. d & e) High-magnification images of overlapping pyrite and flint mineral polish and striations in Zone G.

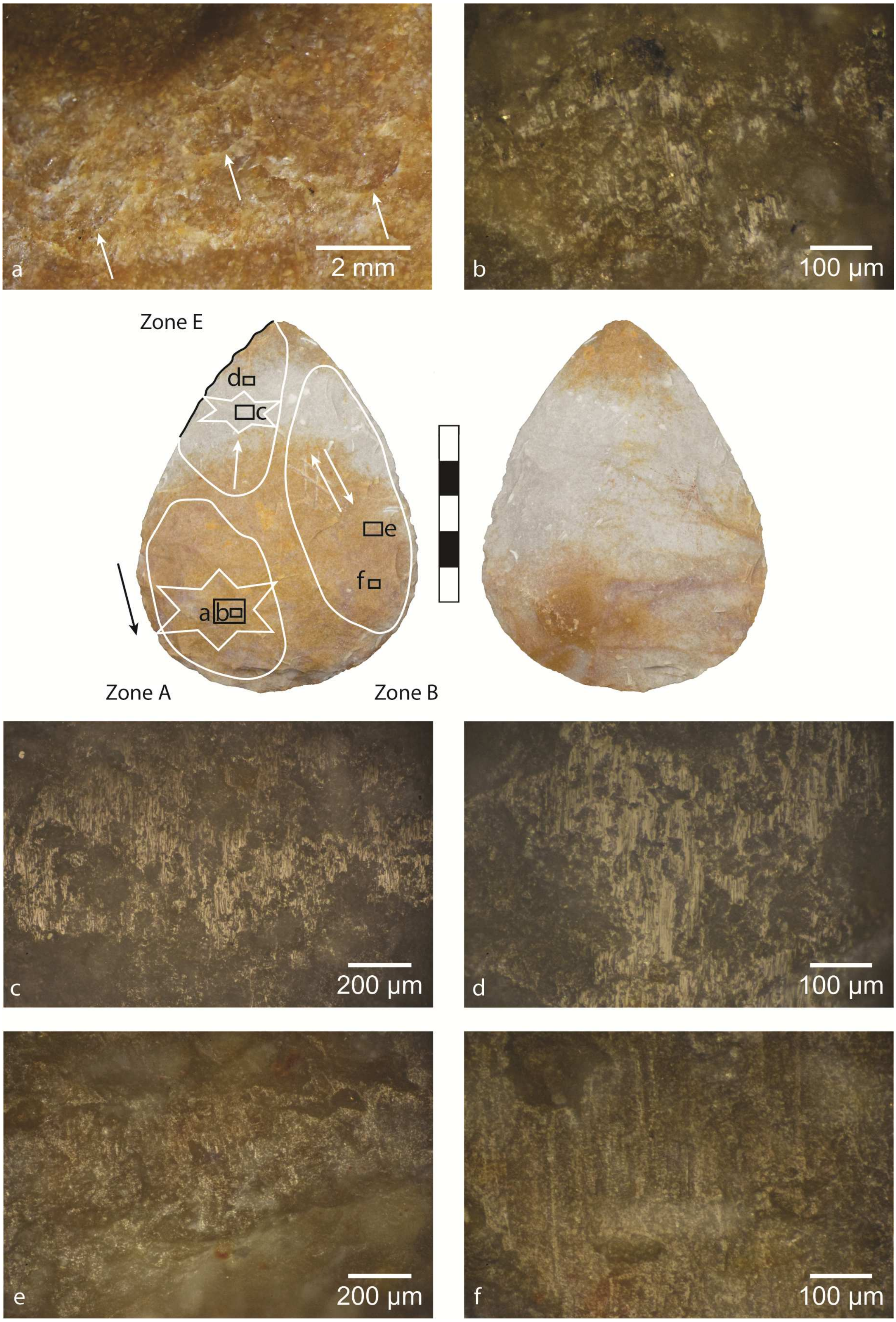

Supplementary Figure S49. Experimental biface 3477 (Side A). Zone A was used to strike a fragment of pyrite crystal aggregate (passive element) for 3 minutes to make fire (black arrow indicates the direction travelled by the biface). Zone B was abraded by a piece of meta-quartz (active element) for 2

minutes using a back-and-forth motion (white arrows). Zone E was percussed with a pyrite nodule fragment (active element) for 5 minutes to make fire (the white arrow indicates the direction of force). The white lines demarcate the resultant zones of use-wear traces. Stars indicate zones of percussion. a) Low-magnification image of distally-opening C-shaped percussion marks (arrows) clustered along a flake scar ridge in Zone A. b) High-magnification image of pyrite mineral polish and striations in Zone A. c & d) High-magnification images of pyrite mineral polish and striations in Zone E. e & f) High-magnification images of somewhat reticulated meta-quartz mineral polish and striations/grooves in Zone B.

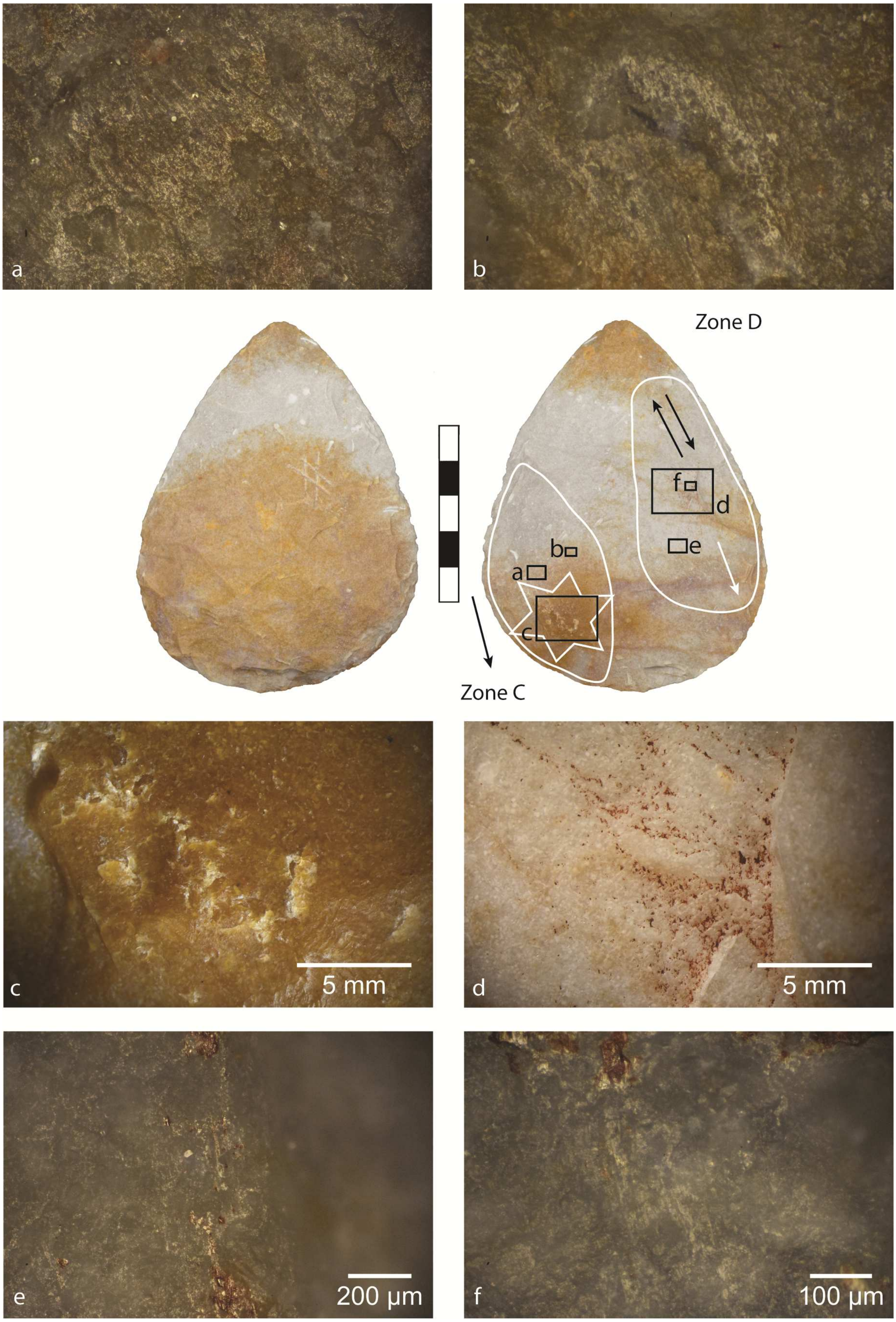

Supplementary Figure S50. Experimental biface 3477 (Side B). Zone D was used to flintknap/retouch a large flint flake (passive element) for around 200 strikes. Zone E was struck 20 times (white arrow indicates the direction of force) and then abraded by a piece of iron-cemented sandstone (active

element) for 1 minute using a back-and-forth motion (black arrows). The white lines demarcate the resultant zones of use-wear traces. The star indicates a zone of percussion in Zone C. a & b) High-magnification images of weak flint mineral polish and striations in Zone C. c) Low-magnification image of percussion marks and surficial gouging in Zone C. d) Low-magnification image of red iron residues remaining in Zone D after cleaning. e & f) High-magnification images of weakly developed iron-cemented sandstone mineral polish and striations and red iron residue in Zone D.

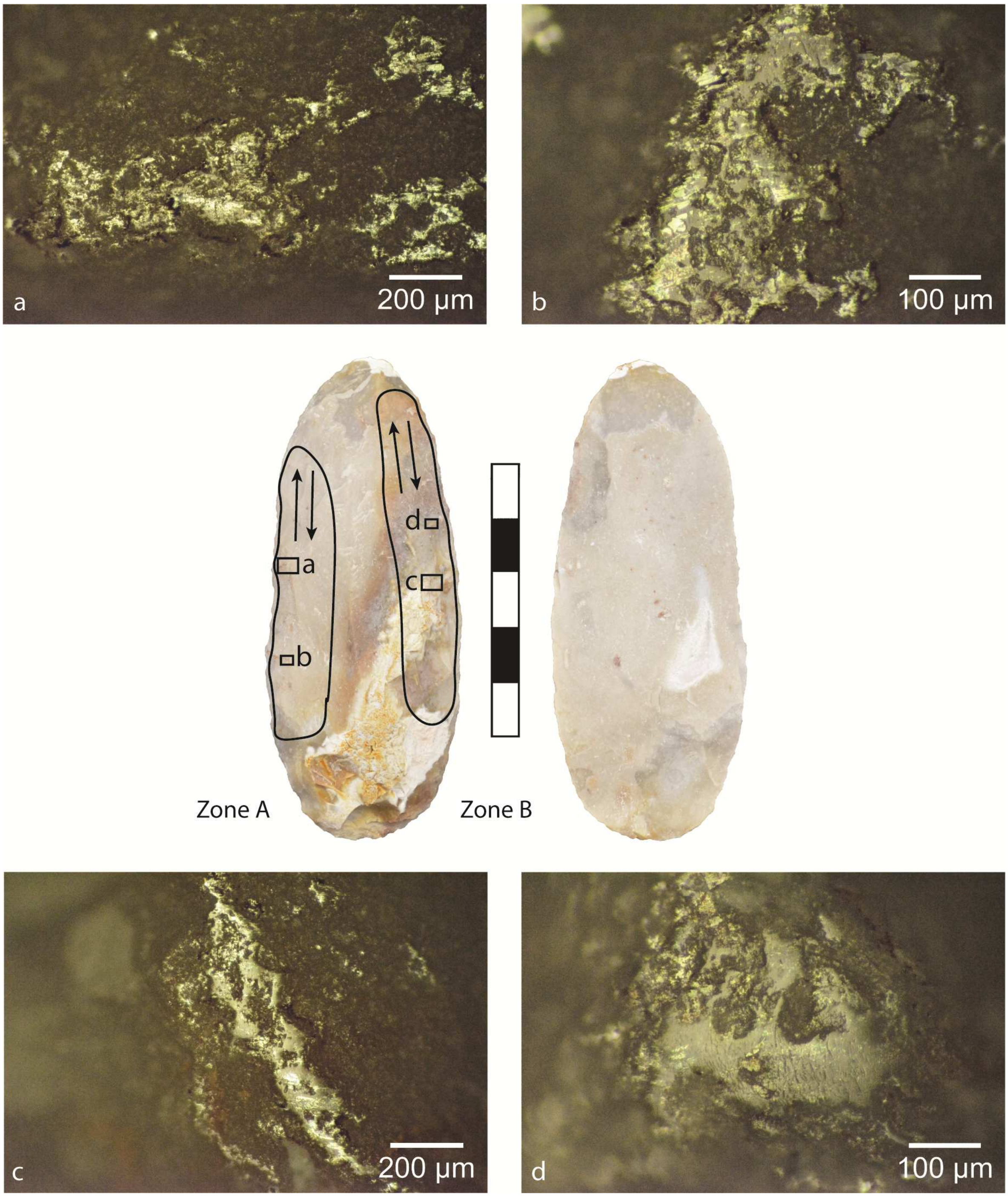

Supplementary Figure S51. Experimental tool 3478 is a unifacial double-scraper, the flaked surface having been used as a grinding surface for a piece of hematite (active element) for 10 minutes (Zone A) and 5 minutes (Zone B) using a back-and-forth motion (black arrows). The black lines demarcate the resultant zones of use-wear traces and red residues. a & b) High-magnification images of hematite mineral polish in Zone A. c & d) High-magnification images of hematite mineral polish in Zone B. The polish is quite bright and tends to lack striations, though undulations in the surface topography can indicate the directionality of the traces, especially at lower magnifications. Fine, recurrent fractures akin to frictive tracks (aka chattersleek) can be seen in image d.

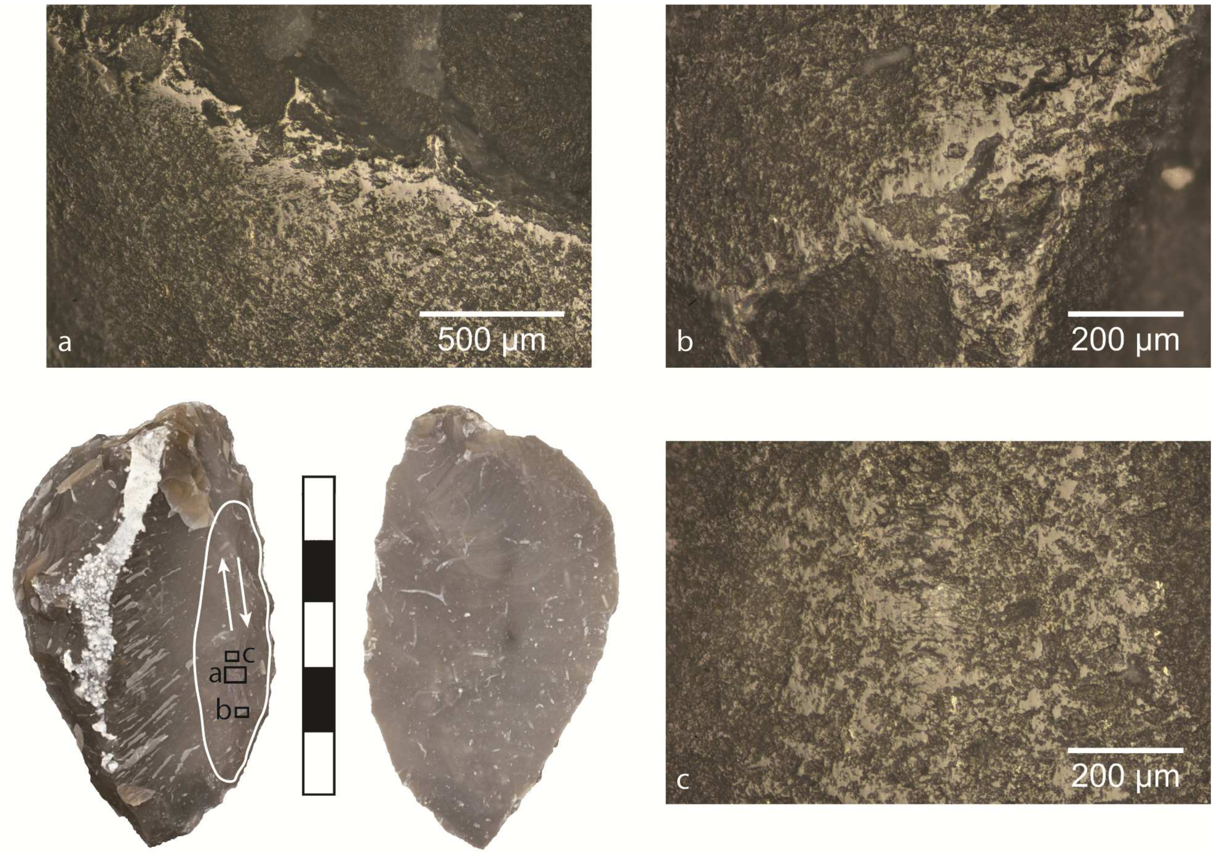

Supplementary Figure S52. Experimental biface 3479 is a unifacial scraper, the flaked surface having been used as a grinding surface for a piece of goethite (active element) for 5 minutes using a back-and-forth motion (white arrows). The white line demarcates the resultant zone of use-wear traces. a –c) High-magnification images of goethite mineral polish. The bright, undulating polish is very similar to hematite, as are the common presence of overlapping frictive tracks, evident in images a and c.

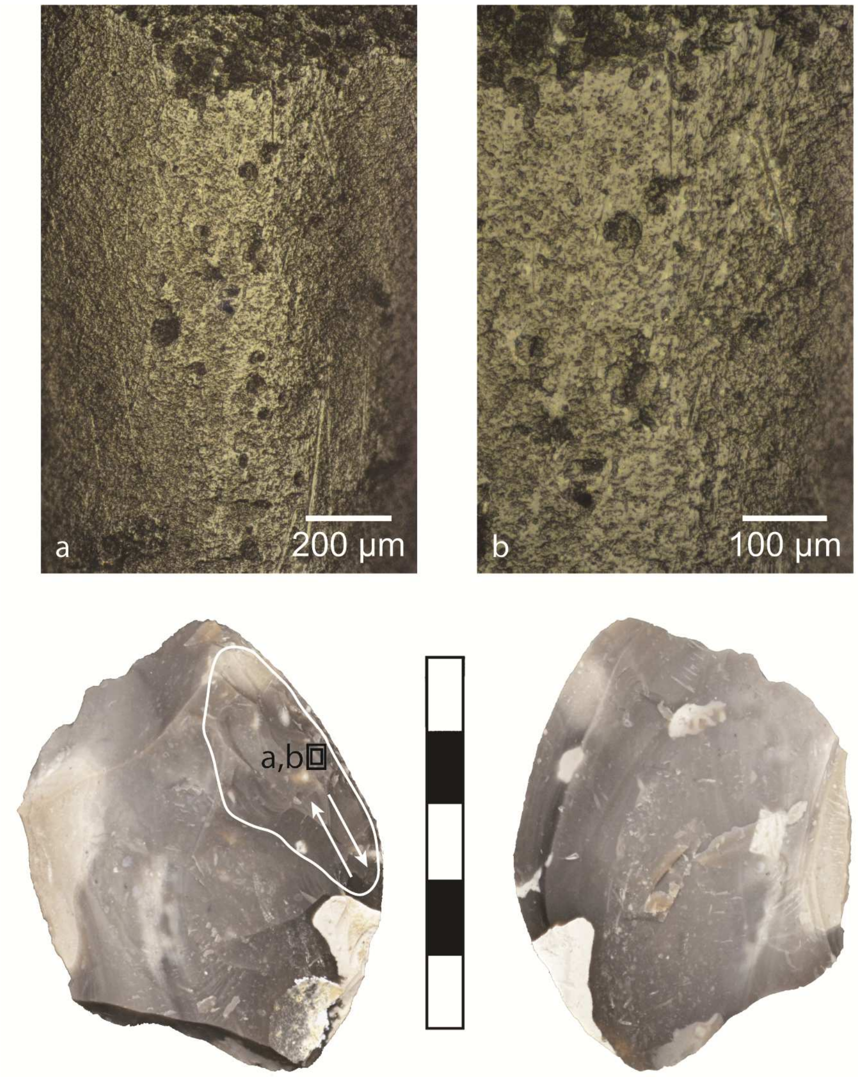

Supplementary Figure S53. Experimental tool 3480 is a unifacial scraper, the flaked surface having been used as a grinding surface for a piece of manganese dioxide (active element) for 5 minutes using a back-and-forth motion (white arrows). The white line demarcates the resultant zone of use-wear traces. a & b) High-magnification images of manganese dioxide mineral polish. The isolated grooves/striations are likely caused by sandy inclusions, which also give the polish a ‘siliceous feel’ akin to flint, quartz or iron-cemented sandstone.

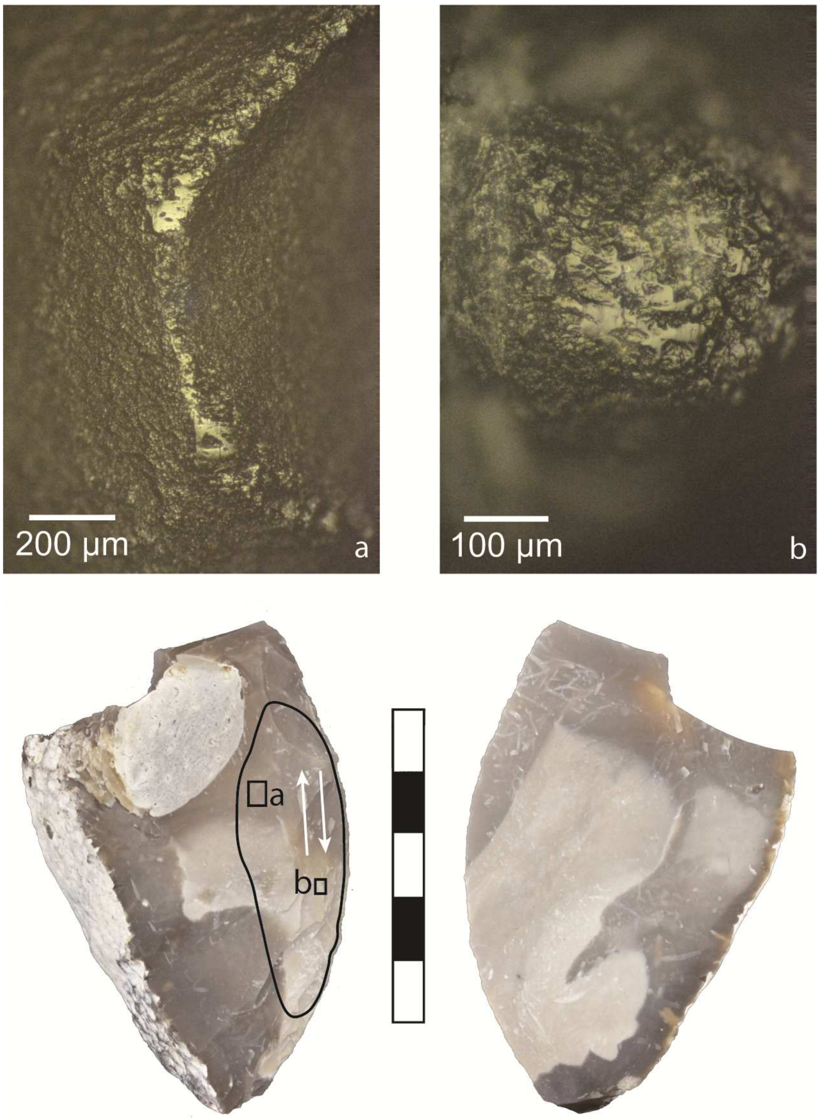

Supplementary Figure S54. Experimental tool 3481 is a unifacial scraper, the flaked surface having been used as a grinding surface for a piece of manganese dioxide intercalated with calcite (active element) for 5 minutes using a back-and-forth motion (white arrows). The black line demarcates the resultant zone of use-wear traces. a & b) High-magnification images of manganese dioxide/calcite mineral polish.

References

1 Claud, E. *Le statut fonctionnel des bifaces au Paléolithique moyen récent dans le Sud-Ouest de la France: Étude tracéologique intégrée des outillages des sites de La Graulet, La Conne de Bergerac, Combe Brune 2, Fonseigner et Chez-Pinaud / Jonzac* PhD thesis, Université Sciences et Technologies - Bordeaux I, (2008).

2 Claud, E. Les bifaces: des outils polyfonctionnels? Étude tracéologique intégrée de bifaces du Paléolithique moyen récent du Sud-Ouest de la France. *Bull Soc Prehist Fr* **109**, 413-439 (2012).

3 Geneste, J.-M. *Analyse lithique d'industries moustériennes du Périgord: une approche technologique du comportement des groupes humaines au Paléolithique moyen* PhD thesis, University of Bordeaux, (1985).

4 Ihuel, E. Le Bout des Vergnes, Bergerac. Dordogne. Aquitaine. Contournement ouest de Bergerac. Rapport final d'opération préventive, CG 24, Service Régional d'Archéologie d'Aquitaine. (in prep.).

5 Brenet, M. *et al.* The function and role of bifaces in the Late Middle Paleolithic of southwestern France: Examples from the Charente and Dordogne to the Basque Country. *Quat Int* **428, Part A**, 151–169 (2017).

6 Soressi, M. *et al.* Pech-de-l'Azé I (Dordogne, France): nouveau regard sur un gisement moustérien de tradition acheuléenne connu depuis le XIX siècle in *Les sociétés Paléolithiques d'un grand Sud-Ouest: nouveaux gisements, nouvelles méthodes, nouveaux résultats* (eds. Jaubert, J., Bordes, J.-G. & Ortega, I.) 95–132 (Société Préhistorique française, 2008).

7 Colonge, D. *et al.* Preliminary results from new Palaeolithic open-air sites near Bayonne (south-western France). *Quat Int* **364**, 109–125 (2015).

8 Feray, P. Saint-Amand-les-eaux (Nord de la France). Rapport Final d'Operation de fouille preventive, INRAP Nord-Picardie, SRA Nord-Pas-de-Calais. (in prep.).

9 Claud, E. New functional data concerning Middle Palaeolithic bifaces from southwestern and northern France in *Proceedings of the International Conference on Use-wear Analysis, Faro, Portugal* (eds Marreiros, J., Bicho, N. & Gibaja, J.F.) Ch. 13, 140–151 (Cambridge Scholars, 2014).

10 Bourguignon, L. Le site stratifié de Canolle Ferme (Creysse). Rapport Final d'Opération préventive, Inrap, Service Régional d'Archéologie d'Aquitaine. (in prep.).

11 Ortega, I. Vieux Coutets (Creysse, Dordogne). Rapport Final d'Opération préventive, Inrap, Service Régional d'Archéologie d'Aquitaine. (in prep).

12 Soressi, M. & Hays, M. A. Manufacture, transport and use of Mousterian bifaces. A case study from the Perigord (France) in *Multiple approaches to the study of bifacial technologies* (eds. Soressi, M. & Dibble, H.) Ch. 6, 125–147 (The University of Pennsylvania Museum Press, 2003).

13 Soressi, M. *Le Moustérien de tradition acheuléenne du sud-ouest de la France. Discussion sur la signification du faciès à partir de l'étude comparée de quatre sites: Pech-de-l'Azé I, Le Moustier, La Rochette et la Grotte XVI* PhD thesis, Université Bordeaux I, (2002).

14 Niekus, M. J. L. T. *et al.* News from the north: A late Middle Palaeolithic site rich in handaxes on the Drenthe–Frisian till plateau near Assen, the Netherlands; first results of a trial excavation. *Quat Int* **411**, 284–304 (2016).
